# Deficits of hierarchical predictive coding in left spatial neglect

**DOI:** 10.1093/braincomms/fcab111

**Published:** 2021-06-04

**Authors:** Fabrizio Doricchi, Mario Pinto, Michele Pellegrino, Fabio Marson, Marilena Aiello, Serena Campana, Francesco Tomaiuolo, Stefano Lasaponara

**Affiliations:** 1 Dipartimento di Psicologia 39, Sapienza Università di Roma, 00185 Roma, Italy; 2 Laboratorio di Neuropsicologia dell’Attenzione, Fondazione Santa Lucia IRCCS, Neurorehabilitation Hospital, 00179 Roma, Italy; 3 Fondazione Patrizio Paoletti—06081 Assisi, Perugia, Italy; 4 Area of Neuroscience, SISSA, 34136 Trieste, Italy; 5 Auxilium Vitae—Neurorehabilitation Hospital, 56048 Volterra (Pisa), Italy; 6 Dipartimento di Medicina Clinica e Sperimentale, Università degli Studi di Messina, 98122 Messina, Italy; 7 Dipartimento di Scienze Umane, Libera Università Maria Santissima Assunta—LUMSA, 00193 Roma, Italy

**Keywords:** spatial neglect, right brain damage, predictive coding, MMN, P3 wave

## Abstract

Right brain-damaged patients with unilateral spatial neglect fail to explore the left side of space. Recent EEG and clinical evidence suggests that neglect patients might suffer deficits in predictive coding, i.e. in identifying and exploiting probabilistic associations among sensory stimuli in the environment. To gain direct insights on this issue, we focussed on the hierarchical components of predictive coding. We recorded EEG responses evoked by central, left-side or right-side tones that were presented at the end of sequences of four central tones. Left-side and right-side deviant tones produce a pre-attentive Mismatch Negativity that reflects a lower-order prediction error for the ‘Local’ deviation of the tone at the end of the sequence. Higher-order prediction errors for the frequency of these deviations in the acoustic environment, i.e. ‘Global’ deviation, are marked by the P3 response. We show that when neglect patients are immersed in an acoustic environment characterized by frequent left-side deviant tones, they display no pre-attentive Mismatch Negativity both for left-side deviant tones and infrequent omissions of the last tone, while they have Mismatch Negativity for infrequent right-side deviant tones. In the same condition, neglect patients show no P300 response to ‘Global’ prediction errors for deviant tones, including those in the non-neglected right-side, and omissions. In contrast to this, when right-side deviant tones are predominant in the acoustic environment, neglect patients have pre-attentive Mismatch Negativity both for right-side deviant tones and infrequent omissions, while they display no Mismatch Negativity for infrequent left-side deviant tones. Most importantly, in the same condition neglect patients show enhanced P300 response to infrequent left-side deviant tones, notwithstanding that these tones evoked no pre-attentive Mismatch Negativity. This latter finding indicates that ‘Global’ predictions are independent of ‘Local’ error signals provided by the Mismatch Negativity. These results qualify deficits of predictive coding in the spatial neglect syndrome and show that neglect patients base their predictive behaviour only on statistical regularities that are related to the frequent occurrence of sensory events on the right side of space.

## Teaser

When patients with left spatial neglect are in an acoustic environment with frequent left-side tones, they are unable to catch and exploit statistical regularities in the omissions of tones and in the occurrence of tones in the right side of space. This deficit affects both pre-attentive processing and contextual updating.

## Graphical Abstract

**Graphical Abstract:**
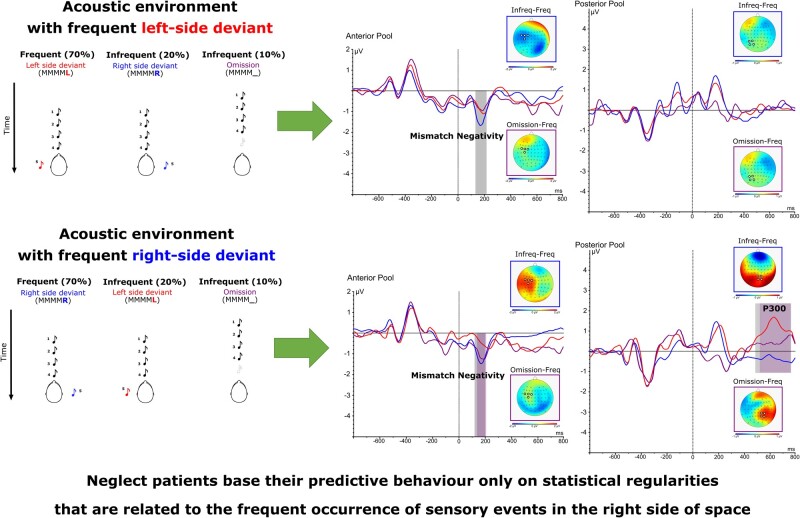

## Introduction

Attending relevant sensory events is facilitated if one can pick-up and exploit the spatial and temporal contingencies in the environment. According to the ‘Predictive Coding Hypothesis’,^1^ the cortex’s cytoarchitecture implements a multi-level hierarchical top-down prediction algorithm that helps to anticipate incoming sensory stimuli. Each hierarchical processing level stores an internal model of the environment, which is generated basing on statistical regularities that have governed past inputs. This model creates top-down predictions that regulate lower processing levels. Predictions are compared continuously with novel incoming inputs and when a mismatch is detected between predicted and actual events, a ‘prediction error’ is transmitted from lower hierarchical levels to higher ones to adjust the internal model. Here, by ‘predictive coding’ we shall refer more broadly to ‘predictive processing’, which encompasses both computational models based upon discrete partially observed Markov decision processes^2^ and Kalman filter style models.^3^

In the human, right brain damage (RBD) often produces left spatial neglect, which consists of the inability to attend the contralesional side of sensory, body and imagery space^4–8^ and implies poor recovery outcome after stroke.^9^^,^^10^ Neglect most frequently follows lesions of parietal-frontal white matter,^5–11^^,15^ though posterior lesions producing both left hemianopia and a concomitant splenial disconnection^16–18^ or lesions in the thalamus or the basal ganglia can also cause neglect.^19–23^

Although deficits of predictive coding in patients with neglect have never been directly investigated, a few studies offer insights on this issue. To evaluate pre-attentive processing in patients with neglect, in a pioneering study, Deouell et al.^24^ investigated the EEG responses evoked by infrequent final tones that deviated 30° medial to the position of a previous series of standard tones that were presented 60° to the left or the right of the patient’s mid-sagittal plane. In the healthy brain, these ‘deviants’ typically elicit a Mismatch Negativity (MMN) which is recorded over anterior-central derivations 130–150 ms after the presentation of the tone. The MMN is now considered a pre-attentive EEG marker of the prediction-error triggered by the detection of deviant tones.^25^ Deouell et al.^24^ found that patients with neglect have a reduced MMN for deviant tones on the left side of space, thus demonstrating a pre-attentive deficit and that the predictive coding of the same stimuli can be compromised in neglect.

In a recent study, we have explored the EEG correlates of attentional orienting in patients with neglect.^26^^,^^27^ We have found that these patients show a pathological drop in the amplitude of the P3a response evoked by targets that, following a right-pointing cue, were infrequently presented in the left side of space, i.e. invalid targets. Vice-versa, invalid targets in the attended right side of space produced a pathological enhancement of the P3a. Since the P3a is evoked by infrequent novel stimuli,^28^ we concluded that neglect patients have reduced novelty reaction to stimuli in the contralesional space and enhanced novelty reaction to stimuli in the ipsilesional space. Besides, patients with neglect displayed a generalized reduction of the P3b component evoked by all types of targets on the left side of space. Since the P3b probably marks the processing of the match or mismatch between expected and actual stimuli (i.e. probabilistic contextual updating^28^), this latter result suggests that neglect patients are unable to monitor the probabilistic association between sensory cues that guide spatial attention and targets in the contralesional space.

Using the oddball-MMN paradigm and dynamic causal modelling of EEG responses, Dietz et al.^29^ (see also Dietz et al.^30^ for a study in healthy participants) recently found that patients with left spatial neglect have functional disconnection in the right hemisphere in response to left-deviants and intact connectivity in the left hemisphere in response to right deviants. In addition, neglect patients showed left hemisphere fronto-temporal connectivity to deviants in the neglected left side, which might be a compensatory mechanism. In the same patients, reduced parietal-frontal connectivity in the left hemisphere was correlated to higher neglect severity. The authors hypothesized that altered connectivity in neglect might entail defective sampling of evidence in the left space and a consequent defective formulation of probabilistic belief for events in the same space.

Taken together, these few studies suggest that patients with neglect might suffer predictive coding deficits both at an early pre-attentive level and at a later processing level that helps to update the representation of probabilistic contingencies in the environment. Bekinschtein et al.^31^ have devised a behavioural paradigm that can help to test and eventually dissociate pre-attentive and contextual updating predictive deficits. In this task, two levels of auditory predictions are specified. The first lower-order level is defined as ‘Local’ and has to do with the perception of regularities/irregularities in a sequence of five tones. Regular-standard sequences are made of five identical tones, e.g. AAAAA, while in irregular-deviant sequences, the last tone is different from the four previous ones, e.g. AAAAB, or is missing, e.g. AAAA_. Deviants and omitted tones evoke a MMN. The second and higher-order level of prediction is defined as ‘Global’ and has to do with the capability of recognizing that in a given block of trials regular sequences are more frequent than irregular ones, or vice versa, and elaborating corresponding sensory expectations. At the electrophysiological level, the occurrence of an infrequent sequence triggers a P3 response.

Here, using a variant of the paradigm devised by Bekinschtein et al.,^31^ we have manipulated both the ‘Local’ and ‘Global’ spatial-auditory features of the environment to test pre-attentive (MMN) and contextual-updating (P3) deficits in patients with left spatial neglect. To this aim, we investigated the brain electrophysiological responses that are evoked by ‘Local’ and ‘Global’ auditory irregularities, with specific reference to irregularities produced by the infrequent displacement of tones in the left or right side of space. In different blocks of trials, left-side displacements were frequent and right-side displacements infrequent or vice versa. This allowed investigating whether patients with neglect can develop spatial predictive coding based on the more or less frequent occurrence of events in each lateral space. As an example, one might hypothesize that when deviations are more frequent in the neglected left side of space, patients with neglect can still infer this probabilistic distribution from the infrequent occurrence of events in the attended right side, and accommodate predictive coding for the two sides of space or, eventually, only for the right-side. Alternatively, one might hypothesize that in these patients, predictive coding is exclusively based on the predominant occurrence of events in one of the two lateral sides of space. In this case, due to their pathological bias of spatial attention, patients with neglect could generate spatial predictions only basing on the more frequent occurrence of events in the right side of space though not when events are more frequent in the left side of space. Recording of the MMN and P3 EEG markers of predictive coding helps in contrasting and verifying these different scenarios.

## Material and methods

### Participants

Patients were examined at the rehabilitation hospital Fondazione Santa Lucia IRCCS (Rome). Patients with bilateral strokes, signs of dementia or history of previous neurological illness were excluded. Two groups of patients completed the experimental protocol: thirteen right-brain-damaged patients with left spatial neglect (N+) and fourteen right-brain-damaged patients without neglect (N−). In addition, sixteen age-matched healthy participants were tested as controls (HC). Patients and participants were all right-handed and had normal or corrected-to-normal visual acuity. At the time of clinical and experimental examination, all patients were free from confusion and temporal or spatial disorientation. Visual fields were tested with standard kinetic Goldmann perimetry. All patients had intact visual fields. N+ and N− patients did not differ in time elapsed from stroke onset [*F*(1,25) = 3.2, *P* = 0.1; mean = 47 days]. Age was equivalent among N+, N− and HC [*F*(2,40) = 2.4, *P* = 0.6; mean age: HC = 58.3; N+ = 67.6; N− = 59.2 years]. Unilateral neglect was assessed with a battery of six standardized tests (for details, see Supplementary material): (i) Line bisection^32^^,^^33^; (ii) Letter cancellation^34^; (iii) Line cancellation^35^; (iv) Star cancellation^36^; (v) Sentence reading test^32^; and (vi) Wundt–Jastrow area illusion test.^37^ Patients who failed on at least two out of the six tests were classified as suffering left spatial neglect. Auditory neglect was separately assessed through a ‘straight-ahead’ acoustic task.^38^ This task requires to judge the alignment of unseen acoustic stimuli, moving along a 180° arc, to the subjective head-body mid-sagittal plane (for details, see Supplementary materials). Clinical and demographic data are reported in Table 1. Patients and controls gave their informed consent for participating in the study that was approved by the Institutional Ethical Committee of the Fondazione Santa Lucia IRCCS.

**Table 1:** Clinical and demographic group data of RBD patients with left spatial neglect (N+), without left spatial neglect (N−) and healthy controls (HCs).

Patients Group	Sex		Age (y)	Stroke onset (days)	Line bisection (200 mm) rightward deviation (mm)	Letter cancellation		Line cancellation		Star cancellation		Sentence reading test	Wundt-Jastrow illusion (unexpected responses)		Deviation of the subjective acoustic ‘straight ahead’
						Left	Right	Left	Right	Left	Right		Left	Right	
N−; *n* = 14															
	M = 11	Mean	59.2	40.1	0.01	52(53)	50.4(51)	10.9(11)	9.9(10)	26.3(27)	26.8(27)	5.6(6)	1.1(20)	0(20)	1.2°
	F = 3	S.D.	15.4	15.8	5.2	1.1	0.9	0.2	0.2	1.3	0.6	0.9	2.7	0	3.3°
N+; *n* = 13															
	M = 6	Mean	67.6	56	19.1	29(53)	43.3(51)	8.2(11)	9.2(10)	9(27)	21.8(27)	3.5(6)	12.4(20)	1.8(20)	17.4°
	F = 7	S.D.	11.1	27.6	20.2	17.2	14	3.4	2.1	8.8	7	2.5	6.7	2.3	20.6°
HC; *n* = 16															
	M = 7	Mean	58.3												−0.9°
	F = 9	S.D.	11.1												1.7°

Maximal scores for each test are reported in parenthesis. In the acoustic task, positive scores indicate rightward deviation of the subjective auditory ‘straight-ahead’ and negative scores leftward deviation.

### Lesion mapping

Individual 1.5 T MRI scans were corrected for inter-individual differences in brain size and brain volume orientation, using a transformation into the standardized MNI space using the software REGISTER (http://www.bic.mni.mcgill.ca/ServicesSoftwareVisualization/Register Accessed 31 May 2021). This program allows performing manual normalization basing on twelve pre-defined anatomical landmarks. Damaged areas were mapped through the DISPLAY software (http://www.bic.mni.mcgill.ca/software/Display/Display.html Accessed 31 May 2021). An expert anatomist (Prof. F. Tomaiuolo), who was blind to the purpose of the study and the identity of patients, run normalization and lesion mapping. In each experimental group, the MNI coordinates of the centroids of maximal lesion overlap areas were defined using the command DISPLAY. To check whether peaks of lesion overlap encroached upon white matter pathways, we used the DSI Studio software (http://dsi-studio.labsolver.org Accessed 31 May 2021) to superimpose lesion peaks on the three-dimensional reconstruction of white matter pathways taken from the diffusion tensor atlas by Yeh et al.^39^ See Supplementary material for details on lesion mapping procedures.

### Procedure and stimuli

Participants were tested with the head comfortably supported by a chin rest, in a dimly lit, sound attenuated and electrically shielded room. Auditory stimuli were presented using in-ear headphones. Participants were only asked to listen to these sounds and keep their gaze stable on a central fixation-cross that was presented on a video monitor (22 inches) at a viewing distance of 57.5 cm. The presentation of auditory stimuli was performed with E-prime software.^40^

The task consisted of an auditory oddball-paradigm in which the location of a sound shifts unpredictably from a central location, aligned to the body-midsagittal plane, to the left or the right side of egocentric space. We used an inter-aural time delay of 800 μs^29^ between left and right ears together with a change in sound-pressure in the ipsilateral (i.e. 70 db) and contralateral (i.e. 22 db) ear to induce subjective leftward or rightward ∼90° shift in the sound location in the horizontal plane. All other parameters of the sound were kept constant (duration: 50 ms, 440 Hz frequency, no harmonics, 5 ms of fade-in and fade-out).

**Figure 1:**
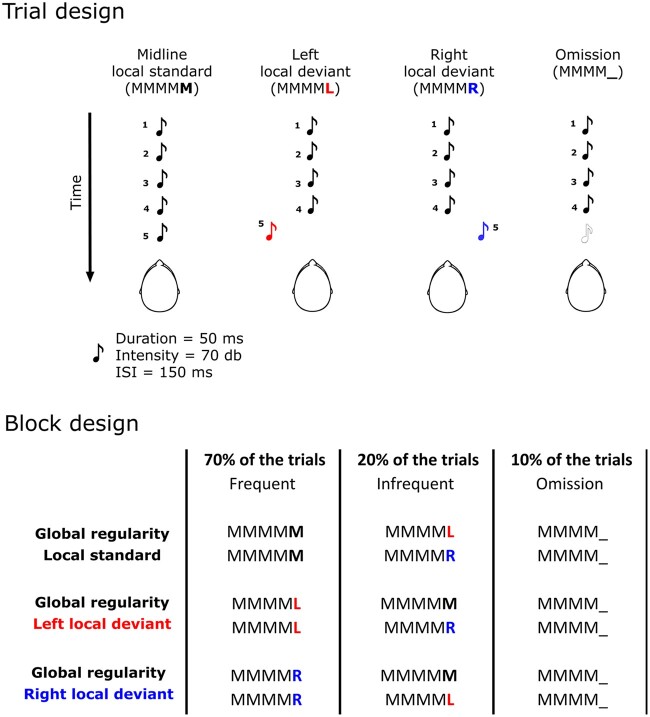
**Task structure:** structure of the four types of auditory trials and the six experimental blocks/experimental-conditions used in the study.

Each trial lasted 850 ms and included a series of 5 tones with an inter-stimulus interval of 150 ms. Four different types of trials were presented: (i) five identical tones presented at the central midline (*local standard*, denoted MMMM**M**); (ii) four identical tones at midline followed by a deviant tone in the left space (**L**) (*left local deviant*, denoted MMMM**L**); (iii) four identical tones at midline followed by a deviant tone in the right space (**R**) (*right local deviant*, denoted MMMM**R**); and (iv) four identical tones with no fifth tone (*Omission*: MMMM**_**); These trial types were organized and presented within six different semi-randomised experimental blocks of ∼4-min duration, with variable inter-trial intervals (700–1000 ms), each relating to a different variation of the ‘Global’ predictive characteristics of the stimuli. Similar to Bekinschtein et al.,^31^ each block of trials started with the repetition of 20 identical trials, i.e. *frequent* series (MMMM**M** *local standard* or *left* and *right local deviant* MMMM**L**/MMMM**R** in different blocks), to establish the ‘Global’ regularity. These were followed by 100 trials with 70% of *frequent* series, 20% *infrequent* series (MMMM**L** or MMMM**R** when the *frequent* series is MMMM**M** and vice versa), and 10% of the *Omission* series. The structure of the different block of trials is depicted in Fig. 1 (see Supplementary material for details). The whole experiment included two experimental sessions (6 blocks of trials in each session for 1440 trials) that were run in different days and separated by a one-two day interval. All experimental blocks were randomized and counterbalanced across sessions and participants.

In an initial screening test, maintenance of primary acoustic sensory processing of left-side and right-side deviant tones, was verified by recording ERPs in blocks of single left-deviants and right-deviant tones (see Supplementary material).

### EEG recording and pre-processing

The EEG was recorded using a Brain Vision system from 64 electrodes placed according to the 10–10 system montage. All scalp channels were online referenced to Fcz. Horizontal eye movements were monitored with a bipolar recording from electrodes at the left and right outer canthi. Blinks and vertical eye movements were recorded with an electrode below the left eye, which was referenced to site Fp1. The EEG from each electrode site was digitized at 250 Hz with an amplifier bandpass of 0.01–60 Hz, including a 50 Hz notch filter, and was stored for off-line averaging. Continuous EEG was recalculated against the average reference, low-pass filtered (30 Hz cut-off) and successively segmented in epochs lasting 1600 ms, locked to the first tone of the trial sequence. A time period of 200 ms before this event was used for baseline-correction. Before automatic artefact rejection, the ocular correction was performed accordingly to Gratton & Coles algorithm.^41^ Artefact rejection was performed before signal averaging to discard epochs in which deviations in eye position, blinks, or amplifier blocking occurred. All epochs in which EOG amplitudes and EEG amplitudes were greater than ±60 mV were excluded from further analysis. On average, 4%, 6% and 7% of the trials were rejected for violating these artefact criteria in HC, N− and N+ group, respectively.

### Statistical analyses

#### Clinical and demographical data

We compared the clinical performance between the two groups of patients by entering the individual score of Line bisection, and Sentence reading test in a series of unpaired two-tailed *t*-test with a *P*-level set to 0.05. Performances in the Letter cancellation, Line cancellation, Star Cancellation and the Wundt-Jastrow Area Illusion test were compared through a series Group (N−, N−) × Target Side (Left, Right) repeated-measures ANOVA. Performances in the ‘straight-ahead’ acoustic task were compared through a one-way between groups (HC, N−, N−) ANOVA.

#### Lesion analyses

**Figure 2:**
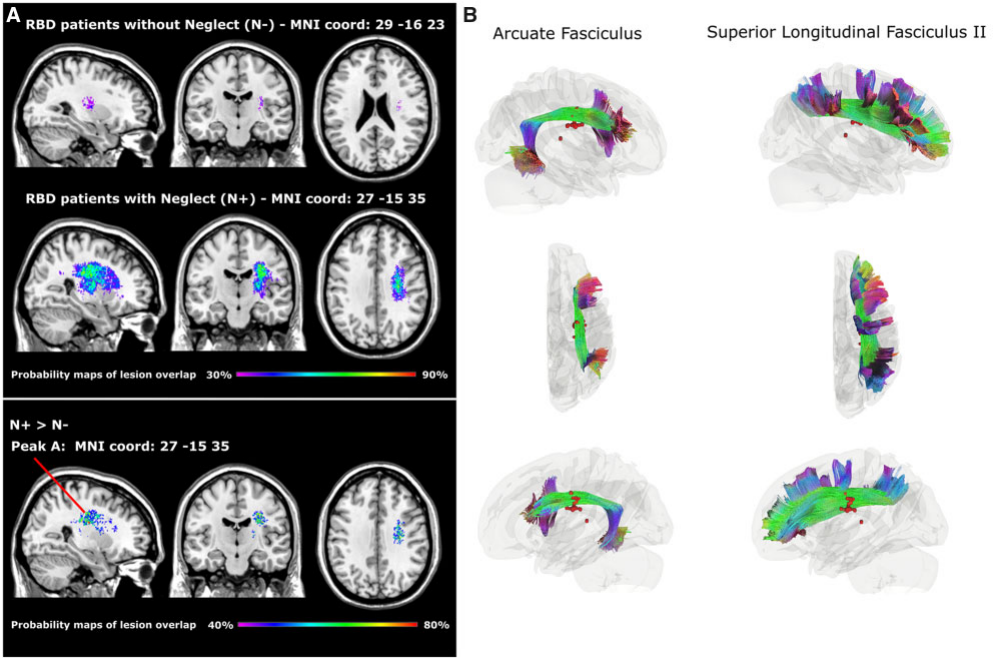
**Lesion probability maps and tridimensional reconstruction of white matter fibres:** (**A**) Probability maps of lesion overlap: First row, patients without neglect (N−); second row, patients with neglect (N+). The third row represents the peaks of lesion overlap resulting from the N+ minus N− subtraction. Note, the main peak of the subtraction (MNI coord: 27, −15, 35) is located in the superior longitudinal and arcuate fasciculi. (**B**) Tridimensional reconstruction of the superior longitudinal and arcuate fasciculi, according to the atlas by Yeh et al. (2018). Red circles represent the highest (70–75%) peaks of lesion overlap resulting from the N+ minus N− subtraction.

Descriptive statistical comparisons of lesion mapping were run by subtracting the probability map of the N− group from that of the N+ group and by comparing, with Fisher exact test, the frequency of damage occurrence at the centroids of the areas of maximal lesion overlap. Lesion probability maps resulting from this subtraction and the corresponding MNI coordinates of centroids of lesion overlaps are reported in Fig. 2 and in Supplementary Fig. 2.

#### ERPs analyses

##### ‘Local effect’

To check for the electrophysiological correlates of ‘Local’ deviance, we averaged together, and independently from their global probability, all trials in which the last tone deviated from the midline (*local deviant*). Separate averages were calculated for left and right deviants. Similarly, we averaged all *local standard* trials^31^ (see Fig. 1). Then, using Brainstorm^42^ (http://neuroimage.usc.edu/brainstorm Accessed 31 May 2021) we run a nonparametric FDR-corrected permutation analysis with Monte Carlo method (see Supplementary material for details) contrasting, in each group, *local deviant* versus *local standard* experimental condition separately for left and right deviants. In all groups, this analysis pointed out an effect of condition (*P* < 0.05) corresponding to a negative cluster in the observed data around 130–200 ms (i.e. MMN) and a positive cluster around 230–350 ms (i.e. P3a; see Supplementary Table 1). In a second step, to check for the presence of differences in these ERPs components between groups, we entered individual subtractive mean amplitude of MMN and P3a from period of interests, in a series of Group (HC, N− and N+) × Side of deviance (Left, Right) repeated-measures ANOVA. *Posthoc* tests were corrected for multiple comparisons using Bonferroni correction.

##### ‘Global effect’

‘Global’ effects were measured by averaging, independently from local deviance, all *frequent* (i.e. 70% probability) and all *infrequent* (i.e. 20% probability) trials separately for the left and right side of space. Also in this case, we run a non-parametric FDR-corrected permutation analysis contrasting in each group, *global infrequent* versus *global frequent* experimental conditions separately for left and right side of space. With the only exception of the T-contrast within N+ group for the left side of space, in all cases, this analysis indicated an effect of condition (*P* < 0.05) corresponding to a positive cluster (i.e. P3b) in the observed data in a time window ranging from 450 to 750 ms after the onset of the last tone of the sequence (see Supplementary Table 1). To check for between-groups differences in P3b amplitude, individual differential data were entered in a Group (HC, N− and N+) × Side of global infrequency (Left, Right) repeated-measures ANOVA. *Posthoc* tests were corrected for multiple comparisons using Bonferroni correction.

##### Lateralized ‘Local’ and ‘Global’ effects

In a final series of nonparametric FDR-corrected permutation analyses, we checked within each group, the ‘Local’ and ‘Global’ effects that were produced by a 180° deviation of the last tone in auditory space. To this aim, we compared: (i) EEG activity evoked by *infrequent left local deviant* trials (MMMM**L**) with that evoked by *frequent right local deviant* ones (MMMM**R**) and vice versa and (ii) EEG activity evoked by *omissions* with that elicited by frequent *left* and *right local deviant* trials. For each group, significant positive and negative clusters with relative time windows suggesting the presence of an MMN and a P3b related EEG activity, are reported in Supplementary Table 1. Successively, individual mean amplitudes of these ERPs components were entered in a Group (HC, N− and N+) × Side of local deviance (Left, Right) × Frequency (Freq, Infreq, Omission) repeated-measures ANOVA to check for between-group differences. *Posthoc* tests were corrected for multiple comparisons using Bonferroni correction.

### Data availability

The data that support the findings of this study are available on request from the corresponding authors. The data are not publicly available, as they include information that could compromise the privacy of the research participants.

## Results

### Clinical results

A series of between-group comparisons, showed that compared to N−, N+ patients had significant rightward spatial biases in all neglect tasks (see Table 1). N+ had a higher rightward bias in line bisection [*t*_(25)_ = −3.4, *P* = 0.002, unpaired *t*-test] and showed a higher number of left side omissions in the Sentence reading task [*t*_(25)_ = 2.9, *P* = 0.007, unpaired *t*-test]. In the Letter cancellation [*F*(1,25) = 13.4, *P* = 0.001, $\eta_{p}^{2}$= 0.34], Line cancellation [*F*(1,25) = 12.9, *P* = 0.001, $\eta_{p}^{2}$= 0.34], Star Cancellation [*F*(1,25) = 34.4, *P* = 0.0000, $\eta_{p}^{2}$= 0.57] and in the Wundt-Jastrow Area Illusion task [*F*(1,25) = 20.9, *P* = 0.0001, $\eta_{p}^{2}$= 0.45], N+’s performance differed from that of N− more for stimuli positioned in the left side of space than for stimuli placed in the right side of space, as indexed by significant Group × Side interactions.

N+ patients showed a significant rightward deviation of the subjective acoustic ‘straight-ahead’ both as compared to HC and N− [*F*(2,40) = 12.4, *P*  < 0.001; HC: −0.9°; N−: +1.2° and N+: +17.4°]. No difference was present between HC and N− (*P* > 0.05).

### Anatomical results

N+ group had larger lesion than N− [36813 vs. 11646; *F*(1,25) = 7.3; *P* = 0.01]. The lesion probability maps resulting from the subtractions between N+ and N− showed one cluster ranging from 40% to 80% of lesion overlap in N+ and no corresponding overlap, i.e. 0%, in N− patients (Fischer exact test, *P* = 0.006). The maximal lesion overlap (i.e. 75%) was located in the white matter underneath the inferior parietal-frontal cortex in the region of the superior longitudinal and arcuate fasciculi (see Fig. 2: Peak A, MNI coordinates: 27, −15, 35; see Supplementary Fig. 2 for the complete range of overlap). This finding confirms the role of parietal-frontal white matter disconnection in spatial neglect.^11–14^

### Electrophysiological results

#### ‘Local’ effect—MMN component

**Figure 3:**
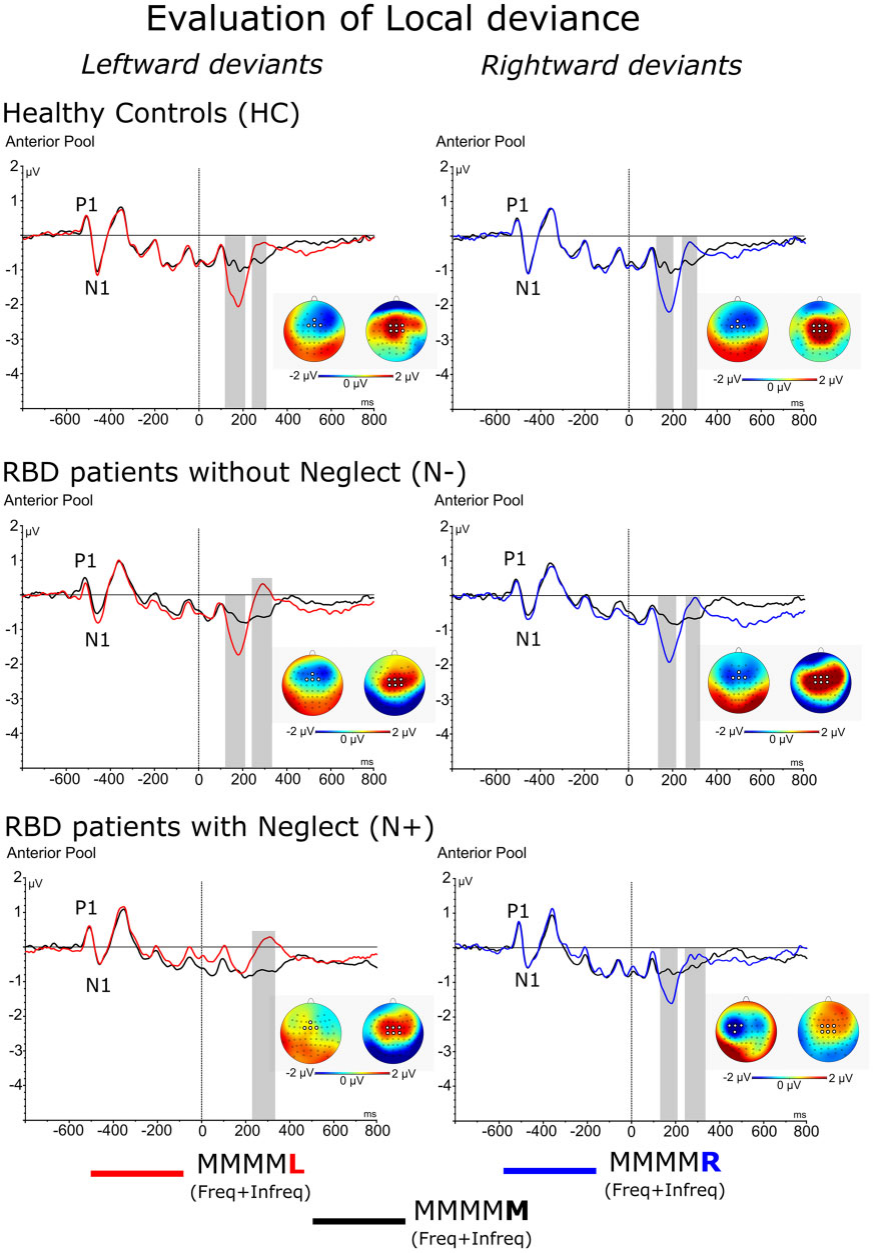
**ERPs components related to local effects:** Waveforms and relative differential scalp topographies of the MMN evoked by the *left* and *right local deviants* as compared to *local standard* in HC, N− and N+. Grey shades indicate post-stimulus onset time-intervals in which a significant statistical difference is present.

A main effect of Group [*F*(2,40) = 6.0, *P* = 0.005, $\eta_{p}^{2}$= 0.23] showed that the amplitude of MMN was generally reduced in N+ (−0.07 μV) with respect to HC (−0.79 μV; *P* < 0.01) and N− (−0.84 μV; *P* < 0.01) while no difference was present between the latter two groups. A significant Group × Side of deviance interaction [*F*(2,40) = 5.7, *P* = 0.006, $\eta_{p}^{2}$= 0.22] showed that in N+ the MMN was suppressed in response to *left-side deviant* stimuli (HC = −0.75 μV; N− = −0.82 μV; N+ = 0.21 μV; both *P* < 0.01) while the amplitude of MMN evoked by *right-side deviants* was comparable to that of N− and HC (HC = −0.84 μV; N− = −0.86 μV; N+ = −0.36 μV; all *P* = n.s.; see Fig. 3).

**Figure 4:**
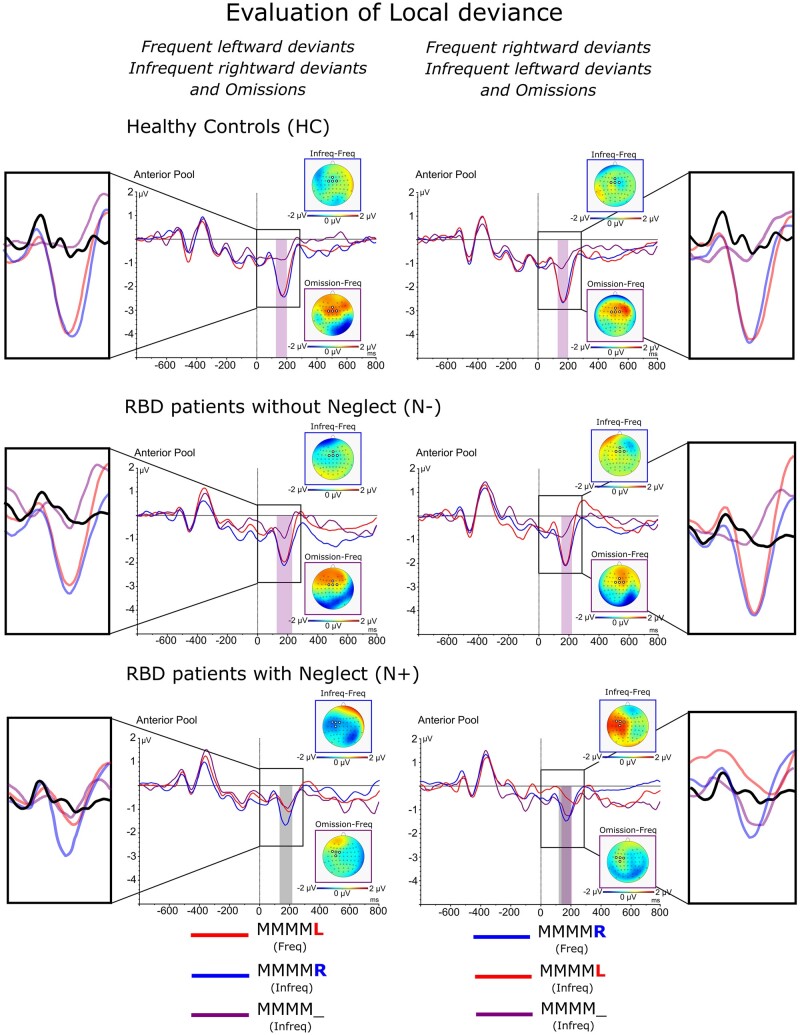
**ERPs components related to lateralized local effects:** Waveforms and relative differential scalp topographies of the MMN component evoked by *frequent* and *infrequent local left*- or *right-deviant* sequences and *omissions* in HC, N− and N+. Grey shades indicate post-stimulus onset time-intervals in which a significant statistical difference is present between *frequent* and *infrequent* trial types. Purple shades indicate post-stimulus onset time-intervals in which a significant statistical difference is present between *frequent* and *omission* trial types. Zoomed squares show the comparison with *local standard* (MMMM**M**) sequences.

In the case of MMN evoked by stimuli that deviated 180° degree in auditory space, a significant Group × Side × Frequency interaction [*F*(2,40) = 3.6, *P* = 0.008, $\eta_{p}^{2}$= 0.15] showed that independently of stimulus frequency (i.e. 70% vs. 20% of trials), in N+, a significant MMN was only found in response to *right-side deviants* (*frequent* MMMM**L** = −0.53 μV vs. *infrequent* MMMM**R** = −1.19 μV, *P* = 0.01; *frequent* MMMM**R** = −0.94 μV vs. *infrequent* MMMM**L** = −0.13 μV, *P* = 0.001; see Fig. 4). This was not found in HC and N−, who displayed comparable MMN for *left-side* and *right-side deviant*, both for *frequent* and *infrequent* stimuli (all *P* = n.s.; see Fig. 4). The same interaction showed that during blocks of trials with *frequent right-side deviants,* in N+ patients *omissions* produced an MMN that was comparable to that evoked by the *right-side deviant*s (*frequent* MMMM**R** = −0.94 μV vs *omissions* = −0.85 μV, *P* = n.s.; see Fig. 4). In all other cases, *omissions* produced no significant MMN.

#### ‘Local’ effect—P3a component

The Group (HC, N− and N+) × Side of deviance (Left, Right) repeated-measures ANOVA did not show any significant main effect or interaction (all *F* < 0.45, all *P* > 0.50), suggesting the presence, in all groups, of a comparable P3a in response to left and right deviant tones.

#### ‘Global’ effect—P3b component

**Figure 5:**
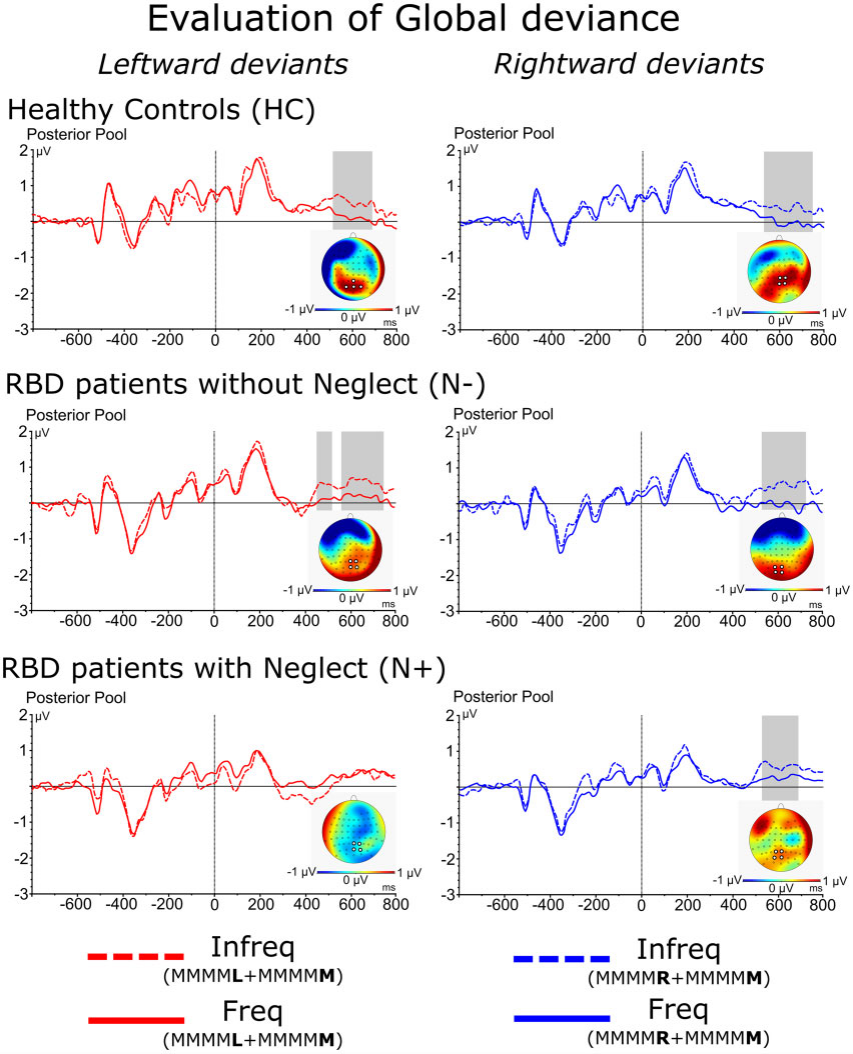
**ERPs components related to global effects:** Waveforms and relative differential scalp topographies of the P3b component evoked by all *frequent* and all *infrequent* stimuli presented in the left and in the right side of space in HC, N− and N+. Grey shades indicate post-stimulus onset time-intervals in which a significant statistical difference is present.

A significant Group × Side of global infrequency interaction [*F*(2,40) = 3.8, *P* = 0.02, $\eta_{p}^{2}$= 0.16] showed that, in N+ the P3b was selectively suppressed in response to *infrequent* stimuli presented in the left side of space (HC = 0.48 μV; N− = 0.42 μV; N+ = 0.01 μV; both *P* < 0.005), while equivalent right-side stimuli evoked a P3b that was comparable to that of HC and N− (HC = 0.45 μV; N− = 0.47 μV; N+ = 0.32 μV; all *P* = n.s. see Fig. 5).

**Figure 6:**
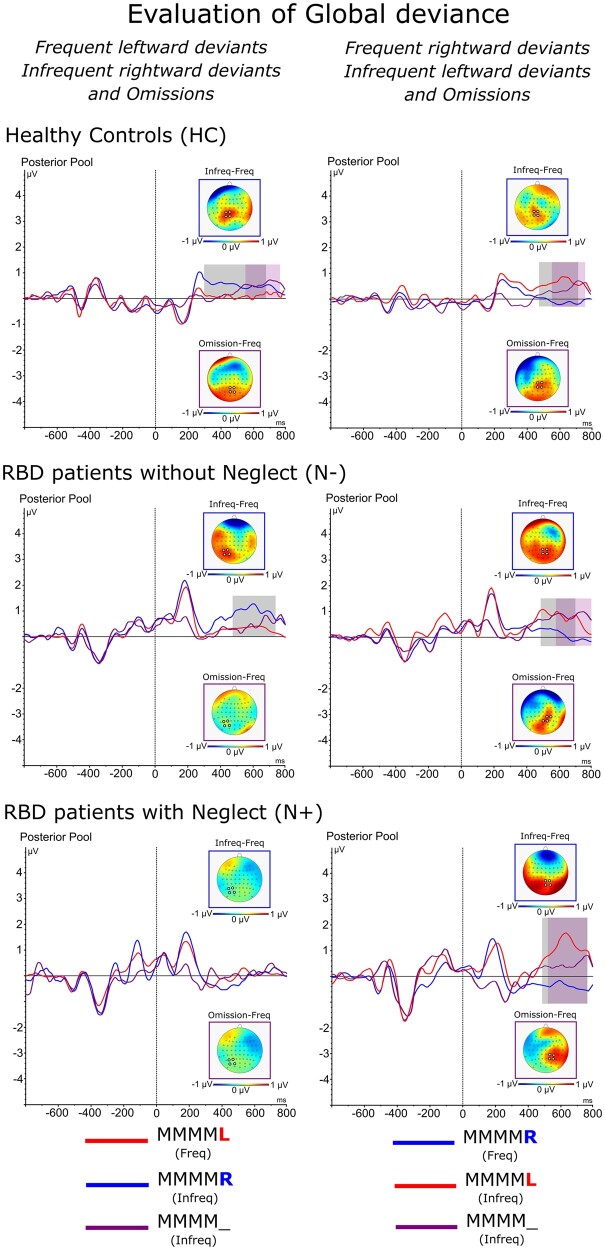
ERPs components related to lateralized global effects: Waveforms and relative differential scalp topographies of the P3b component evoked in response to the *frequent* and *infrequent local left-* or *right-deviant* sequences and *omissions* in HC, N− and N+ during blocks with predominant MMMM**L** or MMMM**R**. Grey shades indicate post-stimulus onset time-intervals in which a significant statistical difference is present between *frequent* and *infrequent* trial types. Purple shades indicate post-stimulus onset time-intervals in which a significant statistical difference is present between *frequent* and *omission* trial types.

The analysis of ‘Global’ effects in blocks of trials with final tones that deviated of 180° degrees in the acoustic space showed that [Group × Side of global deviance × Frequency interaction: *F*(2,80) = 10.2, *P *=* *0.001, $\eta_{p}^{2}$= 0.28] in blocks with *frequent left-side deviants*, *infrequent right-side deviants* produced a significant P3b in HC (*frequent* MMMM**L** = 0.13 μV vs. *infrequent* MMMM**R** = 0.69 μV, *P* = 0.001) and N− (*frequent* MMMM**L** = 0.27 μV vs. *infrequent* MMMM**R** = 0.92 μV, *P* = 0.001) though not in the N+ (*frequent* MMMM**L** = 0.12 μV vs. *infrequent* MMMM**R** = 0.13 μV, *P* = n.s.; see Fig. 6). In contrast, in blocks with *frequent right-side deviant*s, both *infrequent left-side deviants* and *omissions* produced a significant P3b in all groups (HC: *frequent* MMMM**R** = 0.09 μV vs. *infrequent* MMMM**L** = 0.68 μV, *P* = 0.001; N−: *frequent* MMMM**R** = 0.11 μV vs. *infrequent* MMMM**L** = 0.79 μV, *P* = 0.001; N+: *frequent* MMMM**R** = −0.2 μV vs. *infrequent* MMMM**L** = 1.4 μV, *P* = 0.001; see Fig. 6).

In HC, *omissions* produced a significant P3b both when compared to *frequent left-side and frequent right-side deviant*s (*frequent* MMMM**L** = 0.13 μV vs. *omission* = 0.64 μV, *P* = 0.001; *frequent* MMMM**R** = 0.09 μV vs. *omission* = 0.53 μV, *P* = 0.001). It is interesting to note that, in these cases, the P3b component produced by *omissions* was delayed by about 200 ms with respect to that evoked by *infrequent deviants*. In contrast, in N− and N+ *omissions* produced a significant P3b only in blocks with *frequent right-side deviants* (N−: *frequent* MMMM**R** = 0.11 μV vs. *omission* = 0.78 μV, *P* = 0.001; N−: *frequent* MMMM**R** = −0.2 μV vs. *omission* = 0.58 μV, *P* = 0.001). Notably, in N+ the P3b evoked by *omissions* was smaller than that evoked by *infrequent left-side deviant* stimuli (*omission* = 0.58 μV, *P* = 0.001 vs. *infrequent* MMMM**L** = 1.4 μV, *P* = 0.001; see Fig. 6).

## Discussion

To gain novel insights into the spatial neglect syndrome, we investigated the ability of neglect patients to build-up and exploit predictions on the probability that an acoustic stimulus will occur in the neglected left or in the attended right side of space. To this aim, we used a variant of an auditory task^31^ that allows studying the electrophysiological correlates of predictive coding at different processing levels.

Two preliminary findings are relevant. First, compared to controls patients with neglect showed intact primary sensory processing of both left-side and right-side deviant tones, as demonstrated by the comparable latency and amplitude of the P1 and N1 responses evoked by these tones (see screening test in Supplementary Results). Second, in neglect patients, left-side deviants evoked an MMN that was smaller in amplitude than that evoked by right-deviants. This result replicates the pioneering observations by Deouell et al.^24^

As for the specific aims of our study, we found that in patients with neglect, the reduction in amplitude of the MMN evoked by left-side deviant tones matched an interesting modulation of the MMN evoked by omissions of tones. When in a block of trials, left-side deviants represented the most frequent event, the omission of the last tone evoked a reduced MMN that was equivalent in amplitude to that evoked by left-deviants. In contrast, when right-deviant tones were the most frequent event in a block of trials, the amplitude of the MMN evoked by omitted tones increased and reached the amplitude of the MMN evoked right-deviants. These results show that the pre-attentive processing of the same sensory omissions changed utterly depending on the predominant presentation of tones in the left or right side of space, thus exemplifying the profound effects that the probabilistic sensory context can exert on the behaviour of neglect patients.^25^

Most interestingly, HCs and patients without neglect showed no MMN in response to omissions (see Fig. 4). MMN to omissions is a classical finding in paradigms where tone-pitch is the salient stimulus feature in the task (for review^43^) In these paradigms, MMN to omissions is considered a proof of the predictive, rather than sensory-adaptation, nature of the MMN.^25^ Nonetheless, in the task used in our study the relevant stimulus feature was tone-position and not pitch. To the best of our knowledge, MMN studies run with tone-position paradigms have never included omissions.^44^ Our observations seem therefore to provide a new finding highlighting the role of the task set on the generation of the MMN. Different findings with tone-pitch and tone-position paradigms might imply that while in tone pitch-paradigms a ‘no-tone’ omission is pre-attentively treated as a change in pitch, in tone position-paradigms a ‘no-tone’ omission is not pre-attentively treated as a change in tone position. How to frame within this interpretation the observation that in patients with neglect omissions evoked an MMN when they were infrequently interspersed with frequent sequences ending with a shift of the last tone in the right side of space? One possibility is that the combination of neglect for the left side of space together with the pathological bias of attention towards the right side of space enhanced expecting sensory events in right side so that omissions were perceived as the non-occurrence of a highly expected right-side event. Future studies should address and clarify these points.

The influence that the most frequent event in a block of trials played in the modulation of ERPs in patients with neglect, was even more highlighted by the analyses of the P3 response that marks ‘Global’ predictive coding. In this case, when left-deviant tones were most frequent in a block of trials, no P3 was evoked by infrequent right-deviants and omissions. In striking contrast with these results, when right-deviant tones were most frequent in a block of trials, both infrequent omissions and infrequent left-side deviants enhanced the amplitude of the P3. These findings points-out the influence that ‘Global’ predictive coding exerts on late, attentive responses in patients with neglect. The same results show that in these patients, predictions on the probabilistic spatial distribution of sensory events are only generated when sensory events most frequently occur in the right ipsilesional space. This latter conclusion is based on the finding that when acoustic events were more frequent in the left side of space, no P3 marker of ‘Global’ prediction was elicited by infrequent right-deviant tones in the attended space.

Interestingly, compared to both HCs and patients with neglect, patients without neglect showed a partial disturbance of ‘Global’ predictive coding in the left side of space, because when in a block of trials left-deviant tones were predominant, they displayed a P3 response only to right-deviant tones though not to omitted ones. It is important to consider that this was in contrast to what happened in the same group when right-deviant tones were predominant in a block of trials. In this case, both left-deviant and omitted tones evoked the P3.

In terms of hierarchical predictive coding,^1^ the MMN marks the ‘Local’ short-term deviance of a stimulus in a sequence of stimuli and the corresponding release of a prediction-error signal. These signals eventually feed-up the formulation of more complex ‘Global’ long-term predictions that deal with the occurrence of local deviants across the entire pool of sequences presented in a block of trials. In this latter case, prediction errors are indexed by the P3 component. Within this framework, the findings in neglect patients pointed out a clear double dissociation between ‘Local’ and ‘Global’ predictive coding. First, when left-side deviants were predominant in a block of trials, infrequent right-side deviants elicited a normal local MMN error signal though no corresponding P3 response, i.e. no building-up of ‘Global’ predictions (MMN in Fig. 4, blue line in the bottom-left panel; P3b in Fig. 6, blue line in the bottom-left panel). Second, and most importantly, when rightward deviants were predominant in a block of trials, an opposite dissociation was found. In this case, neglect patients displayed no pre-attentive MMN together with enhanced P3b for infrequent leftward-deviant tones (MMN in Fig. 4, red line in the bottom-right panel; P3b in Fig. 6, red line in the bottom-right panel). This latter dissociation points out that ‘Global’ predictive coding does not necessarily depend on local MMN signals and suggests that ‘Local’ and ‘Global’ predictions are elaborated with a high degree of functional independence. In our study, this was also highlighted by the finding that while in HCs and patients without neglect omissions evoked no MMN, in both of these groups omissions evoked a reliable P3b component (see Supplementary Fig. 4; see Fig. 6 for a more detailed comparison with frequent and infrequent local deviants as a function of experimental group and direction of local deviance).

Past studies in healthy participants have shown that frequent pitch-deviants that evoke no P3 can still trigger an MMN, thus highlighting that lower-level ‘Local’ predictions are free from the influence of higher-level ‘Global’ ones.^25^^,^^31^ The other way round, the same studies showed that infrequent sequences of five identical tones evoke no MNN while they trigger a clear P3b response.^25^ The results of pharmacological, behavioural and brain imaging studies converge on the functional dissociation between the MMN and the P3 response. In humans, serotonin reuptake inhibitors increase the amplitude of the MMN without modifying the P3,^44^ while low doses of ethanol reduce the amplitude of the MMN without affecting the P3b.^45^ Sussman^46^ showed that tone sequences with infrequent pitch deviations elicit both the MMN and P3 when participants are asked to focus on single pitch-deviant tones. In contrast, the P3 is maintained, while the MMN is no longer elicited, when participants are asked to focus on the occurrence of sequences rather than on single pitch deviations. Finally, in a MEG study, King et al.^47^ showed that local acoustic deviants evoke a sequence of short-lived patterns of brain activity early after the end of the tone sequence (120–200 ms after the fifth tone). In comparison, violations of ‘Global’ auditory regularities lead to a later (150–700 ms after the fifth tone), more stable and long-lasting pattern of activity, that engage working memory resources in parietal and prefrontal areas.^47^

In our study, to explore the automatic building up of ‘Local’ and ‘Global’ expectations, we avoided requiring the report of the presence or localization of the last tone in a sequence. Lack of reports limits the possibility of evaluating whether patients, with and without neglect, consciously perceived these tones when no MMN, P3 or both were evoked. It has been proposed,^48^ and it is currently debated,^49^ that the P3 marks the conscious processing of sensory stimuli. In neglect patients, the lack of the P3 in response to all types of tones when leftward deviant tones were predominant in a block of trials, is compatible with the view that these patients have reduced awareness of events in the left side of space. Nonetheless, our study also demonstrates a relevant effect of predictive coding on the P3 evoked by left-side deviant tones in patients with neglect. Indeed, when these stimuli were infrequently alternated with frequent rightward-deviant ones, they produced an enhancement of the P3b despite eliciting no corresponding pre-attentive MMN processing. How to interpret this finding? A plausible hypothesis is that since these tones were released binaurally so that an acoustic input was always presented in the attended right side of space/ear, they were interpreted as a different and novel type of spatially deviant tones. Neglect patients often display alloacusia,^50^^,^^51^ where acoustic stimuli in the left side of space are reported as coming from the ipsilesional right side. Inquiries on the ventriloquist illusion have highlighted that when a concurrent visual stimulus shifts the subjective position of deviant tones to that of standard ones, the MMN is suppressed.^52^ We hypothesize that the lack of pre-attentive MMN processing and the enhanced P3b attentive processing contribute to alloacusia of left-side acoustic stimuli in neglect patients.

Based on neuro-computational simulations of the consequences of brain lesion on explorative ocular behaviour, Parr and Friston^53^^,^^54^ (see also Parr et al.,^55^) have recently advanced a series of hypotheses on predictive coding in patients with neglect. They proposed that due to parietal-frontal white matter disconnection in the right hemisphere, neglect patients would be biased away from the contralesional side of space because they expect low novelty reduction and uncertainty resolution from fixating locations in that side of space. Put in short, the left side of space would offer poor changes in their beliefs on the probabilistic contingencies in that side of space, i.e. poor epistemic affordance. This view is generally compatible with our data: nonetheless, there are two findings that current neuropsychological computational models should try to incorporate. The first is that neglect patients show an exaggerated novelty reaction to right side visual stimuli^26^: this means that the reduced epistemic affordance of the left side of space is matched with a pathologically increased epistemic affordance of the right side. The second is that our study shows that the probabilistic organization of the acoustic environment has a significant impact also on the epistemic affordance offered by stimuli in the attended right side of space. This conclusion is based on the finding that in neglect patients, these stimuli trigger no novelty P3 response when they are infrequently interspersed among frequent left side ones.

To summarize, the present study sheds new light on the poorly explored deficits of predictive coding behaviour in patients with left spatial neglect. The high time-resolution of ERPs allowed us to disclose both the pre-attentive and the attentive/belief updating components of these deficits. Taken together with recent studies that have unveiled deficits in learning and predicting the spatial position of reinforcements,^15^^,^^56–58^ these findings pave new ways for interpreting the neglect syndrome and developing new rehabilitation protocols aimed at contrasting the attentional impairments suffered by these patients.

## Supplementary material

Supplementary material is available at *Brain Communications* online.

## Supplementary Material

fcab111_Supplementary_Data

## Abbreviations

HC =: healthy controls
MMN =: mismatch negativity
N+ =: neglect patients
N− =: non-neglect patients
RBD =: right brain damage
