# Supplementary material for: Deficits of hierarchical predictive coding in left spatial neglect
Source: Brain Commun. 2021 Jun 4;3(2):fcab111. doi: 10.1093/braincomms/fcab111 (PMC8209285; doi:10.1093/braincomms/fcab111)
Supplement: fcab111_Supplementary_Data [file fcab111_supplementary_data.zip › Original Submission.pdf]

OXFORD UNIVERSITY PRESS | Brain Communications

Deficits of hierarchical predictive coding in left spatial neglect

Journal:	*Brain Communications*
Manuscript ID	BRAINCOM-2021-007
Manuscript Type:	Original Article
Date Submitted by the Author:	08-Jan-2021
Complete List of Authors:	Doricchi, Fabrizio; Sapienza University of Rome, Psychology Departement ; IRCCS Santa Lucia, Centro ricerche Neuropsicologia<br>Pinto, Mario; IRCCS Santa Lucia, Centro ricerche Neuropsicologia<br>Pellegrino, Michele; Sapienza University of Rome, Psychology Departement<br>Marson, Fabio; Fondazione Patrizio Paoletti<br>Aiello, Marilena; SISSA, Area of Neuroscience<br>Campana, Serena; Auxilium Vitae Volterra SpA, Neurorehabilitation<br>Tomaiuolo, Francesco; University of Messina Faculty of Medicine and Surgery, Clinical and Experimental Medicine<br>Lasaponara, Stefano; Sapienza University of Rome, Psychology Departement
Keywords:	Spatial neglect, Right Brain Damage, EEG, MMN, P300, Predictive Coding

SCHOLARONE™
Manuscripts

Deficits of hierarchical predictive coding in left spatial neglect

Fabrizio Doricchi [a,b], Mario Pinto [ab], Michele Pellegrino [a,b], Fabio Marson[d], Marilena Aiello [f], Serena Campana [g], Francesco Tomaiuolo[e] and Stefano Lasaponara [a,b,c].

[a] Dipartimento di Psicologia 39, Sapienza Università di Roma, Via dei Marsi 78, 00185 Roma, Italy

[b] Laboratorio di Neuropsicologia dell'Attenzione, Fondazione Santa Lucia IRCCS, Neurorehabilitation Hospital, Via Ardeatina 306, 00179 Roma, Italy

[c] Dipartimento di Scienze Umane, Libera Università Maria Santissima Assunta – LUMSA, Borgo Sant'Angelo 13, 00193 Roma, Italy

[d] Fondazione Patrizio Paoletti – Assisi, Perugia, Italy

[e] Dipartimento di Medicina Clinica e Sperimentale, Università degli Studi di Messina, Italy

[f] Area of Neuroscience, SISSA, Trieste, Italy

[g] Auxilium Vitae – Neurorehabilitation Hospital, Volterra (Pisa), Italy

Corresponding Authors:

- Prof. Fabrizio Doricchi, PhD - *Dipartimento di Psicologia 39, Università degli Studi di Roma "La Sapienza", Via dei Marsi 78, 00185 Roma, Italy* – email: fabrizio.doricchi@uniroma1.it
- Stefano Lasaponara, PhD - *Dipartimento di Psicologia 39, Università degli Studi di Roma "La Sapienza", Via dei Marsi 78, 00185 Roma, Italy* – email

Running title: Predictive coding in spatial neglect

For Review Only

Abstract

Right brain-damaged patients with unilateral spatial neglect fail to explore the left side of space. Recent EEG and clinical evidence suggests that neglect patients might suffer deficits in predictive coding, i.e., in identifying and exploiting probabilistic associations among sensory stimuli in the environment. To gain direct insights on this issue, we focused on the hierarchical components of predictive coding. We recorded EEG responses evoked by central, left-side or right-side tones, that were presented at the end of sequences of four central tones. Left-side and right-side deviant tones produce a pre-attentive Mismatch Negativity (MMN) that reflects a lower-order prediction error for the "Local" deviation of the tone at the end of the sequence. Higher-order prediction errors for the frequency of these deviations in the acoustic environment, i.e. "Global" deviation, are marked by the P3 response. We show that when neglect patients are immersed in an acoustic environment characterised by frequent left-side deviant tones, they display no pre-attentive MMN both for left-side deviant tones and infrequent omissions of the last tone, while they have MMN for infrequent right-side deviant tones. In the same condition, neglect patients show no P3 response to "Global" prediction errors for deviant tones, including those in the non-neglected right-side, and omissions. In contrast to this, when right-side deviant tones are predominant in the acoustic environment, neglect patients have pre-attentive MMN both for right-side deviant tones and infrequent omissions, while they display no MMN for infrequent left-side deviant tones. Most importantly, in the same condition neglect patients show enhanced P3 response to infrequent left-side deviant tones, notwithstanding that these tones evoked no pre-attentive MMN. This latter finding indicates that "Global" predictions are independent of "Local" error-signals provided by the MMN. These results qualify deficits of predictive coding in the spatial neglect syndrome and show that neglect patients base their predictive behaviour only on statistical regularities that are related to the frequent occurrence of sensory events on the right side of space.

Keywords: Spatial neglect, Right brain damage, Predictive coding, MMN, P3 wave

For Review Only

Introduction

Attending relevant sensory events is facilitated if one can pick-up and exploit spatial and temporal contingencies in the environment. According to the "Predictive Coding Hypothesis" (Friston, 2005), the cortex's cytoarchitecture implements a multi-level hierarchical top-down prediction algorithm that helps to anticipate incoming sensory stimuli. Each hierarchical processing level stores an internal model of the environment, which is generated basing on statistical regularities that have governed past inputs. This model creates top-down predictions that regulate lower processing levels. Predictions are compared continuously with novel incoming inputs and when a mismatch is detected between predicted and actual events, a "prediction error" is transmitted from lower hierarchical levels to higher ones to adjust the internal model.

In the human, right brain damage (RBD) often produces left spatial neglect, which consists of the inability to attend the contralesional side of sensory, body and imagery space (Husain, 2007; Bartolomeo et al., 2007; Doricchi et al., 2008; Aiello et al., 2012; Aiello et al., 2013) and implies poor recovery outcome after stroke (Nijboer et al., 2013; 2014). Neglect most frequently follows lesions of parietal-frontal white matter (Doricchi & Tomaiuolo, 2003; Thiebaut de Schotten et al., 2005; Bartolomeo et al., 2007; Doricchi et al., 2007; Doricchi et al., 2008; Verdon et al., 2010; Lecce et al., 2015), though posterior lesions producing both left hemianopia and a concomitant splenial disconnection (Gaffan & Hornak, 1997; Lunven et al., 2015; Tomaiuolo et al., 2010) or lesions in the thalamus or the basal ganglia can also cause neglect (Damasio et al. 1980; Healton et al. 1982; Ferro et al. 1987; Karnath et al. 2002; Thiebaut de Schotten et al., 2012).

Although deficits of predictive coding in patients with neglect have never been directly investigated, a few studies offer insights on this issue. To evaluate pre-attentive processing in patients with neglect, in a pioneering study, Deouell and co-workers (2000) investigated the EEG responses evoked by infrequent final tones that deviated 30° medial to the position of a previous series of four standard tones that were presented 60° to the left or the right of the patient's mid-

For Review Only

sagittal plane. In the healthy brain, these "deviants" typically elicit a Mismatch Negativity (MMN) which is recorded over anterior-central derivations 130-150 ms after the presentation of the tone. The MMN is now considered a pre-attentive EEG marker of the prediction-error triggered by the detection of deviant tones (Wancongne et al., 2011). Deouell et al. (2000) found that patients with neglect have a reduced MMN for deviant tones on the left side of space, thus demonstrating a pre-attentive deficit and that the predictive coding of the same stimuli can be compromised in neglect.

In a recent study, we have explored the EEG correlates of attentional orienting in patients with neglect (Lasaponara et al., 2018; see also Lasaponara et al. 2019). We have found that these patients show a pathological drop in the amplitude of the P3a response evoked by targets that, following a right-pointing cue, were infrequently presented in the left side of space, i.e. invalid targets. Vice-versa, invalid targets in the attended right side of space produced a pathological enhancement of the P3a. Since the P3a is evoked by infrequent novel stimuli (Polich, 2007), we concluded that neglect patients have reduced novelty reaction to stimuli in the contralesional space and enhanced novelty reaction to stimuli in the ipsilesional space. Besides, patients with neglect displayed a generalised reduction of the P3b component evoked by all types of targets on the left side of space. Since the P3b marks the processing of the match or mismatch between expected and actual stimuli (i.e. probabilistic contextual updating; Polich, 2007), this latter result suggests that neglect patients are unable to monitor the probabilistic association between sensory cues that guide spatial attention and targets in the contralesional space.

Taken together, these few studies suggest that patients with neglect might suffer predictive coding deficits both at an early pre-attentive level and at a later processing level that helps to update the representation of probabilistic contingencies in the environment. Bekinschtein et al. (2009) have devised a behavioural paradigm that can help to test and eventually dissociate pre-attentive and contextual updating predictive deficits. In this task, two levels of auditory predictions are specified. The first lower-order level is defined as "Local" and has to do with the perception of

For Review Only

regularities/irregularities in a sequence of five tones. Regular-standard sequences are made of five identical tones, e.g., AAAAA, while in irregular-deviant sequences, the last tone is different from the four previous ones, e.g., AAAAB, or is missing, e.g., AAAA_. Deviants and omitted tones evoke a MMN. The second and higher-order level of prediction is defined as "Global" and has to do with the capability of recognising that in a given block of trials, regular sequences are more frequent than irregular ones, or vice versa, and elaborating corresponding sensory expectations. At the electrophysiological level, the occurrence of an infrequent sequence triggers a P3 response.

Here, using a variant of the paradigm devised by Bekinschtein et al. (2009), we have manipulated both the "Local" and "Global" spatial-auditory features of the environment to test pre-attentive (MMN) and contextual-updating (P3) deficits in patients with left spatial neglect. To this aim, we investigated the brain electrophysiological responses that are evoked by "Local" and "Global" auditory irregularities, with specific reference to irregularities produced by the infrequent displacement of tones in the left or right side of space. In different blocks of trials, left-side displacements were frequent and right-side displacements infrequent or vice versa. This allowed investigating whether patients with neglect can develop spatial predictive coding based on the more or less frequent occurrence of events in each lateral space. As an example, one might hypothesise that when deviations are more frequent in the neglected left side of space, patients with neglect can still infer this probabilistic distribution from the infrequent occurrence of events in the attended right side, and accommodate predictive coding for the two sides of space or, eventually, only for the right-side. Alternatively, one might hypothesise that in these patients, predictive coding is exclusively based on the predominant occurrence of events in one of the two lateral sides of space. In this case, due to their pathological bias of spatial attention, patients with neglect could generate spatial predictions only basing on the more frequent occurrence of events in the right side of space though not when events are more frequent in the left side of space. Recording of the MMN and P3 EEG markers of predictive coding helps in contrasting and verifying these different scenarios.

For Review Only

Material & methods

Participants

Patients were examined at the rehabilitation hospital Fondazione Santa Lucia IRCCS (Rome). Patients with bilateral strokes, signs of dementia, or history of previous neurological illness were excluded. Two groups of patients completed the experimental protocol: thirteen right-brain-damaged patients with left spatial neglect (N+) and fourteen right-brain-damaged patients without neglect (N-). In addition, sixteen age-matched healthy participants were tested as controls (HC). Patients and participants were all right-handed and had normal or corrected-to-normal visual acuity. At the time of clinical and experimental examination, all patients were free from confusion and temporal or spatial disorientation. Visual fields were tested with standard kinetic Goldmann perimetry. All patients had intact visual fields. N+ and N- patients did not differ in time elapsed from stroke onset ($F_{(1,11)} = 3.2$, $P = 0.1$; mean = 47 days). Age was equivalent among N+, N- and HC ($F_{(2,40)} = 2.4$ $P = 0.6$; mean age: HC = 58.3; N+ = 67.6; N- = 59.2 years). Unilateral neglect was assessed with a battery of six standardised tests (for details see Supplementary Material): (1) Line bisection (Pizzamiglio et al., 1992; Rotondaro et al., 2015); (2) Letter cancellation (Diller et al., 1974); (3) Line cancellation (Albert, 1973); (4) Star cancellation (Halligan et al., 1990); (5) Sentence reading test (Pizzamiglio et al., 1992); (6) Wundt–Jastrow area illusion test (Massironi et al., 1988). Patients who failed on at least two out of the six tests were classified as suffering left spatial neglect. Auditory neglect was separately assessed through a “straight-ahead” acoustic task (Vallar et al. 1995). This task requires to judge the alignment of unseen acoustic stimuli, moving along a 180° arc, to the subjective head-body mid-sagittal plane (for details, see Supplementary Materials). Clinical and demographic data are reported in **Table 1.** Patients and controls gave their informed consent for participating in the study that was approved by the Institutional Ethical Committee of the Fondazione Santa Lucia IRCCS.

For Review Only

*** Insert Table 1 about here ***

Patients Group	Sex		Age (y)	Stroke onset (days)	Line bisection (200 mm) rightward deviation (mm)	Letter cancellation		Line cancellation		Star cancellation		Sentence reading test	Wundt-Jastrow illusion (unexpected responses)		Deviation of the subjective acoustic "straight ahead"
						Left	Right	Left	Right	Left	Right		Left	Right	
**N-; n = 14**															
	M=11	**Mean**	59.2	40.1	0.01	52(53)	50.4(51)	10.9(11)	9.9(10)	26.3(27)	26.8(27)	5.6(6)	1.1(20)	0(20)	1.2°
	F=3	**S.D.**	15.4	15.8	5.2	1.1	0.9	0.2	0.2	1.3	0.6	0.9	2.7	0	3.3°
**N+; n = 13**															
	M=6	**Mean**	67.6	56	19.1	29(53)	43.3(51)	8.2(11)	9.2(10)	9(27)	21.8(27)	3.5(6)	12.4(20)	1.8(20)	17.4°
	F=7	**S.D.**	11.1	27.6	20.2	17.2	14	3.4	2.1	8.8	7	2.5	6.7	2.3	20.6°
**HC; n = 16**															
	M=7	**Mean**	58.3												-0.9°
	F=9	**S.D.**	11.1												1.7°

Table 1. Clinical and demographic group data of RBD patients with left spatial neglect (N+), without left spatial neglect (N-), and healthy controls (HC). Maximal scores for each test are reported in parenthesis. In the acoustic task, positive scores indicate rightward deviation of the subjective auditory "straight-ahead" and negative scores leftward deviation.

Lesion mapping

Individual 1.5 T MRI scans were corrected for inter-individual differences in brain size and brain volume orientation, using a transformation into the standardised MNI space using the software REGISTER (http://www.bic.mni.mcgill.ca/ServicesSoftwareVisualization/Register). Damaged areas were mapped through the DISPLAY software (http://www.bic.mni.mcgill.ca/software/Display/Display.html). The probability maps of N+ and N- groups are reported in **Fig. 2**. In each experimental group, the MNI coordinates of the centroids of maximal lesion overlap areas were defined using the command DISPLAY. To check whether peaks of lesion overlap encroached upon white matter pathways, we used the DSI Studio software (http://dsi-studio.labsolver.org) to superimpose lesion peaks on the three-dimensional reconstruction of white matter pathways taken from the diffusion tensor atlas by Yeh et al. (2018). See Supplementary Material for details on lesion mapping procedures.

For Review Only

Procedure and stimuli

Participants were tested with the head comfortably blocked by a chin rest, in a dimly lit, sound attenuated, and electrically shielded room. Auditory stimuli were presented using in-ear headphones. Participants were only asked to listen to these sounds and keep their gaze stable on a central fixation cross presented on a video monitor (22 inches) at a viewing distance of 57.5 cm. The presentation of auditory stimuli was performed with E-prime software (Schneider *et al.,* 2002).

The task consisted of an auditory oddball-paradigm in which the location of a sound shifts unpredictably from a central location, aligned to the body-midsagittal plane, to the left or the right side of egocentric space. We used an inter-aural time delay (ITD) between the left and the right to induce subjective leftward or rightward ~90° shift in the sound location in the horizontal plane. All other spectral, amplitude and duration parameters of the sound were kept constant. Each trial lasted 850 ms and included a series of 50 ms tones with 70 dB intensity and a stimulus onset asynchrony of 150 ms. Four different types of trials were presented: a) five identical tones presented at the central midline (*local standard*, denoted MMMM**M**); b) four identical tones at midline followed by a deviant tone in the left space (**L**) (*left local deviant*, denoted MMMM**L**); c) four identical tones at midline followed by a deviant tone in the right space (**R**) (*right local deviant*, denoted MMMM**R**); d) four identical tones with no fifth tone (*Omission*: MMMM_); These trial types were organised and presented within six different semi-randomised experimental blocks of ~4-min duration, with variable inter-trial intervals (700 -1,000 ms), each relating to a different variation of the "Global" predictive characteristics of the stimuli. Similar to Bekinschtein et al. (2009), each block of trials started with the repetition of 20 identical trials, i.e., *frequent* series (MMMM**M** *local standard* or *left* and *right local deviant* MMMM**L**/MMMM**R** in different blocks), to establish the "Global" regularity. These were followed by 100 trials with 70% of *frequent* series, 20% *infrequent* series (MMMM**L** or MMMM**R** when the *frequent* series is MMMM**M** and vice versa), and 10% of the *Omission* series. The structure of the different block of trials is depicted in **Fig. 1** (see

For Review Only

Supplementary Material for details). The whole experiment included two experimental sessions (6 blocks of trials in each session for 1440 trials) that were run in different days and separated by a one-two day interval.

*** Insert Figure 1 about here ***

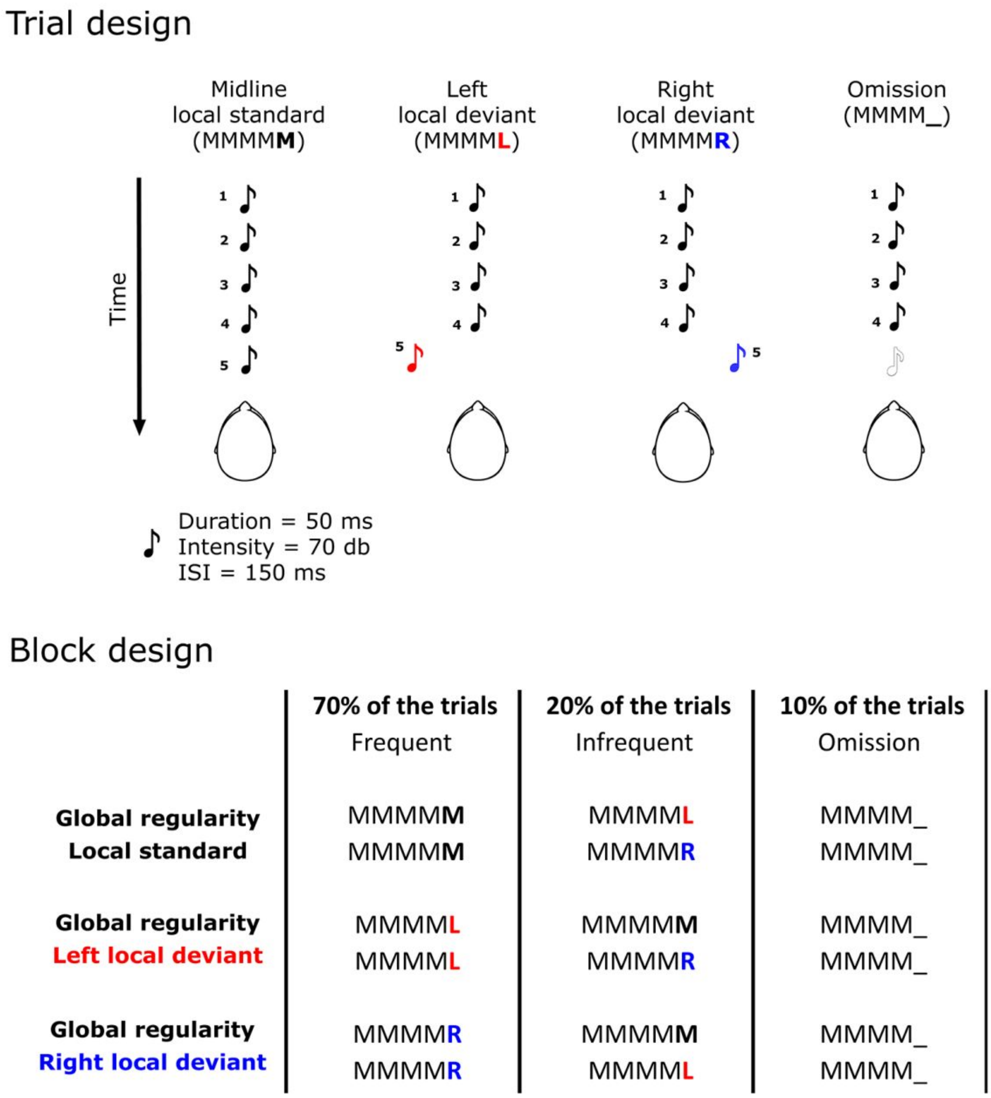

Fig.1: Structure of the four types of auditory trials and the six experimental blocks/experimental-conditions used in the study

EEG recording and pre-processing

The EEG was recorded using a Brain Vision system from 64 electrodes placed according to the 10–10 system montage. All scalp channels were online referenced to Fcz. Horizontal eye movements were monitored with a bipolar recording from electrodes at the left and right outer canthi. Blinks and vertical eye movements were recorded with an electrode below the left eye, which was referenced to site Fp1. The EEG from each electrode site was digitised at 250 Hz with an amplifier bandpass of 0.01–60 Hz, including a 50 Hz notch filter, and was stored for off-line averaging. Continuous EEG was recalculated against the average reference and successively segmented in epochs lasting 1600 ms, locked to the first tone of the trial sequence. A time period of 200 ms before this event was used for baseline-correction. Before automatic artefact rejection, the ocular correction was performed accordingly to Gratton & Coles algorithm (Gratton *et al.,* 1983). Artefact rejection was performed before signal averaging to discard epochs in which deviations in eye position, blinks, or amplifier blocking occurred. All epochs in which EOG amplitudes and EEG amplitudes were greater than ±60 mV were excluded from further analysis. On average, 4%, 6%, and 7% of the trials were rejected for violating these artefact criteria in HC, N- and N+ group, respectively.

For Review Only

Statistical analyses

Clinical and demographical data.

We compared the clinical performance between the two groups of patients by entering the individual score of Line bisection, and Sentence reading test in a series of unpaired two-tailed T-test with a p-level set to 0.05. Performances in the Letter cancellation, Line cancellation, Star Cancellation, and the Wundt-Jastrow Area Illusion test were compared through a series Group (N-,

N-) x Target Side (Left, Right) repeated-measures ANOVA. Performances in the "straight-ahead" acoustic task were compared through a one-way between groups (HC, N-, N-) ANOVA.

Lesion analyses

Descriptive statistical comparisons of lesion mapping were run by subtracting the probability map of the N- group from that of the N+ group and by comparing, with Fisher exact test, the frequency of damage occurrence at the centroids of the areas of maximal lesion overlap. Lesion probability maps resulting from this subtraction and the corresponding MNI coordinates of centroids of lesion overlaps are reported in **Fig. 2**.

For Review Only

ERPs analyses.

"Local" effect – MMN

To check for the electrophysiological correlates of "Local" deviance, we averaged together, and independently from their global probability, all trials in which the last tone deviated from the midline (*local deviant*). Separate averages were calculated for left and right deviants. Similarly, we averaged all *local standard* trials (Bekinschtein et al., 2009; see **Fig. 1**). In each of these experimental conditions, the amplitude of the MMN component was measured as the mean activity change with respect to a 200 ms pre-stimulus baseline over Fz, FCz, Fc1/2 pools of derivations in a time windows of 170 - 220 ms after the onset of the last tone of the trial sequence.

The selection of time windows and derivations used for the analysis of MMN was made using statistically defined t-maps that highlight scalp sites where ERPs components were significantly present for a minimum amount of time (Sergent et al., 2005; Bekinschtein et al., 2009; Lasaponara et al., 2015; for details see Supplementary Material). We then subtracted the mean amplitude of MMN recorded in *local standard* trials from that of *local deviant* trials. Separate

subtractions were run for left and right deviants. Individual subtractive data were entered in a Group (HC, N- and N+) x Side of deviance (Left, Right) repeated-measures ANOVA.

In a second analysis, we checked the "Local" effects that were produced by a 180° deviation of the last tone in auditory space. To this aim we compared: a) the amplitude of the MMN evoked by *infrequent left local deviant* trials (MMMM**L**) with that evoked by *frequent right local deviant* ones (MMMM**R**) and vice versa; b) the amplitude of the MMN evoked by *omissions* with that elicited by frequent *left* and *right local deviant* trials. Individual data were entered in a Group (HC, N- and N+) x Side of local deviance (Left, Right) x Frequency (Freq, Infreq, Omission) repeated-measures ANOVA. In a final analysis, we compared the MMN evoked by *infrequent omissions* of the last tone (MMMM_) with that elicited by *frequent local standard* sequences (MMMM**M**).

In all cases post-hoc tests were corrected for multiple comparisons using Bonferroni correction.

"Global" effect – P3b

"Global" effects were measured by averaging, independently from local deviance, all *frequent* (i.e. 70% probability) and all *infrequent* (i.e. 20% probability) trials separately for the left and right side of space. In each of these experimental conditions, the amplitude of P3b components was measured as the mean activity change with respect to a 200 ms pre-stimulus baseline over Pz, P7/8, P3/4 pools of derivations in a time window of 450 - 750 ms after the onset of the last tone of the sequence. The selection of time windows and derivations used for the analysis was made using statistically defined t-maps (see Supplementary Material). Mean amplitude of the P3b recorded in all *frequent* trials was subtracted from the mean amplitude of the P3b recorded in all *infrequent* trials, separately for the left and the right side of space. Individual differential data were entered in a Group (HC, N- and N+) x Side of global infrequency (Left, Right) repeated-measures ANOVA.

For Review Only

Again, we investigated the presence of “Global” effects when the last tone shifted 180° the auditory space. As in the case of “Local” analysis, we compared: a) the mean amplitude of the P3b evoked by *infrequent left local deviant* trials (MMMM**L**) with that evoked by *frequent right local deviant* ones (MMMM**R**) and vice versa; b) the mean amplitude of the P3b evoked by *omissions* with that elicited by *frequent left* and *right local deviant* trials. Individual data were entered in a Group (HC, N- and N+) x Side of global deviance (Left, Right) x Frequency (Freq, Infreq, Omission) repeated-measures ANOVA. Finally, we compared the P3b evoked by *infrequent omissions* (MMMM_) when compared to *frequent local standard* sequences (MMMM**M**).

In all cases post-hoc tests were corrected for multiple comparisons using Bonferroni correction.

For Review Only

Data availability

The data that support the findings of this study are available on request from the corresponding authors. The data are not publicly available, as they include information that could compromise the privacy of the research participants.

Results

Clinical results

A series of between-group comparisons, showed that compared to N-, N+ patients had significant rightward spatial biases in all neglect tasks (see **Table 1**). N+ had a higher rightward bias in line bisection ($t_{(25)}$ = -3.4, p = 0.002, unpaired t-test) and showed a higher number of left side omissions in the Sentence reading task ($t_{(25)}$ = 2.9, p = 0.007, unpaired t-test). In the Letter cancellation ($F_{(1,25)}$ = 13.4, p = 0.001, η_p^2= 0.34), Line cancellation ($F_{(1,25)}$ = 12.9, p = 0.001, η_p^2=

0.34), Star Cancellation ($F_{(1,25)}$ = 34.4, p = 0.0000, η_p^2= 0.57) and in the Wundt-Jastrow Area Illusion task ($F_{(1,25)}$ = 20.9, p = 0.0001, η_p^2= 0.45), N+'s performance differed from that of N- more for stimuli positioned in the left side of space than for stimuli placed in the right side of space, as indexed by significant Group x Side interactions.

N+ patients showed a significant rightward deviation of the subjective acoustic "straight-ahead" both as compared to HC and N- ($F_{(2,40)}$ = 12.4, p < 0.001; HC: - 0.9°; N-: +1.2° and N+: +17.4°). No difference was present between HC and N- (p >.05).

Anatomical results

The lesion probability maps resulting from the subtractions between N+ and N- showed one cluster ranging from 40% to 80% of lesion overlap in N+ and no corresponding overlap, i.e., 0%, in N- patients (Fischer exact test, p = 0.006). The maximal lesion overlap (i.e., 75%) was located in the white matter underneath the inferior parietal-frontal cortex in the region of the superior longitudinal and arcuate fasciculi (See **Fig. 2:** Peak A, MNI coordinates: 27, -15, 35). This finding confirms the role of parietal-frontal white matter disconnection in spatial neglect (Doricchi and Tomaiuolo, 2003; Thiebaut De Schotten et al., 2005; Verdon et al., 2010).

*** Insert Figure 2 about here ***

For Review Only

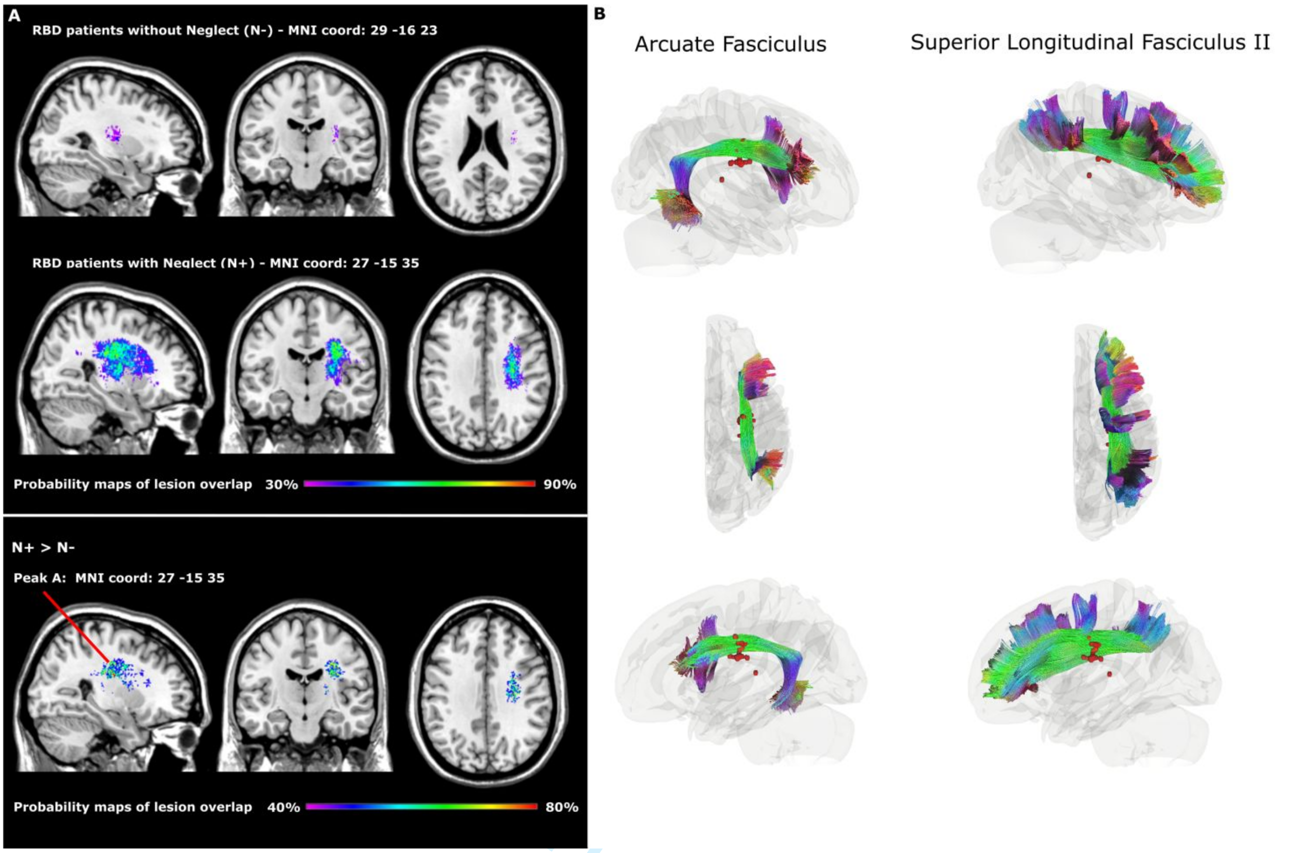

Fig.2: A) Probability maps of lesion overlap: First row, patients without neglect (N-); second row, patients with neglect (N+). The third row represents the peaks of lesion overlap resulting from the N+ minus N- subtraction. Note, the main peak of the subtraction (MNI coord: 27, -15, 35) is located in the superior longitudinal and arcuate fasciculi. **B)** Tridimensional reconstruction of the superior longitudinal and arcuate fasciculi, according to the atlas by Yeh et al. (2018). Red circles represent the highest (70-75%) peaks of lesion overlap resulting from the N+ minus N- subtraction.

view Only

Electrophysiological results

"Local" effect – MMN component

A main effect of Group ($F_{(2,40)}$ = 5.5, p = 0.007, η_p^2= 0.21) showed that the amplitude of MMN was generally reduced in N+ (-0.07 µV) with respect to HC (-0.79 µV; p < 0.01) and N- (-0.84 µV; p < 0.01) while no difference was present between the latter two groups. A significant Group x Side of deviance interaction ($F_{(2,40)}$ = 4.9, p = 0.01, η_p^2= 0.19) showed that in N+ the MMN was suppressed in response to *left-side deviant* stimuli (HC = - 0.75 µV; N- = - 0.82 µV; N+ = 0.19

μV; both $p < 0.01$) while the amplitude of MMN evoked by *right-side deviants* was comparable to that of N- and HC (HC = - 0.84 μV; N- = - 0.86 μV; N+ = - 0.33 μV; all p = n.s.; see **Fig. 3**).

In the case of MMN evoked by stimuli that deviated 180° degree in auditory space, a significant Group x Side x Frequency interaction ($F_{(2,40)} = 2.8$, $p = 0.03$, $\eta_p^2 = 0.12$) showed that independently of stimulus frequency (i.e. 70% vs. 20% of trials), in N+, a significant MMN was only found in response to *right-side deviants* (*frequent* MMMM**L** = -0.41 μV vs. *infrequent* MMMM**R** = -0.83 μV, p = 0.01; *frequent* MMMM**R** = -0.89 μV vs. *infrequent* MMMM**L** = -0.43 μV, p = 0.005; see **Fig. 4**). This was not found in HC and N-, who displayed comparable MMN for *left-side* and *right-side deviant*, both for *frequent* and *infrequent* stimuli (all p = n.s.; see **Fig. 4**). The same interaction showed that during blocks of trials with *frequent right-side deviants,* in N+ patients *omissions* produced an MMN that was comparable to that evoked by the *right-side deviant*s (*frequent* MMMM**R** = -0.89 μV vs *omissions* = -0.84 μV, p = n.s.; see **Fig. 4**). In all other cases, *omissions* produced no significant MMN.

Finally, in all groups, when compared with *local standard*, no MMN was found in response to *omissions* (all F < 1, all p = n.s.; see **Supplementary Fig. 2**).

For Review Only

*** Insert Figure 3 about here ***

*** Insert Figure 4 about here ***

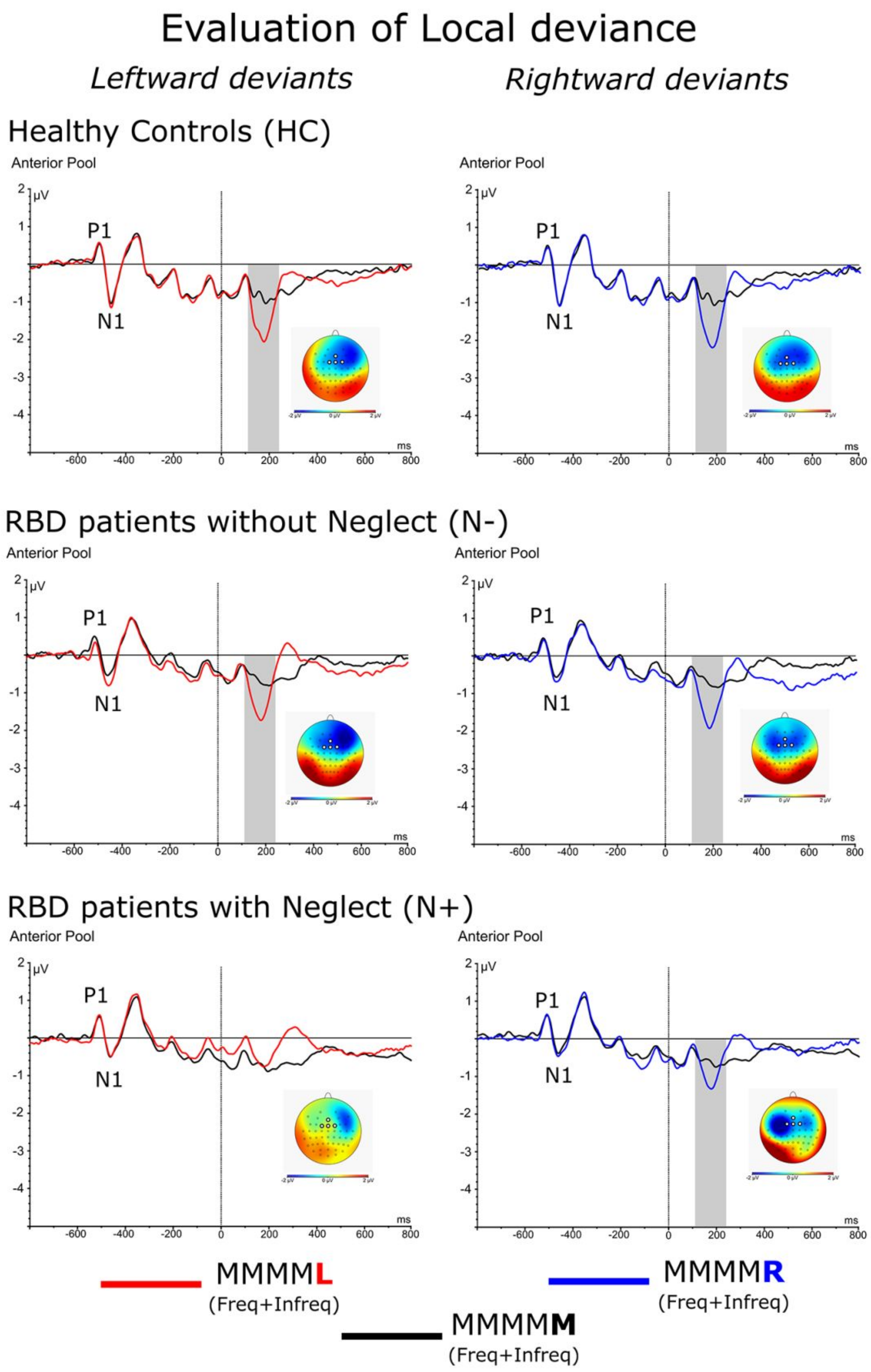

Fig. 3: Waveforms and relative differential scalp topographies of the MMN evoked by the *left* and *right local deviants* as compared to *local standard* in HC, N- and N+. Grey shades indicate post-stimulus onset time-intervals in which a significant statistical difference is present.

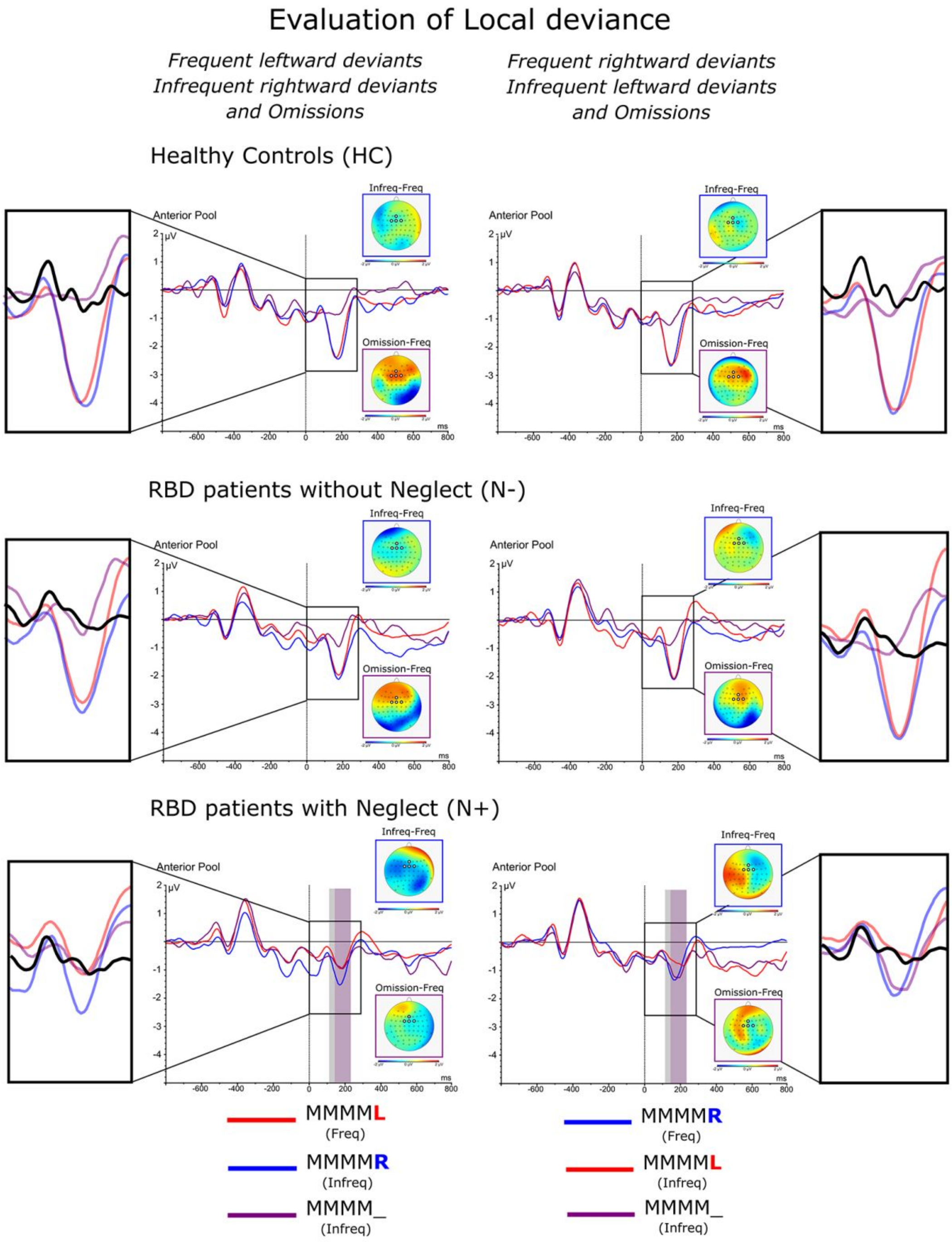

Fig. 4: Waveforms and relative differential scalp topographies of the MMN component evoked by *frequent* and *infrequent local left* or *right deviant* sequences and *omissions* in HC, N- and N+. Grey shades indicate post-stimulus onset time-intervals in which a significant statistical difference is present between *frequent* and *infrequent* trial type. Purple shades indicate post-stimulus onset time-intervals in which a significant statistical difference is present between *frequent* and *omission* trial type. Zoomed squares show the comparison with *local standard* (MMMM**M**) sequences.

"Global" effect – P3b component

A main effect of Group ($F_{(2,40)}$ = 5, p = 0.01, η_p^2= 0.20) highlighted a generalised reduction in P3 amplitude in N+ (0.21 μV), as compared both to HC (0.40 μV; p < 0.01) and N- (0.44 μV; p < 0.005). No difference was present between these latter groups. In addition, a significant Group x Side of global infrequency interaction ($F_{(2,40)}$ = 5.7, p = 0.006, η_p^2= 0.22) showed that, in N+ the P3b was selectively suppressed in response to *infrequent* stimuli presented in the left side of space (HC = 0.39 μV; N- = 0.44 μV; N+ = 0.05 μV; both p < 0.005), while equivalent right-side stimuli evoked a P3b that was comparable to that of HC and N- (HC = 0.41 μV; N- = 0.44 μV; N+ = 0.37 μV; all p = n.s. see **Fig. 5**).

The analysis of "Global" effects in blocks of trials with final tones that deviated of 180° degrees in the acoustic space showed that in blocks with *frequent left-side deviants*, *infrequent right-side deviants* produced a significant P3b in HC (*frequent* MMMM**L** = 0.10 μV vs. *infrequent* MMMM**R** = 0.67 μV, p = 0.001) and N- (*frequent* MMMM**L** = 0.25 μV vs. *infrequent* MMMM**R** = 0.89 μV, p = 0.001) though not in the N+ (*frequent* MMMM**L** = 0.1 μV vs. *infrequent* MMMM**R** = 0.11 μV, p = n.s.; Group x Side of global deviance x Frequency interaction: $F_{(2,40)}$ = 6.6, *p* = 0.001, η_p^2= 0.24; see **Fig. 6**). In contrast, in blocks with *frequent right-side deviant*s, both *infrequent left-side deviants* and *omissions* produced a significant P3b in all groups (HC: *frequent* MMMM**R** = 0.14 μV vs. *infrequent* MMMM**L** = 0.66 μV, p = 0.001; N-: *frequent* MMMM**R** = 0.08 μV vs. *infrequent* MMMM**L** = 0.80 μV, p = 0.001; N+: *frequent* MMMM**R** = 0.09 μV vs. *infrequent* MMMM**L** = 1.34 μV, p = 0.001; see **Fig. 6**).

In HC, *omissions* produced a significant P3b both when compared to *frequent lef-side and frequent right-side deviant*s (*frequent* MMMM**L** = 0.10 μV vs. *omission* = 0.66 μV, p = 0.001; *frequent* MMMM**R** = 0.14 μV vs. *omission* = 0.65 μV, p = 0.001). It is interesting to note that, in these cases, the P3b component produced by *omissions* was delayed of about 200 ms with respect to that evoked by *infrequent deviants*. In contrast, in N- and N+ *omissions* produced a significant P3b

For Review Only

only in blocks with *frequent right-side deviants* (N-: *frequent* MMMM**R** = 0.08 μV vs. *omission* = 0.76 μV, p = 0.001; N-: *frequent* MMMM**R** = 0.09 μV vs. *omission* = 0.57 μV, p = 0.001). Notably, in N+ the P3b evoked by *omissions* was smaller than that evoked by *infrequent left-side deviant* stimuli (*omission* = 0.57 μV, p = 0.001 vs. *infrequent* MMMM**L** = 1.34 μV, p = 0.001; see **Fig. 6**).

Finally, during block of trials with *frequent local standard* stimuli, *omissions* produced a significant P3b component in HC (*frequent* MMMM**M** = -0.16 μV vs. *omission* = 0.94 μV, p = 0.006), and N- (*frequent* MMMM**M** = -0.12 μV vs. *omission* = 0.68 μV, p = 0.04) though not in the N+ (Group x Frequency interaction, $F_{(2,40)} = 5.39$, p = 0.008, η_p^2= 0.21; *frequent* MMMM**M** = 0.18 μV vs. *omission* = -0.5 μV, p = n.s.; **see Supplementary Fig. 2**).

*** Insert Figure 5 about here ***

*** Insert Figure 6 about here ***

For Review Only

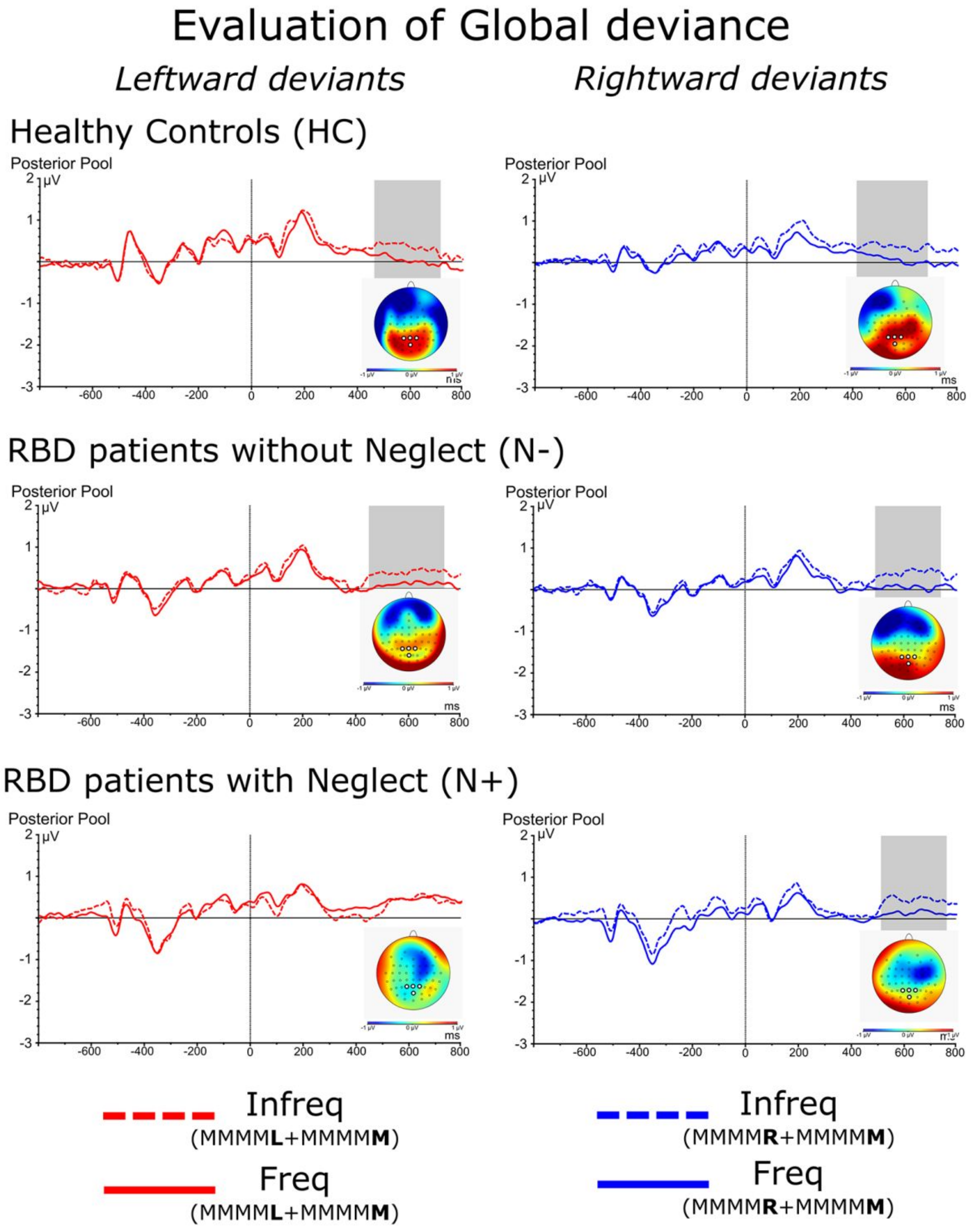

Fig.5: Waveforms and relative differential scalp topographies of the P3b component evoked by *frequent* and *infrequent* stimuli presented in the left and on the right side of space in HC, N- and N+. Grey shades indicate post-stimulus onset time-intervals in which a significant statistical difference is present.

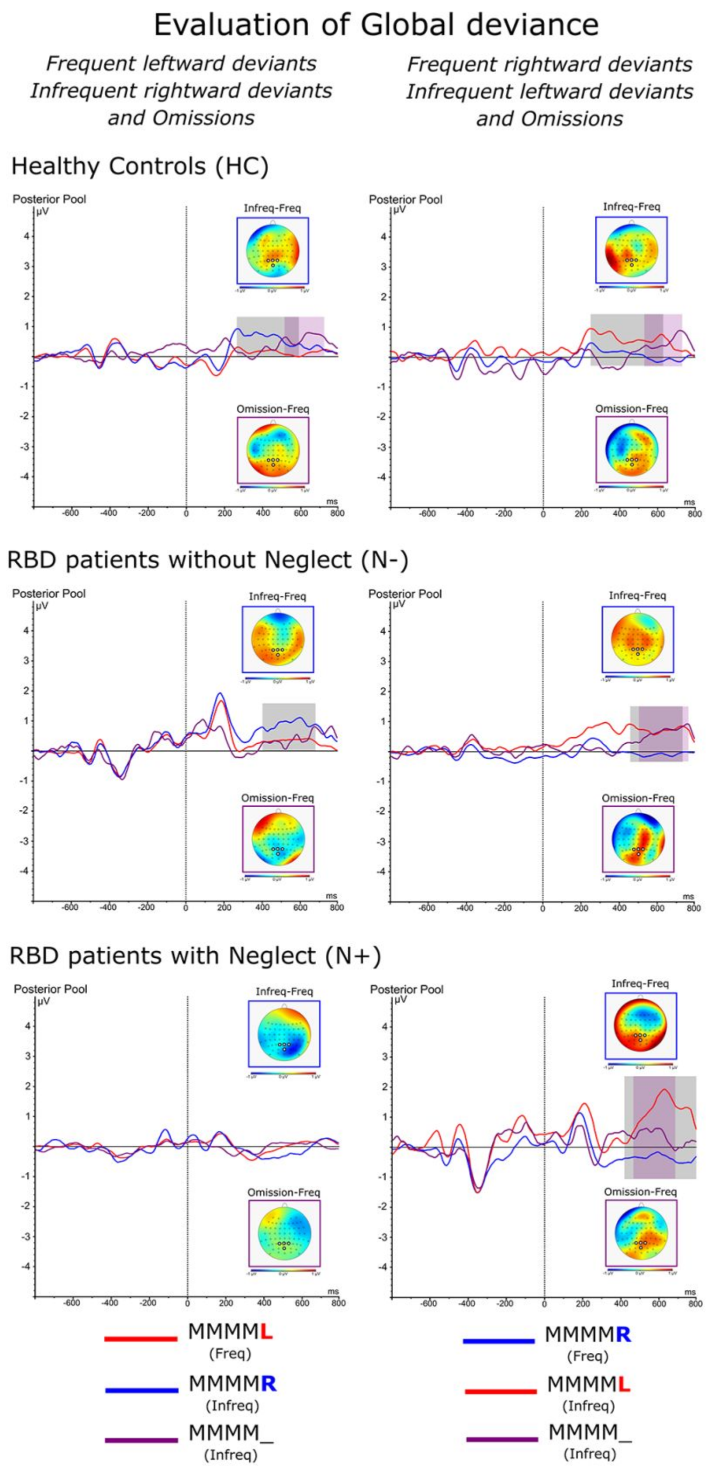

Fig.6: Waveforms and relative differential scalp topographies of the P3b component evoked in response to the *frequent* and *infrequent local left* or *right deviant* sequences and *omissions* in HC, N- and N+. Grey shades indicate post-stimulus onset time-intervals in which a significant statistical difference is present between *frequent* and *infrequent* trial type. Purple shades indicate post-stimulus onset time-intervals in which a significant statistical difference is present between *frequent* and *omission* trial type.

Discussion

To gain novel insights into the spatial neglect syndrome, we investigated the ability of neglect patients to build-up and exploit predictions on the probability that an acoustic stimulus will occur in the neglected left or in the attended right side of space. To this aim, we used a variant of an auditory task (Beckinstein et al., 2009) that allows studying the electrophysiological correlates of predictive coding at different processing levels.

Two preliminary findings are relevant. First, compared to controls, patients with neglect showed intact primary sensory processing of both left-side and right-side deviant tones, as demonstrated by the comparable latency and amplitude of the P1 and N1 responses evoked by these tones. Second, in neglect patients, left-side deviants evoked a MMN that was smaller in amplitude than that evoked by right-deviants. This result replicates the pioneering observations by Deouell and co-workers (2002).

As for the specific aims of our study, we found that in patients with neglect, the reduction in amplitude of the MMN evoked by left-side deviant tones matched an interesting modulation of the MMN evoked by omissions of tones. When in a block of trials, the most frequent event was represented by left-side deviants, the omission of the last tone evoked a reduced MMN that was equivalent in amplitude to that evoked by left-deviants. In contrast, when right-deviant tones were the most frequent event in a block of trials, the amplitude of the MMN evoked by omitted tones increased and reached the amplitude of the MMN evoked right-deviants. These results show that the pre-attentive processing of the same sensory omissions changed utterly depending on the predominant presentation of tones in the left or right side of space, thus exemplifying the profound effects that the probabilistic sensory context can exert on the behaviour of neglect patients.

The influence that the most frequent event in a block of trials played in the modulation of ERPs in patients with neglect, was even more highlighted by the analyses of the P3 response that marks “Global” predictive coding. In this case, when left-deviant tones were most frequent in a

For Review Only

block of trials, no P3 was evoked by infrequent right-deviants and omissions. In striking contrast with these results, when right-deviant tones were most frequent in a block of trials, both infrequent omissions and infrequent left-side deviants enhanced the amplitude of the P3. These findings points-out the influence that “Global” predictive coding exerts on late, attentive responses in patients with neglect. The same results show that in these patients, predictions on the probabilistic spatial distribution of sensory events are only generated when sensory events most frequently occur in the right ipsilesional space. This latter conclusion is based on the finding that when acoustic events were more frequent in the left side of space, no P3 marker of “Global” prediction was elicited by infrequent right-deviant tones in the attended space.

Interestingly, compared to both healthy controls and patients with neglect, patients without neglect showed a partial disturbance of “Global” predictive coding in the left side of space, because when in a block of trials left-deviant tones were predominant, they displayed a P3 response only to right-deviant tones though not to omitted ones. It is important to consider that this was in contrast to what happened in the same group when right-deviant tones were predominant in a block of trials. In this case, both left-deviant and omitted tones evoked the P3.

In terms of hierarchical predictive coding (Friston, 2005), the MMN marks the “Local” short-term deviance of a stimulus in a sequence of stimuli and the corresponding release of a prediction-error signal. These signals eventually feed-up the formulation of more complex “Global” long-term predictions that deal with the occurrence of local deviants across the entire pool of sequences presented in a block of trials. In this latter case, prediction errors are indexed by the P3 component. Within this framework, the findings in neglect patients pointed out a clear double dissociation between “Local” and “Global” predictive coding. First, when left-side deviants were predominant in a block of trials, infrequent right-side deviants elicited a normal local MMN error signal though no corresponding P3 response, i.e., no building-up of “Global” predictions (MMN in Figure 4, blue line in the bottom-left panel; P3b in Figure 6, blue line in the bottom-left panel).

For Review Only

Second, and most importantly, when rightward deviants were predominant in a block of trials, an opposite dissociation was found. In this case, neglect patients displayed no pre-attentive MMN together with enhanced P3b for infrequent leftward-deviant tones (MMN in Figure 4, red line in the bottom-right panel; P3b in Figure 6, red line in the bottom-right panel). This latter dissociation points out that "Global" predictive coding does not necessarily depend on local MMN signals and suggests that "Local" and "Global" predictions are elaborated with a high degree of functional independence. Past studies in healthy participants have shown that frequent pitch-deviants that evoke no P3 can still trigger a MMN, thus highlighting that lower-level "Local" predictions are free from the influence of higher-level "Global" ones (Bekinschtein et al. 2009; Wancogne et al., 2011). The other way round, the same studies showed that infrequent sequences of five identical tones evoke no MNN while they trigger a clear P3b response (Wancogne et al., 2011). The results of pharmacological, behavioural and brain imaging studies converge on the functional dissociation between the MMN and the P3 response. In humans, serotonin reuptake inhibitors increase the amplitude of the MMN without modifying the P3 (Wienberg et al., 2010), while low doses of ethanol reduce the amplitude of the MMN without affecting the P3b (Jääskeläinen et al., 1995). Sussman (2007) showed that tone sequences with infrequent pitch deviations elicit both the MMN and P3 when participants are asked to focus on single pitch-deviant tones. In contrast, the P3 is maintained, while the MMN is no longer elicited, when participants are asked to focus on the occurrence of sequences rather than on single pitch deviations. Finally, in a MEG study, King et al. (2014) showed that local acoustic deviants evoke a sequence of short-lived patterns of brain activity early after the end of the tone sequence (120 to 200 ms after the fifth tone). In comparison, violations of "Global" auditory regularities lead to a later (150 to 700 ms after the fifth tone), more stable and long-lasting pattern of activity, that engage working memory resources in parietal and prefrontal areas (King et al., 2014).

In our study, to explore the automatic building up of "Local" and "Global" expectations, we avoided requiring the report of the presence or localisation of the last tone in a sequence. Lack of

For Review Only

reports limits the possibility of evaluating whether patients, with and without neglect, consciously perceived these tones when no MMN, P3 or both were evoked. It has been proposed (Dehaene and Changeux, 2011), and it is currently debated (Koch et al., 2016), that the P3 marks the conscious processing of sensory stimuli. In neglect patients, the lack of the P3 in response to all types of tones when leftward deviant tones were predominant in a block of trials, is compatible with the view that these patients have reduced awareness of events in the left side of space. Nonetheless, our study also demonstrates a relevant effect of predictive coding on the P3 evoked by left-side deviant tones in patients with neglect. Indeed, when these stimuli were infrequently alternated with frequent rightward-deviant ones, they produced an enhancement of the P3b despite eliciting no corresponding pre-attentive MMN processing. How to interpret this finding? A plausible hypothesis is that since these tones were released binaurally so that an acoustic input was always presented in the attended right side of space/ear, they were interpreted as a different and novel type of spatially deviant tones. Neglect patients often display alloacusia (Bender and Diamond, 1965; Pavani et al., 2004), where acoustic stimuli in the left side of space are reported as coming from the ipsilesional right side. Inquiries on the ventriloquist illusion have highlighted that when a concurrent visual stimulus shifts the subjective position of deviant tones to that of standard ones, the MMN is suppressed (Colin et al., 2002). We hypothesise that the lack of pre-attentive MMN processing and the enhanced P3b attentive processing contribute to alloacusia of left-side acoustic stimuli in neglect patients.

Based on neuro-computational simulations of the consequences of brain lesion on explorative ocular behaviour, Parr and Friston (2017; 2018; see also Parr et al., 2018) have recently advanced a series of hypotheses on predictive coding in patients with neglect. They proposed that due to parietal-frontal white matter disconnection in the right hemisphere, neglect patients would be biased away from the contralesional side of space because they expect low novelty reduction and uncertainty resolution from fixating locations in that side of space. Put in short, the left side of space would offer poor changes in their beliefs on the probabilistic contingencies in that side of

For Review Only

space, i.e. poor epistemic affordance. This view is generally compatible with our data: nonetheless, there are two findings that current neuropsychological computational models should try to incorporate. The first is that neglect patients show an exaggerated novelty reaction to right side visual stimuli (Lasaponara et al., 2018): this means that the reduced epistemic affordance of the left side of space is matched with a pathologically increased epistemic affordance of the right side. The second is that our study shows that the probabilistic organisation of the acoustic environment has a significant impact also on the epistemic affordance offered by stimuli in the attended right side of space. This conclusion is based on the finding that in neglect patients, these stimuli trigger no novelty P3 response when they are infrequently interspersed among frequent left side ones.

To summarise, the present study sheds new light on the poorly explored deficits of predictive coding behaviour in patients with left spatial neglect. The high time-resolution of ERPs allowed us to disclose both the pre-attentive and the attentive/belief updating components of these deficits. Taken together with recent studies that have unveiled deficits in learning and predicting the spatial position of reinforcements (Malhotra et al., 2013; Lecce et al., 2015; Li et al., 2020; Neppi-Modona et al., 2020), these findings pave new ways for interpreting the neglect syndrome and developing new rehabilitation protocols aimed at contrasting the attentional impairments suffered by these patients.

For Review Only

Acknowledgements.

The authors are grateful to all participants for involvement in the study.

Funding.

This study was supported grants from the Ministero della Ricerca Scientifica (PRIN 2017, Project Code: 2017XBJN4F) and the Universita' La Sapienza (Ricerche Ateneo, 2018) to F.D. S. L was supported by PRIN grant (Code: 2017XBJN4F) to F.D. M.Pinto was supported by a grant from the Fondazione Santa Lucia (RC20.D_F.3, 2019). M.Pellegrino and M. D'O. were supported by a grant from the Fondazione Terzo Pilastro (2020) to F.D.

For Review Only

References

Aiello, M., Jacquin-Courtois, S., Merola, S., Ottaviani, T., Tomaiuolo, F., Bueti, D., ... & Doricchi, F. (2012). No inherent left and right side in human 'mental number line': evidence from right brain damage. *Brain*, *135*(8), 2492-2505.

Aiello, M., Merola, S., & Doricchi, F. (2013). Small numbers in the right brain: evidence from patients without and with spatial neglect. *Cortex*, *49*(1), 348-351.

Albert, M. L. (1973). A simple test of visual neglect. *Neurology*.

Azouvi, P., Samuel, C., Louis-Dreyfus, A., Bernati, T., Bartolomeo, P., Beis, J. M., ... & De Montety, G. (2002). Sensitivity of clinical and behavioural tests of spatial neglect after right hemisphere stroke. *Journal of Neurology, Neurosurgery & Psychiatry*, *73*(2), 160-166.

Bartolomeo, P., Thiebaut de Schotten, M., & Doricchi, F. (2007). Left unilateral neglect as a disconnection syndrome. *Cerebral cortex*, *17*(11), 2479-2490.

Bekinschtein, T. A., Dehaene, S., Rohaut, B., Tadel, F., Cohen, L., & Naccache, L. (2009). Neural signature of the conscious processing of auditory regularities. *Proceedings of the National Academy of Sciences*, *106*(5), 1672-1677.

For Review Only

Bender, M. B., & Diamond, S. P. (1965). An analysis of auditory perceptual defects with observations on the localisation of dysfunction. *Brain*, *88*(4), 675-686.

Bozzali, M., Mastropasqua, C., Cercignani, M., Giulietti, G., Bonnì, S., Caltagirone, C., & Koch, G. (2012). Microstructural damage of the posterior corpus callosum contributes to the clinical severity of neglect. *PloS one*, *7*(10), e48079.

Colin, C., Radeau, M., Soquet, A., Dachy, B., & Deltenre, P. (2002). Electrophysiology of spatial scene analysis: the mismatch negativity (MMN) is sensitive to the ventriloquism illusion. *Clinical Neurophysiology*, *113*(4), 507-518.

Damasio, A. R., Damasio, H., & Chui, H. C. (1980). Neglect following damage to frontal lobe or basal ganglia. Neuropsychologia, 18(2), 123-132.

Dehaene, S. & Changeux, J.-P. Experimental and theoretical approaches to conscious processing. *Neuron* **70**, 200–227 (2011).

Deouell, L. Y., Bentin, S., & Soroker, N. (2000). Electrophysiological evidence for an early (pre-attentive) information processing deficit in patients with right hemisphere damage and unilateral neglect. *Brain*, *123*(2), 353-365.

de Schotten, M. T., Urbanski, M., Duffau, H., Volle, E., Lévy, R., Dubois, B., & Bartolomeo, P. (2005). Direct evidence for a parietal-frontal pathway subserving spatial awareness in humans. *Science*, *309*(5744), 2226-2228.

For Review Only

de Schotten, M. T., Tomaiuolo, F., Bartolomeo, P., & Doricchi, F. (2012). Damage to White Matter Pathways in Chronic Visuospatial Neglect (S44. 002).

Diller, L., Ben-Yishay, Y., Gerstman, L. J., Goodkin, R., Gordon, W., & Weinberg, J. (1974). Studies in scanning behavior in hemiplegia. Rehabilitation monograph no. 50. *Proceedings of the Studies in Cognition and Rehabilitation in Hemiplegia*.

Doricchi, F., & Tomaiuolo, F. (2003). The anatomy of neglect without hemianopia: a key role for parietal–frontal disconnection?. *Neuroreport*, *14*(17), 2239-2243.

Doricchi, F., Iaria, G., Silvetti, M., Figliozzi, F., & Siegler, I. (2007). The "ways" we look at dreams: evidence from unilateral spatial neglect (with an evolutionary account of dream bizarreness). *Experimental brain research*, *178*(4), 450-461.

Doricchi, F., de Schotten, M. T., Tomaiuolo, F., & Bartolomeo, P. (2008). White matter (dis) connections and gray matter (dys) functions in visual neglect: gaining insights into the brain networks of spatial awareness. *Cortex*, *44*(8), 983-995.

Ferro, J. M., Kertesz, A., & Black, S. E. (1987). Subcortical neglect: quantitation, anatomy, and recovery. Neurology, 37(9), 1487-1487.

For Review Only

Fink, G. R., Halligan, P. W., Marshall, J. C., Frith, C. D., Frackowiak, R. S. J., & Dolan, R. J. (1996). Where in the brain does visual attention select the forest and the trees?. *Nature*, *382*(6592), 626-628.

Friston, K. (2005). A theory of cortical responses. *Philosophical transactions of the Royal Society B: Biological sciences*, *360*(1456), 815-836.

Gaffan, D., & Hornak, J. (1997). Visual neglect in the monkey. Representation and disconnection. Brain: a journal of neurology, 120(9), 1647-1657.

Gratton, G., Coles, M. G., & Donchin, E. (1983). A new method for off-line removal of ocular artifact. *Electroencephalography and clinical neurophysiology*, *55*(4), 468-484.

Halligan, P. W., Marshall, J. C., & Wade, D. T. (1990). Do visual field deficits exacerbate visuo-spatial neglect?. *Journal of Neurology, Neurosurgery & Psychiatry*, *53*(6), 487-491.

Hämäläinen H, Lahtinen E, Pirilä J, Lindroos J: N1and MMN in neglect. Paper presented at MMN98, the First International Workshop on MMN and Its Clinical Applications, Helsinki, 1998

Healton, E. B., Navarro, C., Bressman, S., & Brust, J. C. (1982). Subcortical neglect. Neurology, 32(7), 776-776.

For Review Only

Husain, M. (2008). Hemispatial neglect. *Handbook of clinical neurology*, *88*, 359-372.

Jääskeläinen, I. P., Lehtokoski, A., Alho, K., Kujala, T., Pekkonen, E., Sinclair, J. D., ... & Sillanaukee, P. (1995). Low dose of ethanol suppresses mismatch negativity of auditory event-related potentials. *Alcoholism: Clinical and Experimental Research*, *19*(3), 607-610.

Karnath, H. O., Himmelbach, M., & Rorden, C. (2002). The subcortical anatomy of human spatial neglect: putamen, caudate nucleus and pulvinar. Brain, 125(2), 350-360.

King, J. R., Gramfort, A., Schurger, A., Naccache, L., & Dehaene, S. (2014). Two distinct dynamic modes subtend the detection of unexpected sounds. *PloS one*, *9*(1), e85791.

Koch, C., Massimini, M., Boly, M., & Tononi, G. (2016). Neural correlates of consciousness: progress and problems. *Nature Reviews Neuroscience*, *17*(5), 307-321.

Lasaponara, S., Dragone, A., Lecce, F., Di Russo, F., & Doricchi, F. (2015). The "serendipitous brain": low expectancy and timing uncertainty of conscious events improve awareness of unconscious ones (evidence from the attentional blink). *Cortex*, *71*, 15-33.

Lasaponara, S., D'Onofrio, M., Pinto, M., Dragone, A., Menicagli, D., Bueti, D., ... & Doricchi, F. (2018). EEG correlates of preparatory orienting, contextual updating, and inhibition of sensory processing in left spatial neglect. Journal of Neuroscience, 38(15), 3792-3808.

For Review Only

Lasaponara, S., Pinto, M., Aiello, M., Tomaiuolo, F., & Doricchi, F. (2019). The hemispheric distribution of α-band EEG activity during orienting of attention in patients with reduced awareness of the left side of space (spatial neglect). Journal of Neuroscience, 39(22), 4332-4343.

Lecce, F., Rotondaro, F., Bonni, S., Carlesimo, A., De Schotten, M. T., Tomaiuolo, F., & Doricchi, F. (2015). Cingulate neglect in humans: Disruption of contralesional reward learning in right brain damage. *Cortex*, *62*, 73-88.

Li, K., Bentley, P., Nair, A., Halse, O., Barker, G., Russell, C., ... & Malhotra, P. A. (2020). Reward sensitivity predicts dopaminergic response in spatial neglect. *Cortex*, *122*, 213-224.

Lunven, M., Thiebaut de Schotten, M., Bourlon, C., Duret, C., Migliaccio, R., Rode, G., & Bartolomeo, P. (2015). White matter lesional predictors of chronic visual neglect: a longitudinal study. *Brain*, *138*(3), 746-760.

Malhotra, P. A., Soto, D., Li, K., & Russell, C. (2013). Reward modulates spatial neglect. Journal of Neurology, Neurosurgery & Psychiatry, 84(4), 366e369.

Massironi, M., Antonucci, G., Pizzamiglio, L., Vitale, M. V., & Zoccolotti, P. (1988). The Wundt-Jastrow illusion in the study of spatial hemi-inattention. *Neuropsychologia*, *26*(1), 161-166.

For Review Only

Neppi-Mòdona M, Sirovich R, Cicerale A, et al. Following the gold trail: Reward influences on spatial exploration in neglect. *Cortex*. 2020;129:329-340. doi:10.1016/j.cortex.2020.04.027

Nijboer T, van de Port I, Schepers V, Post M, Visser-Meily A. (2013). Predicting functional outcome after stroke: the influence of neglect on basic activities in daily living. *Front Hum Neurosci* 7: 182.

Nijboer TC, Kollen BJ, Kwakkel G. (2014) The impact of recovery of visuo- spatial neglect on motor recovery of the upper paretic limb after stroke. *PLoS One*, 9: e100584.

Nyffeler, T., Vanbellingen, T., Kaufmann, B. C., Pflugshaupt, T., Bauer, D., Frey, J., ... & Cazzoli, D. (2019). Theta burst stimulation in neglect after stroke: functional outcome and response variability origins. *Brain*, *142*(4), 992-1008.

Parr, T., & Friston, K. J. (2018). The computational anatomy of visual neglect. *Cerebral Cortex*, *28*(2), 777-790.

Parr, T., Rees, G., & Friston, K. J. (2018). Computational neuropsychology and Bayesian inference. *Frontiers in human neuroscience*, *12*, 61.

Parr, T., & Friston, K. J. (2018). Active inference, novelty and neglect. In *Processes of visuospatial attention and working memory* (pp. 115-128). Springer, Cham.

Pavani, F., Husain, M., Ládavas, E., & Driver, J. (2004). Auditory deficits in visuospatial neglect patients. *Cortex*, *40*(2), 347-365.

For Review Only

Pizzamiglio L, Antonucci G, Judica A, Montenero P, Razzano C, Zoccolotti P. Cognitive rehabilitation of the hemineglect disorder in chronic patients with unilateral brain damage. J Clin Exp Neurospychol 1992; 14: 901–23.

Polich, J. (2007). Updating P3: an integrative theory of P3a and P3b. *Clinical neurophysiology*, *118*(10), 2128-2148.

Rotondaro, F., Merola, S., Aiello, M., Pinto, M., & Doricchi, F. (2015). Dissociation between line bisection and mental-number-line bisection in healthy adults. *Neuropsychologia*, *75*, 565-576.

Schneider, W., Eschmann, A., Zuccolotto, A., 2002. E-Prime v1. 1. Psychology Software Tools Inc, Pittsburgh, PA.

Sergent, C., Baillet, S., & Dehaene, S. (2005). Timing of the brain events underlying access to consciousness during the attentional blink. *Nature neuroscience*, *8*(10), 1391-1400.

Sussman, E. S. (2007). A new view on the MMN and attention debate: the role of context in processing auditory events. *Journal of Psychophysiology*, *21*(3-4), 164-175.

For Review Only

Tomaiuolo, F., Voci, L., Bresci, M., Cozza, S., Posteraro, F., Oliva, M., & Doricchi, F. (2010). Selective visual neglect in right brain damaged patients with splenial interhemispheric disconnection. Experimental brain research, 206(2), 209-217.

Vallar, G., Guariglia, C., Nico, D., & Bisiach, E. (1995). Spatial hemineglect in back space. *Brain*, *118*(2), 467-472.

Verdon, V., Schwartz, S., Lovblad, K. O., Hauert, C. A., & Vuilleumier, P. (2010). Neuroanatomy of hemispatial neglect and its functional components: a study using voxel-based lesion-symptom mapping. *Brain*, *133*(3), 880-894.

Wacongne, C., Labyt, E., van Wassenhove, V., Bekinschtein, T., Naccache, L., & Dehaene, S. (2011). Evidence for a hierarchy of predictions and prediction errors in human cortex. *Proceedings of the National Academy of Sciences*, *108*(51), 20754-20759.

Wienberg, M., Glenthoj, B. Y., Jensen, K. S., & Oranje, B. (2010). A single high dose of escitalopram increases mismatch negativity without affecting processing negativity or P3 amplitude in healthy volunteers. *Journal of Psychopharmacology*, *24*(8), 1183-1192.

Yeh, F. C., Panesar, S., Fernandes, D., Meola, A., Yoshino, M., Fernandez-Miranda, J. C., ... & Verstynen, T. (2018). Population-averaged atlas of the macroscale human structural connectome and its network topology. *NeuroImage*, *178*, 57-68.

For Review Only

Figure and table captions

Table 1. Clinical and demographic group data of RBD patients with left spatial neglect (N+), without left spatial neglect (N-), and healthy controls (HC). Maximal scores for each test are reported in parenthesis. In the acoustic task, positive scores indicate rightward deviation of the subjective auditory “straight-ahead” and negative scores leftward deviation.

Fig.1: Structure of the four types of auditory trials and the six experimental blocks/experimental-conditions used in the study

For Review Only

Fig.2: A) Probability maps of lesion overlap: First row, patients without neglect (N-); second row, patients with neglect (N+). The third row represents the peaks of lesion overlap resulting from the N+ minus N- subtraction. Note, the main peak of the subtraction (MNI coord: 27, -15, 35) is located in the superior longitudinal and arcuate fasciculi. **B)** Tridimensional reconstruction of the superior longitudinal and arcuate fasciculi, according to the atlas by Yeh et al. (2018). Red circles represent the highest (70-75%) peaks of lesion overlap resulting from the N+ minus N- subtraction.

Fig. 3: Waveforms and relative differential scalp topographies of the MMN evoked by the *left* and *right local deviants* as compared to *local standard* in HC, N- and N+. Grey shades indicate post-stimulus onset time-intervals in which a significant statistical difference is present.

Fig. 4: Waveforms and relative differential scalp topographies of the MMN component evoked by *frequent* and *infrequent local left* or *right deviant* sequences and *omissions* in HC, N- and N+. Grey shades indicate post-stimulus onset time-intervals in which a significant statistical difference is

present between *frequent* and *infrequent* trial type. Purple shades indicate post-stimulus onset time-intervals in which a significant statistical difference is present between *frequent* and *omission* trial type. Zoomed squares show the comparison with *local standard* (MMMM**M**) sequences.

Fig.5: Waveforms and relative differential scalp topographies of the P3b component evoked by *frequent* and *infrequent* stimuli presented in the left and on the right side of space in HC, N- and N+. Grey shades indicate post-stimulus onset time-intervals in which a significant statistical difference is present.

Fig.6: Waveforms and relative differential scalp topographies of the P3b component evoked in response to the *frequent* and *infrequent local left* or *right deviant* sequences and *omissions* in HC, N- and N+. Grey shades indicate post-stimulus onset time-intervals in which a significant statistical difference is present between *frequent* and *infrequent* trial type. Purple shades indicate post-stimulus onset time-intervals in which a significant statistical difference is present between *frequent* and *omission* trial type.

For Review Only

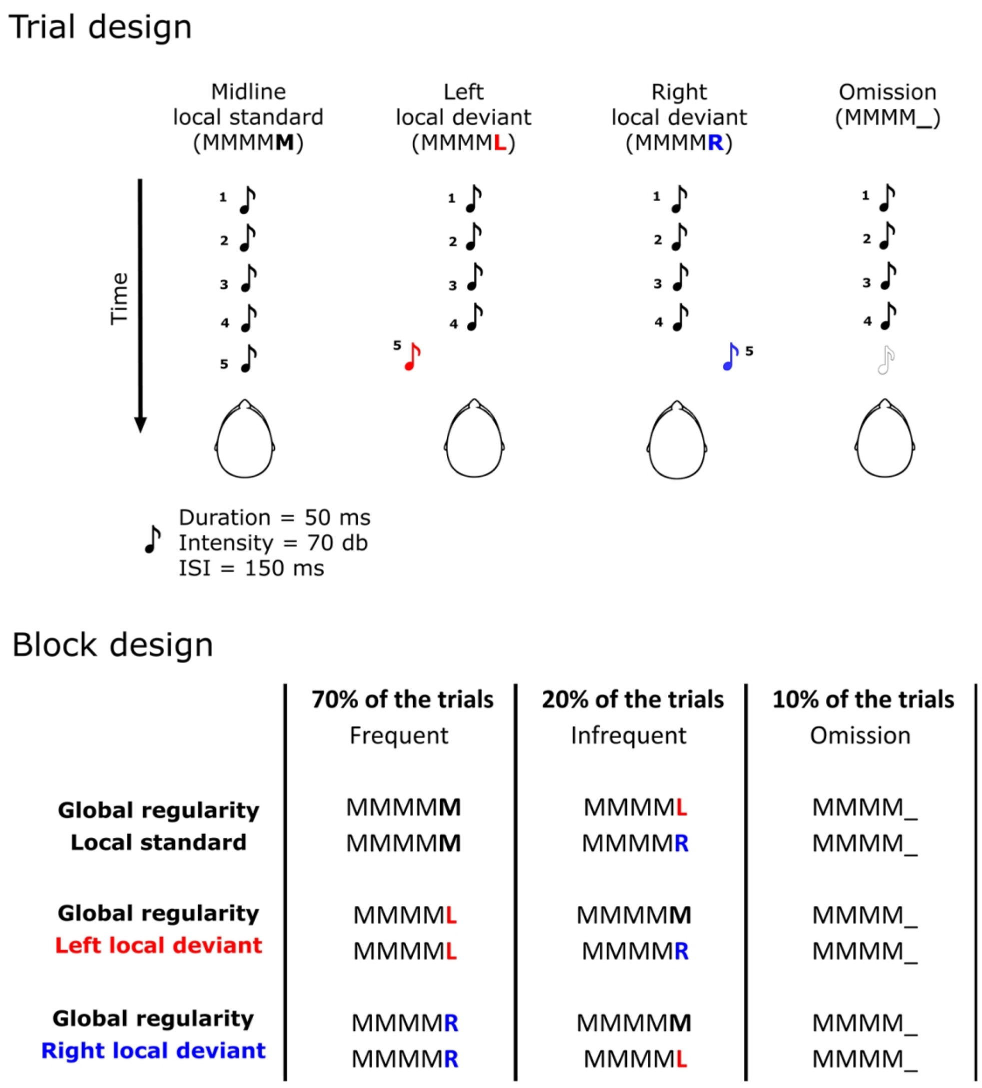

Fig.1: Structure of the four types of auditory trials and the six experimental blocks/experimental-conditions used in the study

134x150mm (300 x 300 DPI)

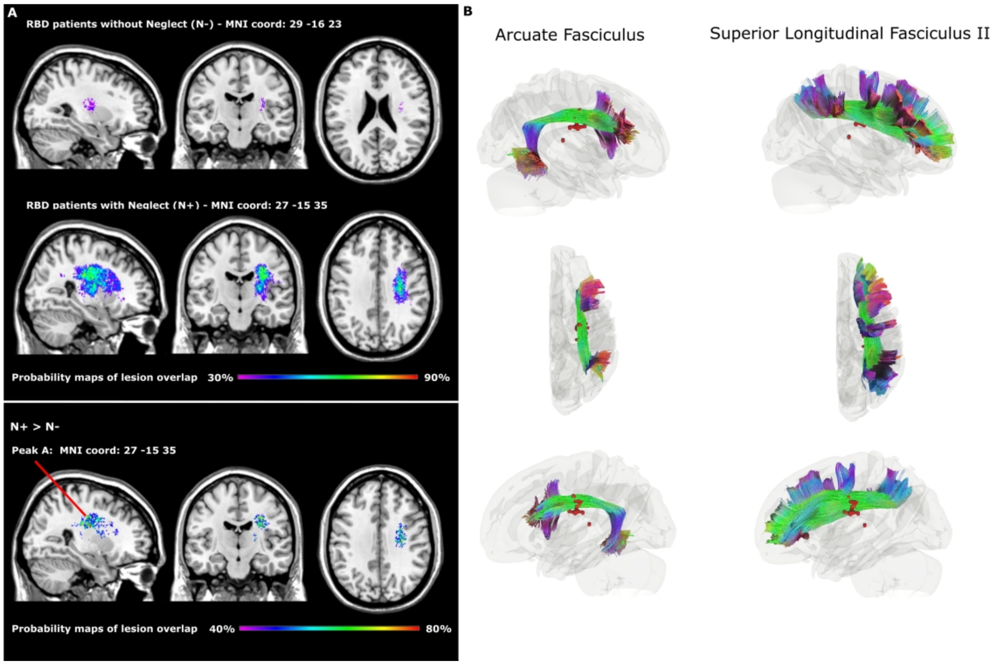

Fig.2: A) Probability maps of lesion overlap: First row, patients without neglect (N-); second row, patients with neglect (N+). The third row represents the peaks of lesion overlap resulting from the N+ minus N- subtraction. Note, the main peak of the subtraction (MNI coord: 27, -15, 35) is located in the superior longitudinal and arcuate fasciculi. B) Tridimensional reconstruction of the superior longitudinal and arcuate fasciculi, according to the atlas by Yeh et al. (2018). Red circles represent the highest (70-75%) peaks of lesion overlap resulting from the N+ minus N- subtraction.

228x150mm (300 x 300 DPI)

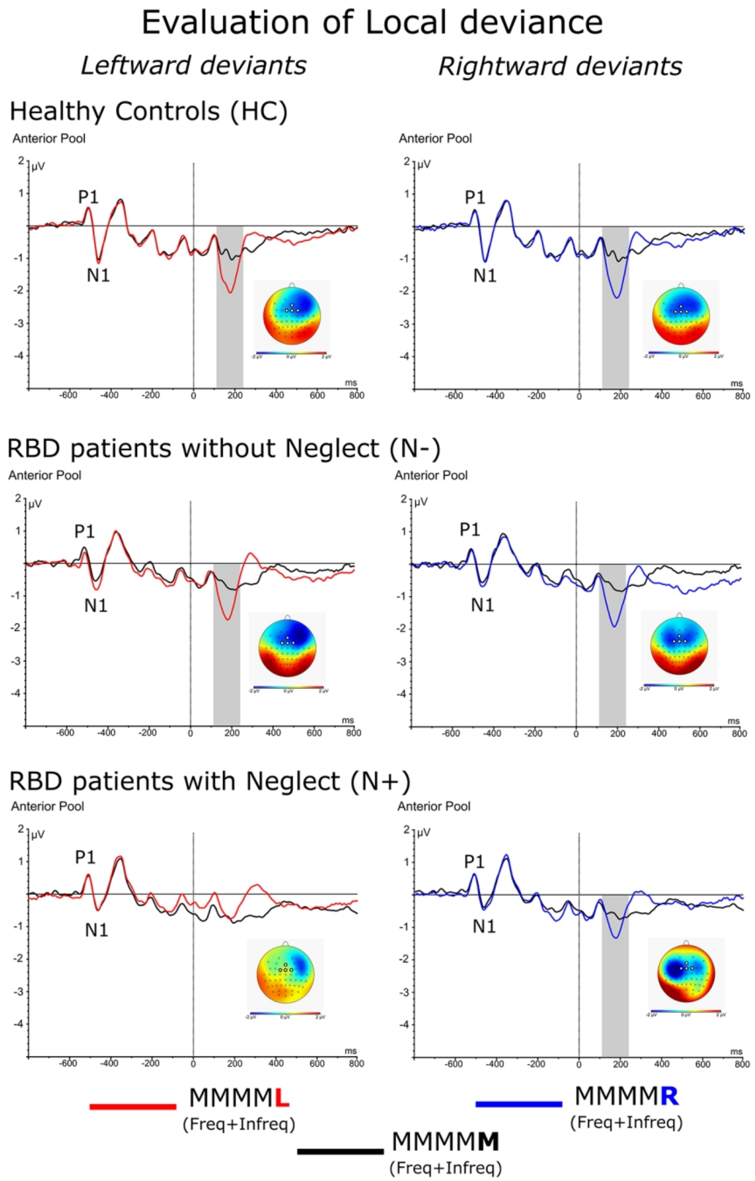

Fig. 3: Waveforms and relative differential scalp topographies of the MMN evoked by the left and right local deviants as compared to local standard in HC, N- and N+. Grey shades indicate post-stimulus onset time-intervals in which a significant statistical difference is present.

120x180mm (300 x 300 DPI)

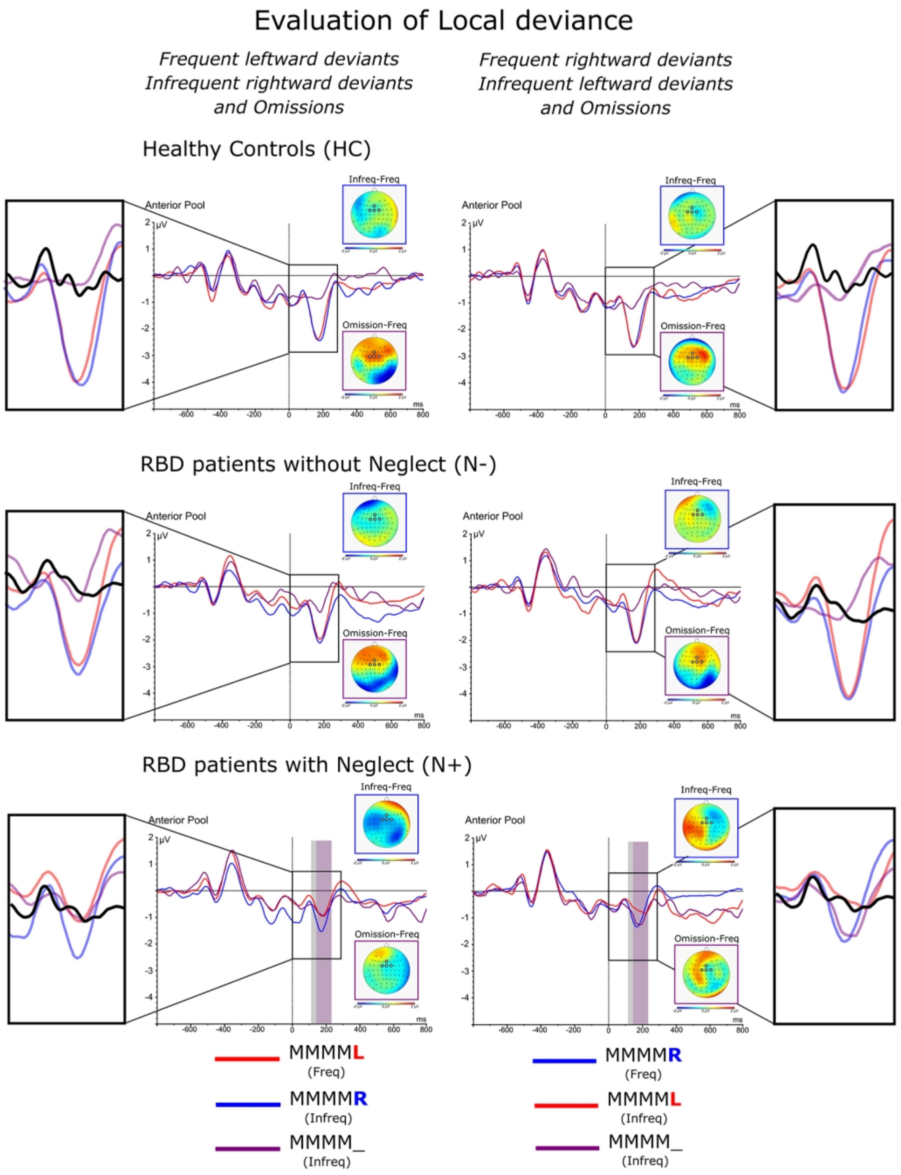

Fig. 4: Waveforms and relative differential scalp topographies of the MMN component evoked by frequent and infrequent local left or right deviant sequences and omissions in HC, N- and N+. Grey shades indicate post-stimulus onset time-intervals in which a significant statistical difference is present between frequent and infrequent trial type. Purple shades indicate post-stimulus onset time-intervals in which a significant statistical difference is present between frequent and omission trial type. Zoomed squares show the comparison with local standard (MMMMM) sequences.

143x180mm (300 x 300 DPI)

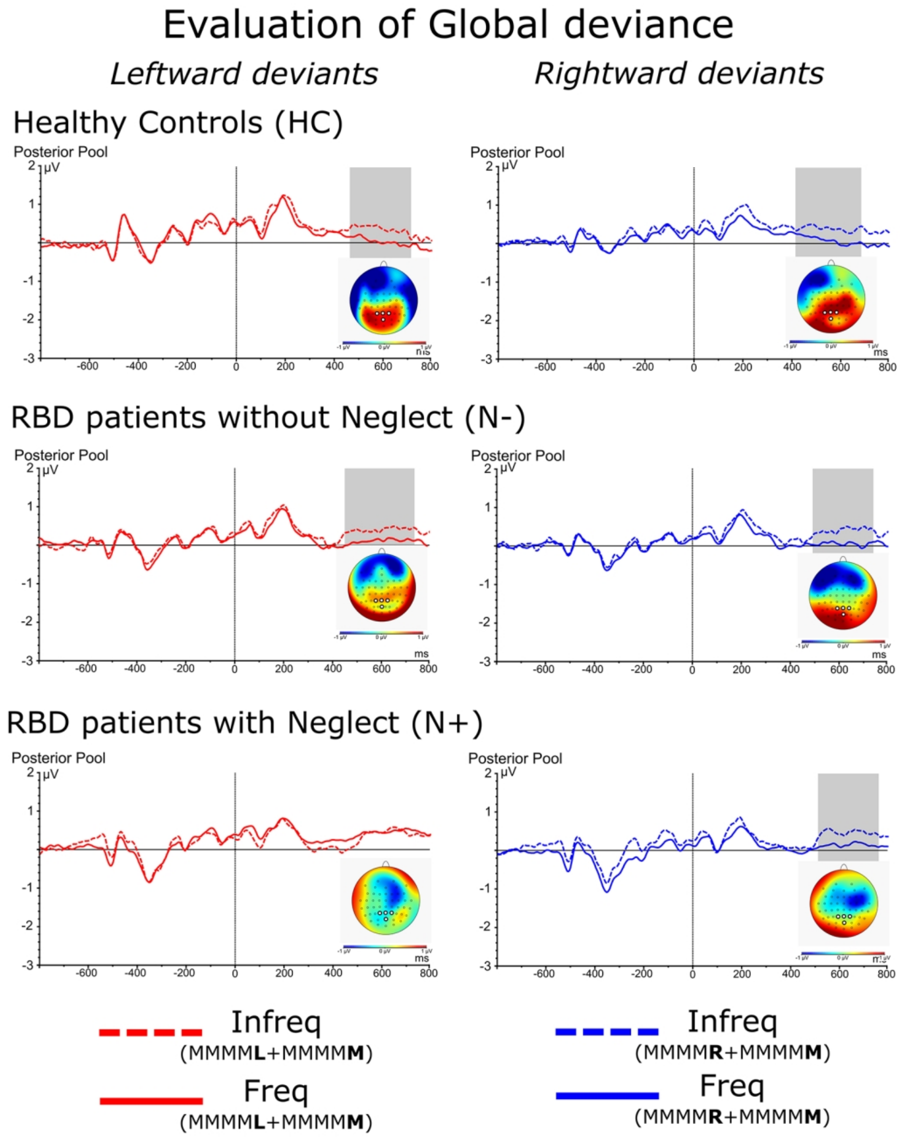

Fig.5: Waveforms and relative differential scalp topographies of the P3b component evoked by frequent and infrequent stimuli presented in the left and on the right side of space in HC, N- and N+. Grey shades indicate post-stimulus onset time-intervals in which a significant statistical difference is present.

144x180mm (300 x 300 DPI)

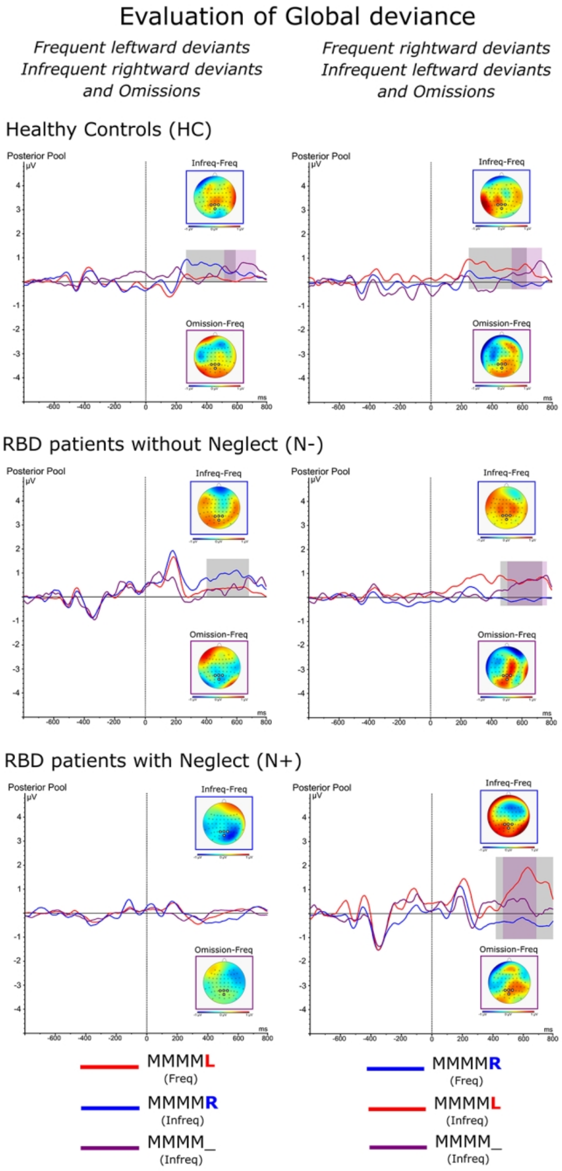

Fig.6: Waveforms and relative differential scalp topographies of the P3b component evoked in response to the frequent and infrequent local left or right deviant sequences and omissions in HC, N- and N+. Grey shades indicate post-stimulus onset time-intervals in which a significant statistical difference is present between frequent and infrequent trial type. Purple shades indicate post-stimulus onset time-intervals in which a significant statistical difference is present between frequent and omission trial type.

93x180mm (300 x 300 DPI)

Table 1. Clinical and demographic group data of RBD patients with left spatial neglect (N_), without left spatial neglect (N_), and healthy controls (HCs). Maximal scores for each test are reported in parenthesis.

Patients Group	Sex		Age (y)	Stroke onset (days)	Line bisection (200 mm) rightward deviation (mm)	Letter cancellation		Line cancellation		Star cancellation		Sentence reading test	Wundt-Jastrow illusion (unexpected responses)		Deviation of the subjective acoustic "straight ahead"
						**Left**	**Right**	**Left**	**Right**	**Left**	**Right**		**Left**	**Right**	
**N-; n = 14**															
	M=11	**Mean**	59.2	40.1	0.01	52(53)	50.4(51)	10.9(11)	9.9(10)	26.3(27)	26.8(27)	5.6(6)	1.1(20)	0(20)	1.2°
	F=3	**S.D.**	15.4	15.8	5.2	1.1	0.9	0.2	0.2	1.3	0.6	0.9	2.7	0	3.3°
**N+; n = 13**															
	M=6	**Mean**	67.6	56	19.1	29(53)	43.3(51)	8.2(11)	9.2(10)	9(27)	21.8(27)	3.5(6)	12.4(20)	1.8(20)	17.4°
	F=7	**S.D.**	11.1	27.6	20.2	17.2	14	3.4	2.1	8.8	7	2.5	6.7	2.3	20.6°
**HC; n = 16**															
	M=7	**Mean**	58.3												-0.9°
	F=9	**S.D.**	11.1												1.7°

For Review Only

Supplementary material

Deficits of hierarchical predictive coding in left spatial neglect

Fabrizio Doricchi, Mario Pinto, Michele Pellegrino, Fabio Marson, Marilena Aiello, Francesco Tomaiuolo and Stefano Lasaponara.

Clinical assessment of neglect

For Review Only

Unilateral neglect was assessed with a battery composed of six standardised tests:

(1) Line bisection (Pizzamiglio et al., 1992; Rotondaro et al., 2015): the task requires the bisection of five horizontals 200 mm lines. Each line is separately presented at the centre of a horizontally oriented A3 paper sheet. Rightward deviations from the real line centre are scored as positive deviations (in mm) and leftward deviations as negative ones. The cut-off score for spatial neglect is 6.5 mm (Azouvi et al., 2002).

(2) Letter cancellation (Diller et al., 1974): the task requires the cancellation of target capital letters presented on a horizontally oriented A3 paper sheet. Letters are arranged in six rows. In each row, target letters (H) are intermixed with filler letters (total score range 0 –104; 0 –53 on the left side, 0 –51 on the right side). The presence of neglect is diagnosed when the difference between the number of omissions in the contralesional and ipsilesional side of the sheet is higher than four.

(3) Line cancellation (Albert, 1973): the task requires the cancellation of short line segments that are arranged in scattered order and random orientation on an A3 paper sheet (total score range 0 –21; 0 –11 on the left side, 0 –10 on the right side). Neglect is diagnosed when the difference between the number of omissions the contralesional and ipsilesional side of the sheet is equal or higher than one.

(4) Star cancellation (Halligan et al., 1990): the task requires the cancellation of small stars that are presented on an A3 paper sheet interspersed with 52 large stars, 13 letters, and 10 short words that act as distracters (total score = 54: 27 on the left side and 27 on the right side). Neglect is diagnosed when the difference between the number of omissions in the contralesional and ipsilesional side of the sheet is equal or higher than three.

(5) Sentence reading test (Pizzamiglio et al., 1992): the score is the number of sentences read without omissions/errors (score range 0 – 6). One or more omissions/errors in reading the initial part of the sentence or the words composing the sentence indicates the presence left spatial neglect.

(6) Wundt–Jastrow area illusion test (Massironi et al., 1988): the score is the frequency of missed optical illusion when the two fans are oriented toward the contralesional or the ipsilesional side of space (score range 0 –20 in both cases). Neglect is diagnosed when the difference between the number of omitted illusions in the contralesional versus the ipsilesional side of space is larger than two.

Patients who failed on at least two out of the six tests were classified as suffering left spatial neglect.

For Review Only

Clinical assessment of auditory neglect

Auditory deviations from the objective midline were evaluated as follows. Blindfolded participants sat in front of a 180° graduated arch with the head position fixed and stabilised using a chin rest. The arch' endpoints (i.e., left endpoint = 0°, right endpoint = 180°) were positioned at the ear level. The centre of the arch, i.e. 90° position, was aligned to the head-body midsagittal plane (see **Supplementary Fig. 1**). The distance between the subjects' head and the arch was ~90 cm. In each trial, starting from the left or the right arch' endpoint, the experimenter slightly moved, all along the graduated arch, a buzzer producing a continuous tone of 80 dB. The participant' task was to verbally stop the sound when it reached the subjective "straight-ahead", i.e. the subjective

alignment of the tone with the head-body midsagittal plane. Five leftward and five rightward trials were alternated according to a random sequence. In each trial, scores were recorded as the difference, in degree, between the subjective and the objective "straight-ahead". Scores lower than 90° indicated rightward deviations of the "subjective straight ahead", while scores higher than 90° indicated leftward deviations.

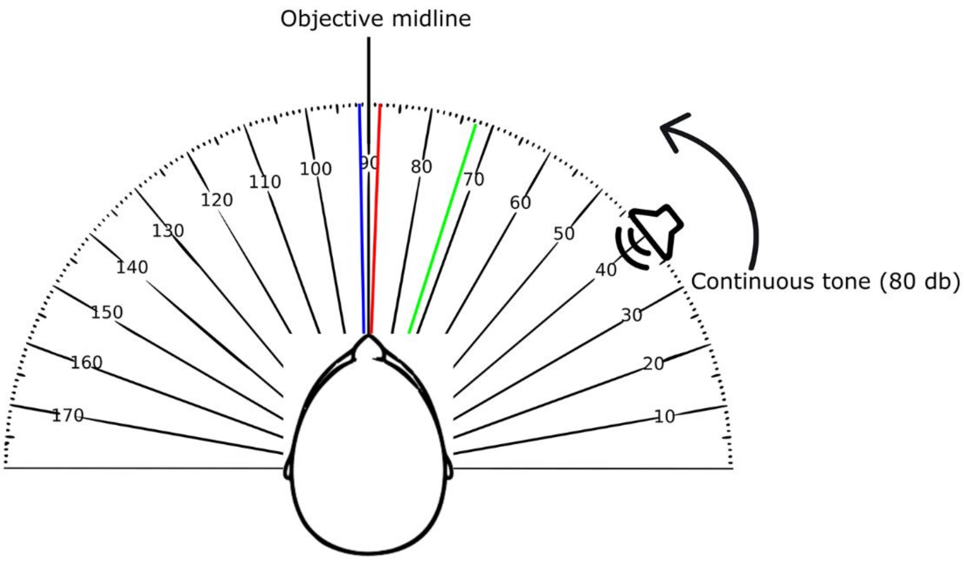

Supplementary Fig.1: Schematisation of the task used for the evaluation of auditory deviations from the objective midline. An example of a trial with a leftward start is showed in the figure. The coloured bar represents the average auditory midline measured in HC (blue), N- (red), and N+ (green).

Structure of the different six experimental block of trials.

- Blocks with Global regularity of *Local standard*:

  1) 70% *Local standard* - MMMM**M**, 20% *Left local deviant* - MMMM**L**, 10% *Omission* – MMMM_;

  2) 70% *Local standard* - MMMM**M**, 20% *Right local deviant* - MMMM**R**, 10% *Omission* – MMMM**_;**

- Blocks with Global regularity of *Left local deviant*:

  3) 70% *Left local deviant* - MMMM**L**, 20% *Local standard* – MMMM**M**, 10% *Omission* – MMMM_;

  4) 70% *Left local deviant* - MMMM**L**, 20% *Right local deviant* – MMMM**R**, 10% *Omission* – MMMM_;

- Blocks with Global regularity of *Right local deviant*:

  5) 70% *Right local deviant* - MMMM**R**, 20% *Local standard* – MMMM**M**, 10% *Omission* – MMMM_;

  6) 70% *Right local deviant* - MMMM**R**, 20% *Left local deviant* – MMMM**L**, 10% *Omission* – MMMM_;

For Review Only

Lesion mapping

Individual brain MRI or CT scan volumes were transformed into the Montreal Neurological Institute Space (2012) by means of Register (http://www.bic.mni.mcgill.ca/ServicesSoftwareVisualization/Register), an interactive graphics application that allows the simultaneous visualization of two brain volumes and the resampling of the second volume into the space and a size of the first one. In this study we used as first volume the MNI standard space brain. Twelve matched tag points were used for registering in MNI standard space brain the individual brain volumes.

Individual lesions were then drawn manually on the re-oriented normalized in MNI standard space brain using Display (http://www.bic.mni.mcgill.ca/software/Display/Display.html). This program allows labelling lesioned voxels on each slice of the MRI volume while simultaneously visualising the sagittal, axial, and coronal planes of the MRI. The labelled lesion area is then saved in binary images. The labelled voxels of each group of patients were averaged to generate the probability lesion maps. In each experimental group, the MNI coordinates of the centroids of maximal lesion overlap areas were defined using MRIcron software (http://www.mccauslandcenter.sc.edu/mricro/ mricron/). To check whether peaks of lesion overlap encroached upon white matter pathways, we used the DSI Studio software (http://dsi-studio.labsolver.org) that allows for an overlap of lesion peaks to the three-dimensional reconstruction of white matter pathways available in the diffusion tensor atlas by Yeh et al. (2018).

Criteria for selection of time windows and derivations used in the ERPs analyses

Amplitudes of the different ERPs MMN and P3 components were measured as mean activity values within corresponding component-related time windows These time windows were defined as follows. First, we individuated groups of at least 4 electrodes showing a significant deviation from the baseline on least ten consecutive 4 msec time samples (total time: 40 msec). In each 4 msec time sample the significance of deviation from the baseline was evaluated through t-test with a criterion of $p < .05$ corrected for multiple comparison. Statistically defined t-maps that highlight scalp sites where ERPs component are significantly present, allows optimizing the selection of electrodes pools. Second, for each component we established the intervals of significance using a point-by-point analysis according to Guthrie and Buchwald's criteria (see Guthrie & Buchwald, 1991).

For Review Only

Electrophysiological results

"Local" effect – MMN component

In all groups, when compared with *local standard*, no MMN was found in response to *omissions* (all F < 1, all p = n.s.; see **Supplementary Fig. 2**).

"Global" effect – P3b component

During block of trials with *frequent local standard* stimuli, *omissions* produced a significant P3b component in HC (*frequent* MMMM**M** = -0.16 µV vs. *omission* = 0.94 µV, p = 0.006), and N- (*frequent* MMMM**M** = -0.12 µV vs. *omission* = 0.68 µV, p = 0.04) though not in the N+ (Group x Frequency interaction, $F_{(2,40)} = 5.39$, $p = 0.008$, $\eta_p^2 = 0.21$; *frequent* MMMM**M** = 0.18 µV vs. *omission* = -0.5 µV, p = n.s.; **see Supplementary Fig. 2**).

For Review Only

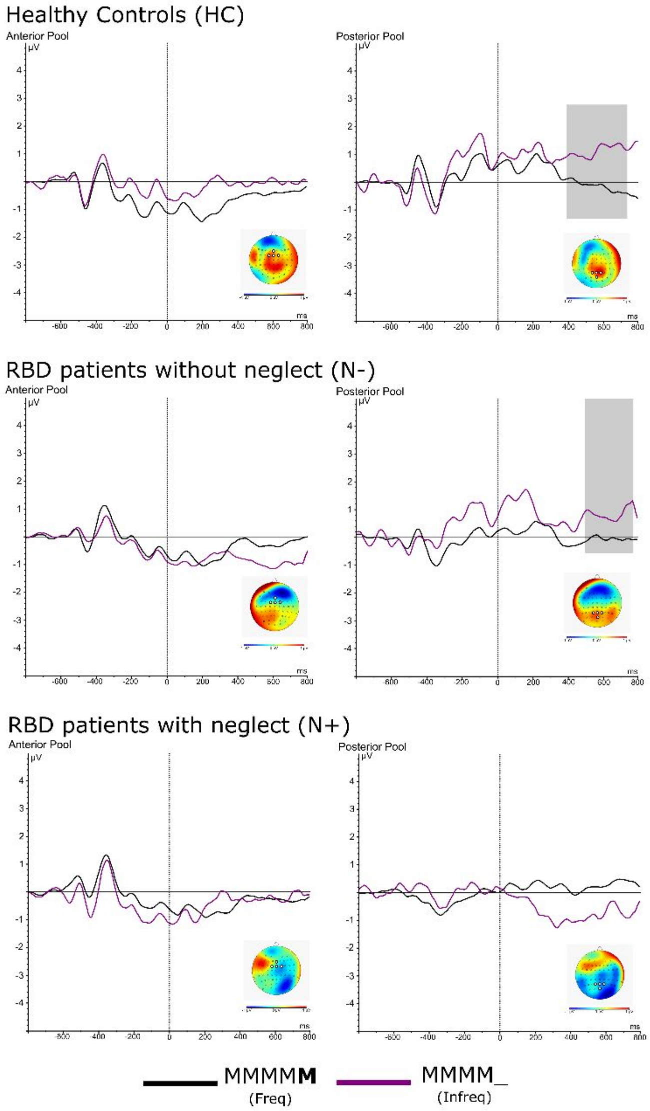

Supplementary Fig.2: Waveforms and relative differential scalp topographies of the P3b component evoked in response to *Infrequent* omission sequences as compared to *Frequent* local sequences with deviant stimuli in the left and on the right side of space and frequent local standard sequences, in HC, N- and N+. Grey shades indicate post-stimulus onset time-intervals in which a significant statistical difference is present.
