# Supplementary material for: Deficits of hierarchical predictive coding in left spatial neglect
Source: Brain Commun. 2021 Jun 4;3(2):fcab111. doi: 10.1093/braincomms/fcab111 (PMC8209285; doi:10.1093/braincomms/fcab111)
Supplement: fcab111_Supplementary_Data [file fcab111_supplementary_data.zip › Supplementary material.pdf]

Supplementary material

Deficits of hierarchical predictive coding in left spatial neglect

Fabrizio Doricchi, Mario Pinto, Michele Pellegrino, Fabio Marson, Marilena Aiello, Francesco Tomaiuolo and Stefano Lasaponara.

Clinical assessment of neglect

Unilateral neglect was assessed with a battery composed of six standardised tests:

(1) Line bisection (Pizzamiglio et al., 1992; Rotondaro et al., 2015): the task requires the bisection of five horizontals 200 mm lines. Each line is separately presented at the centre of a horizontally oriented A3 paper sheet. Rightward deviations from the real line centre are scored as positive deviations (in mm) and leftward deviations as negative ones. The cut-off score for spatial neglect is 6.5 mm (Azouvi et al., 2002).

(2) Letter cancellation (Diller et al., 1974): the task requires the cancellation of target capital letters presented on a horizontally oriented A3 paper sheet. Letters are arranged in six rows. In each row, target letters (H) are intermixed with filler letters (total score range 0 –104; 0 –53 on the left side, 0 –51 on the right side). The presence of neglect is diagnosed when the difference between the number of omissions in the contralesional and ipsilesional side of the sheet is higher than four.

(3) Line cancellation (Albert, 1973): the task requires the cancellation of short line segments that are arranged in scattered order and random orientation on an A3 paper sheet (total score range 0 –21; 0 –11 on the left side, 0 –10 on the right side). Neglect is diagnosed when the difference between the number of omissions the contralesional and ipsilesional side of the sheet is equal or higher than one.

(4) Star cancellation (Halligan et al., 1990): the task requires the cancellation of small stars that are presented on an A3 paper sheet interspersed with 52 large stars, 13 letters, and 10 short words that act as distracters (total score = 54: 27 on the left side and 27 on the right side). Neglect is diagnosed when the difference between the number of omissions in the contralesional and ipsilesional side of the sheet is equal or higher than three.

(5) Sentence reading test (Pizzamiglio et al., 1992): the score is the number of sentences read without omissions/errors (score range 0 – 6). One or more omissions/errors in reading the initial part of the sentence or the words composing the sentence indicates the presence left spatial neglect.

(6) Wundt–Jastrow area illusion test (Massironi et al., 1988): the score is the frequency of missed optical illusion when the two fans are oriented toward the contralesional or the ipsilesional side of space (score range 0 –20 in both cases). Neglect is diagnosed when the difference between the number of omitted illusions in the contralesional versus the ipsilesional side of space is larger than two.

Patients who failed on at least two out of the six tests were classified as suffering left spatial neglect.

Clinical assessment of auditory neglect

Auditory deviations from the objective midline were evaluated as follows. Blindfolded participants sat in front of a 180° graduated arch with the head position fixed and stabilised using a chin rest. The arch' endpoints (i.e., left endpoint = 0°, right endpoint = 180°) were positioned at the ear level. The centre of the arch, i.e. 90° position, was aligned to the head-body midsagittal plane (see **Supplementary Fig. 1**). The distance between the subjects' head and the arch was ~90 cm. In each trial, starting from the left or the right arch' endpoint, the experimenter slightly moved, all along the graduated arch, a buzzer producing a continuous tone of 80 dB. The participant' task was to verbally stop the sound when it reached the subjective "straight-ahead", i.e. the subjective alignment of the tone with the head-body midsagittal plane. Five leftward and five rightward trials were alternated

according to a random sequence. In each trial, scores were recorded as the difference, in degree, between the subjective and the objective "straight-ahead". Scores lower than 90° indicated rightward deviations of the "subjective straight ahead", while scores higher than 90° indicated leftward deviations.

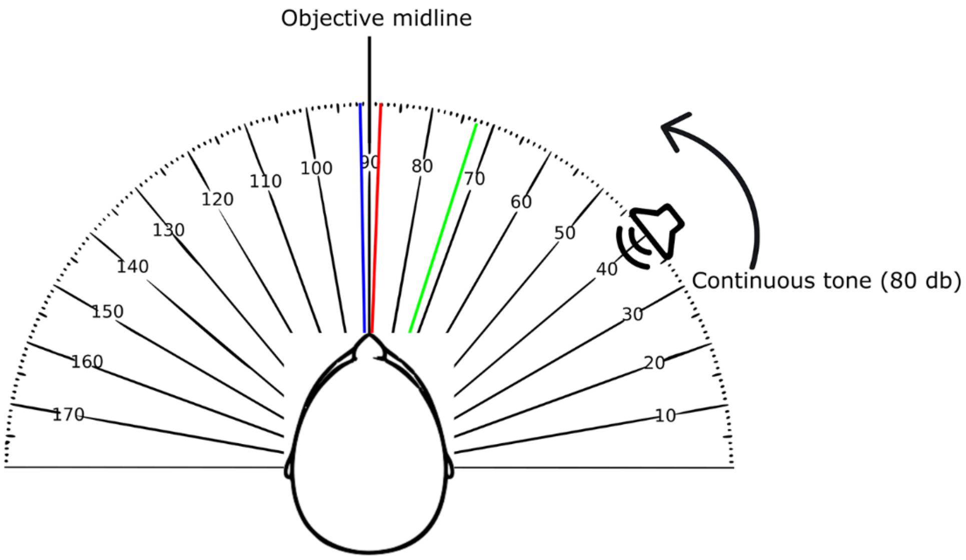

Supplementary Fig.1: Schematisation of the task used for the evaluation of auditory deviations from the objective midline. An example of a trial with a leftward start is showed in the figure. The coloured bar represents the average auditory midline measured in HC (blue), N- (red), and N+ (green).

Structure of the different six experimental block of trials.

- Blocks with Global regularity of *Local standard*:

  1) 70% *Local standard* - MMMM**M**, 20% *Left local deviant* - MMMM**L**, 10% *Omission* – MMMM**_**;

  2) 70% *Local standard* - MMMM**M**, 20% *Right local deviant* - MMMM**R**, 10% *Omission* – MMMM**_;**

- Blocks with Global regularity of *Left local deviant*:

3) 70% *Left local deviant* - MMMM**L**, 20% *Local standard* – MMMM**M**, 10% *Omission* – MMMM**_**;

4) 70% *Left local deviant* - MMMM**L**, 20% *Right local deviant* – MMMM**R**, 10% *Omission* – MMMM**_**;

- Blocks with Global regularity of *Right local deviant*:

5) 70% *Right local deviant* - MMMM**R**, 20% *Local standard* – MMMM**M**, 10% *Omission* – MMMM**_**;

6) 70% *Right local deviant* - MMMM**R**, 20% *Left local deviant* – MMMM**L**, 10% *Omission* – MMMM**_**;

Lesion mapping

Individual brain MRI or CT scan volumes were transformed into the Montreal Neurological Institute Space (2012) by means of Register (http://www.bic.mni.mcgill.ca/ServicesSoftwareVisualization/Register), an interactive graphics application that allows the simultaneous visualization of two brain volumes and the resampling of the second volume into the space and a size of the first one. In this study we used as first volume the MNI standard space brain. Twelve matched tag points were used for registering in MNI standard space brain the individual brain volumes.

Individual lesions were then drawn manually on the re-oriented normalized in MNI standard space brain using Display (http://www.bic.mni.mcgill.ca/software/Display/Display.html). This program allows labelling lesioned voxels on each slice of the MRI volume while simultaneously visualising the sagittal, axial, and coronal planes of the MRI. The labelled lesion area is then saved in binary images. The labelled voxels of each group of patients were averaged to generate the probability lesion maps. In each experimental group, the MNI coordinates of the centroids of maximal lesion overlap areas were defined using MRIcron software

(http://www.mccauslandcenter.sc.edu/mricro/ mricron/). To check whether peaks of lesion overlap encroached upon white matter pathways, we used the DSI Studio software (http://dsi-studio.labsolver.org) that allows for an overlap of lesion peaks to the three-dimensional reconstruction of white matter pathways available in the diffusion tensor atlas by Yeh et al. (2018).

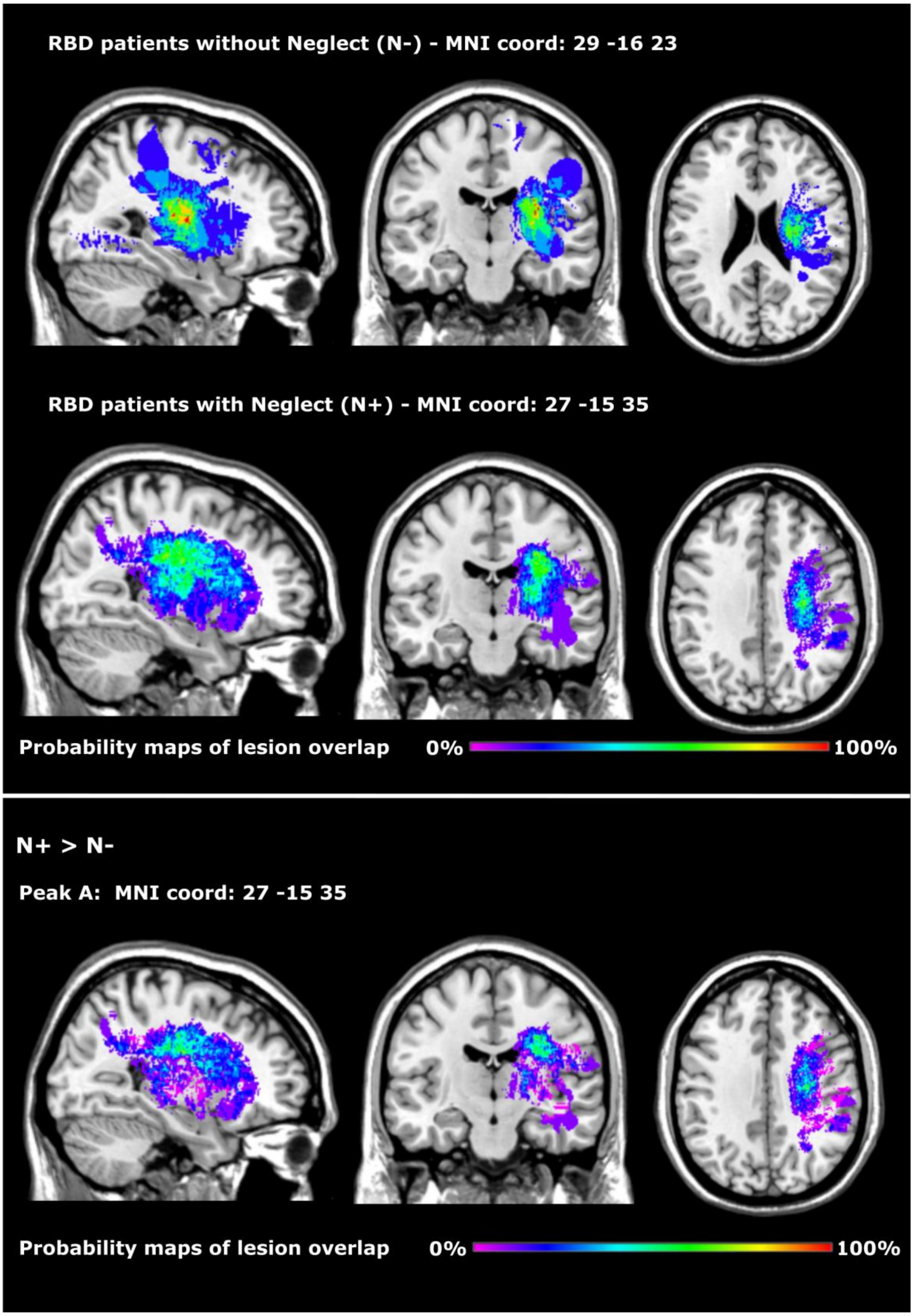

Supplementary Fig.2: Probability maps of lesion overlap: First row, patients without neglect (N-); second row, patients with neglect (N+). The third row represents the peaks of lesion overlap resulting from the N+ minus N- subtraction.

Electrophysiological analyses and results

Results of the Nonparametric FDR-corrected permutation T-tests with Monte Carlo method

Brainstorm software performs non-parametric statistical inference that does not make assumptions on the distributions of the data (Maris & Oostenveld, 2007; Pantazis et al., 2005). Permutation tests were performed across subjects for random effects inference. Under the null hypothesis of no EEG-activity difference in the sensor data between two experimental conditions “A” and “B”, in each participant the labels between conditions A and B are randomly permuted and the resulting data are used to compute a permutation t-statistic spatiotemporal sensor map. Repeating this permutation procedure 1000 times, using Monte Carlo random sampling, enables to estimate the empirical distribution of the t-statistic at each sensor and time point, and thus convert the original data into a p-value statistical map. Finally, to control for multiple comparisons across all sensors and time points, the p-values are adjusted using a false discovery rate procedure. When no statistical effect was highlighted by the permutation test, which simply means that in any of the derivation and all along the entire epochs there was no difference between the two experimental conditions involved in the comparison, for sake of homogeneity in the analysis and in the figures, we considered the pools of electrodes that for the same between-experimental conditions comparison was significant in the same group, or in the N- groups.

More specifically:

Fig.3 → In the N+ (bottom-left panel), permutation test failed to highlight a significant difference between MMMM**M** and MMMM**L** during the time windows compatible with the MMN presence (~130-200 ms). In this case, we selected the same anterior pools in which permutation test showed significant difference between the same conditions in the N- and HC (Fz – Fc1 – Fcz – Fc2)

Fig.4 → In the N+ (bottom-left panel), during the MMN time windows (~130-200 ms). permutation test highlights a significant difference between Freq MMMM**L** and Infreq MMMM**R** though in the same group, shows no significant difference between Freq MMMM**L** and Infreq Omissions. In this case, we selected the same anterior pools in which permutation test highlights the presence of a significant difference (Fc5 – Fc3 – Fc1 – C3).

Fig.5 → In the N+ (bottom-left panel), permutation test failed to highlight a significant difference between Freq and Infreq conditions during the time windows compatible with the P3b presence (~450-750 ms). In this case, we selected the same posterior pools in which permutation test showed significant difference between the same conditions in the N- (Pz – P2 – POz – PO4).

Fig.6 → In the N- (middle-left panel), during the P3b time windows (~450-750 ms). permutation test highlights a significant difference between Freq MMMM**L** and Infreq MMMM**R** though in the same group, shows no significant difference between Freq MMMM**L** and Infreq Omissions. In this case, we selected the same pools in which permutation test highlights the presence of a significant difference (P5 – P3 – PO7 – PO5). Similarly, in the N+ (bottom-left panel) permutation tests resulted as non-significant both in the Freq MMMM**L** vs. Infreq MMMM**R** and in the Freq MMMM**L** vs. Infreq Omissions comparisons. In both cases, we selected the same posterior pools in which permutation test showed significant difference between the same conditions in the N- (P5 – P3 – PO7 – PO5).

Healthy Controls (HC)		Periods of significant differential activity (ms)	Electrode pools	ERP Component
	**Local Effect Left:**<br>MMMM**L** (Freq+Infreq) > MMMM**M** (Freq+Infreq)	130 – 200	Fz – Fc1 – Fcz – Fc2	MMN
		240 – 300	Fc1 – Fcz – Fc2<br>C1 – Cz – C2	P3a
	**Local Effect Right:**<br>MMMM**R** (Freq+Infreq) > MMMM**M** (Freq+Infreq)	140 – 200	Fz – Fc1 – Fcz – Fc2	MMN
		230 – 330	Fc1 – Fcz – Fc2<br>C1 – Cz – C2	P3a
	**Global Effect Left:**<br>Infreq (MMMM**L**+MMMM**M**) > Freq (MMMM**L**+MMMM**M**)	520 – 680	Pz –PO3 – POz – PO4	P3b
	**Global Effect Right:**<br>Infreq (MMMM**R**+MMMM**M**) > Freq (MMMM**R**+MMMM**M**)	540 – 740	CPz – CP2 - Pz – P2	P3b
	**Lateralized Effect Left:**<br>MMMM**R** (Infreq) > MMMM**L** (Freq)	300 – 680	P1 – Pz – PO3 – POz	P3b
	**Lateralized Effect Left/Omissions:**<br>MMMM_ (Infreq) > MMMM**L** (Freq)	130 – 200	Fz - Fc1 – Fcz – Fc2	MMN
		540 – 780	Pz – P2 – POz – PO4	P3b
	**Lateralized Effect Right:**<br>MMMM**L** (Infreq) > MMMM**R** (Freq)	480 – 710	CP1 – CPz – C1 – Cz	P3b
	**Lateralized Effect Right/Omissions:**<br>MMMM_ (Infreq) > MMMM**R** (Freq)	140 – 200	Fz - Fc1 – Fcz – Fc2	MMN
		560 – 760	Cz – C2 – CPz – CP2	P3b

**RBD patients without neglect (N-)**				
	**Local Effect Left:**<br>MMMM**L** (Freq+Infreq) > MMMM**M** (Freq+Infreq)	140 – 220	Fz - Fc1 – Fcz – Fc2	MMN
		230 – 340	Fc1 – Fcz – Fc2<br>C1 – Cz – C2	P3a
	**Local Effect Right:**<br>MMMM**R** (Freq+Infreq) > MMMM**M** (Freq+Infreq)	140 – 220	Fz - Fc1 – Fcz – Fc2	MMN
		240 – 320	Fc1 – Fcz – Fc2<br>C1 – Cz – C2	P3a
	**Global Effect Left:**<br>Infreq (MMMM**L**+MMMM**M**) > Freq (MMMM**L**+MMMM**M**)	450 – 510/570 – 740	Pz – P2 – POz – PO4	P3b
	**Global Effect Right:**<br>Infreq (MMMM**R**+MMMM**M**) > Freq (MMMM**R**+MMMM**M**)	430 – 720	PO3 – POz – O1 – Oz	P3b
	**Lateralized Effect Left:**<br>MMMM**R** (Infreq) > MMMM**L** (Freq)	480 – 720	P5 – P3 – PO7 – PO5	P3b
	**Lateralized Effect Left/Omissions:**<br>MMMM**_** (Infreq) > MMMM**L** (Freq)	140 – 220	Fz - Fc1 – Fcz – Fc2	MMN
		-	-	-
	**Lateralized Effect Right:**<br>MMMM**L** (Infreq) > MMMM**R** (Freq)	490 - 700	Pz – P2 – POz – PO4	P3b
	**Lateralized Effect Right/Omissions:**<br>MMMM**_** (Infreq) > MMMM**R** (Freq)	160 – 220	Fz - Fc1 – Fcz – Fc2	MMN
		590 – 800	Cp2 – Cp4 – Pz – P2	P3b

RBD patients with neglect (N+)				
	**Local Effect Left:**<br>MMMM**L** (Freq+Infreq) > MMMM**M** (Freq+Infreq)	-	-	-
		230 – 340	Fc1 – Fcz – Fc2<br>C1 – Cz – C2	P3a
	**Local Effect Right:**<br>MMMM**R** (Freq+Infreq) > MMMM**M** (Freq+Infreq)	140 – 220	Fc5 – Fc3 – Fc1 – C3	MMN
		240 – 330	Fc1 – Fcz – Fc2<br>C1 – Cz – C2	P3a
	**Global Effect Left:**<br>Infreq (MMMM**L**+MMMM**M**) > Freq (MMMM**L**+MMMM**M**)	-	-	-
	**Global Effect Right:**<br>Infreq (MMMM**R**+MMMM**M**) > Freq (MMMM**R**+MMMM**M**)	420 – 690	P1 – Pz – PO3 – POz	P3b
	**Lateralized Effect Left:**<br>MMMM**R** (Infreq) > MMMM**L** (Freq)	130 – 210	Fc5 – Fc3 – Fc1 – C3	MMN
		-	-	-
	**Lateralized Effect Left/Omissions:**<br>MMMM_ (Infreq) > MMMM**L** (Freq)	-	-	-
	**Lateralized Effect Right:**<br>MMMM**L** (Infreq) > MMMM**R** (Freq)	120 – 200	Fc5 – Fc3 – Fc1 – C3	MMN
		490 – 760	Pz – P2 – POz – PO4	P3b
	**Lateralized Effect Right/Omissions:**<br>MMMM_ (Infreq) > MMMM**R** (Freq)	500 – 760	Cp2 – Cp4 – P2 – P4	P3b

Supplementary Table 1: Results of the non-parametric FDR-corrected permutation T-tests with Monte Carlo method in the three experimental groups, showing pool of derivations and time windows where a significant difference has been observed between the two experimental conditions included in the comparisons.

Primary acoustic-sensory processing

In an initial screening test, maintenance of primary acoustic sensory processing of left-side and right-side deviant tones, was verified by recording ERPs in blocks during which we presented 192 single binaural left-deviant (n. 96) and right-deviant (n. 96) tones. These tones had the same characteristics of the fifth deviant tones presented at the end of the sequences used in the main experiment, i.e. MMMM**L** and MMMM**R**. Each tones lasted 50 ms and the inter-stimulus delay between each tones was of 1150 ms. To avoid attentional effects, all left- and all right-deviant tones were separately presented in two different blocks of trials of approximately ~1.5 min duration. Participants were only asked to listen to the sounds and keep the eye on a fixation point on a monitor.

Continuous EEG recorded during these blocks was pre-processed (see Method in the main text) and successively segmented in epochs lasting 450 ms, locked to each single tones. A time period of 100 ms before this event was used for baseline-correction. Successively, the presence of reliable sensory P1 and N1 components related to the grand-average of single left-deviant and right-deviant tones was evaluated using a nonparametric permutation T-test (Sergent et al., 2005; Lasaponara et al., 2015) corrected for multiple comparisons in time and space (i.e. derivations), which contrasted voltage values against the baseline all along the entire 450 ms epoch. This analysis highlighted the presence of a positive EEG activity around 70-110 ms (P1) and of a negative activity in a time window of 120-170 ms (N1) from the onset of the tone. In a final step, to check for the presence of between-groups differences in these ERPs components, we entered individual mean amplitude from period of interests, in a series of mixed ANOVA with Group (HC, N- and N+) x Side of deviance (Left, Right) factors. Both the ANOVAs run for the P1 and N1 analysis did not show any significant main effect or interaction (all $F < 3.05$, all $p > 0.05$), suggesting the presence, in all groups, of a comparable primary sensory response to left and right deviant tones (see **Supplementary Fig. 3**).

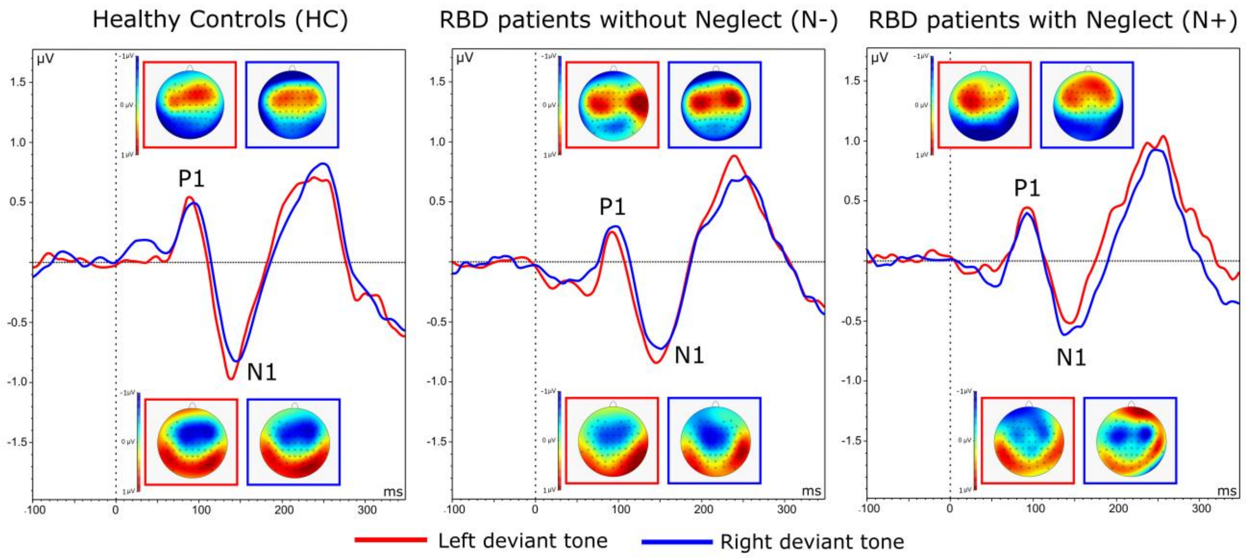

Supplementary Fig.3: Waveforms and relative scalp topographies of P1 and N1 sensory components evoked by single *left* and *right deviants* in HC, N- and N+.

*Local and Global effects related to Infrequent Omissions (MMMM_) vs. Frequent Local Standard (MMMM**M**) comparison*

In a final analysis, we compared the MMN and P3b evoked by *infrequent omissions* of the last tone (MMMM_) with that elicited by *frequent local standard* sequences (MMMM**M**). Individual data were entered in a series of Group (HC, N- and N+) x Sequence (Freq Local Standard, Infreq Omission) repeated-measures ANOVA.

"Local" effect – MMN component

In all groups, when compared with *local standard*, no MMN was found in response to *omissions* (all F < 1, all p = n.s.; see **Supplementary Fig. 4**).

"Global" effect – P3b component

During block of trials with *frequent local standard* stimuli, *omissions* produced a significant P3b component in HC (*frequent* MMMM**M** = -0.16 μV vs. *omission* = 0.94 μV, p = 0.006), and N- (*frequent* MMMM**M** = -0.12 μV vs. *omission* = 0.68 μV, p = 0.04) though not in the N+ (Group x Frequency interaction, $F_{(2,40)} = 5.39$, p = 0.008, $\eta_p^2 = 0.21$; *frequent* MMMM**M** = 0.18 μV vs. *omission* = -0.5 μV, p = n.s.; **see Supplementary Fig. 4**).

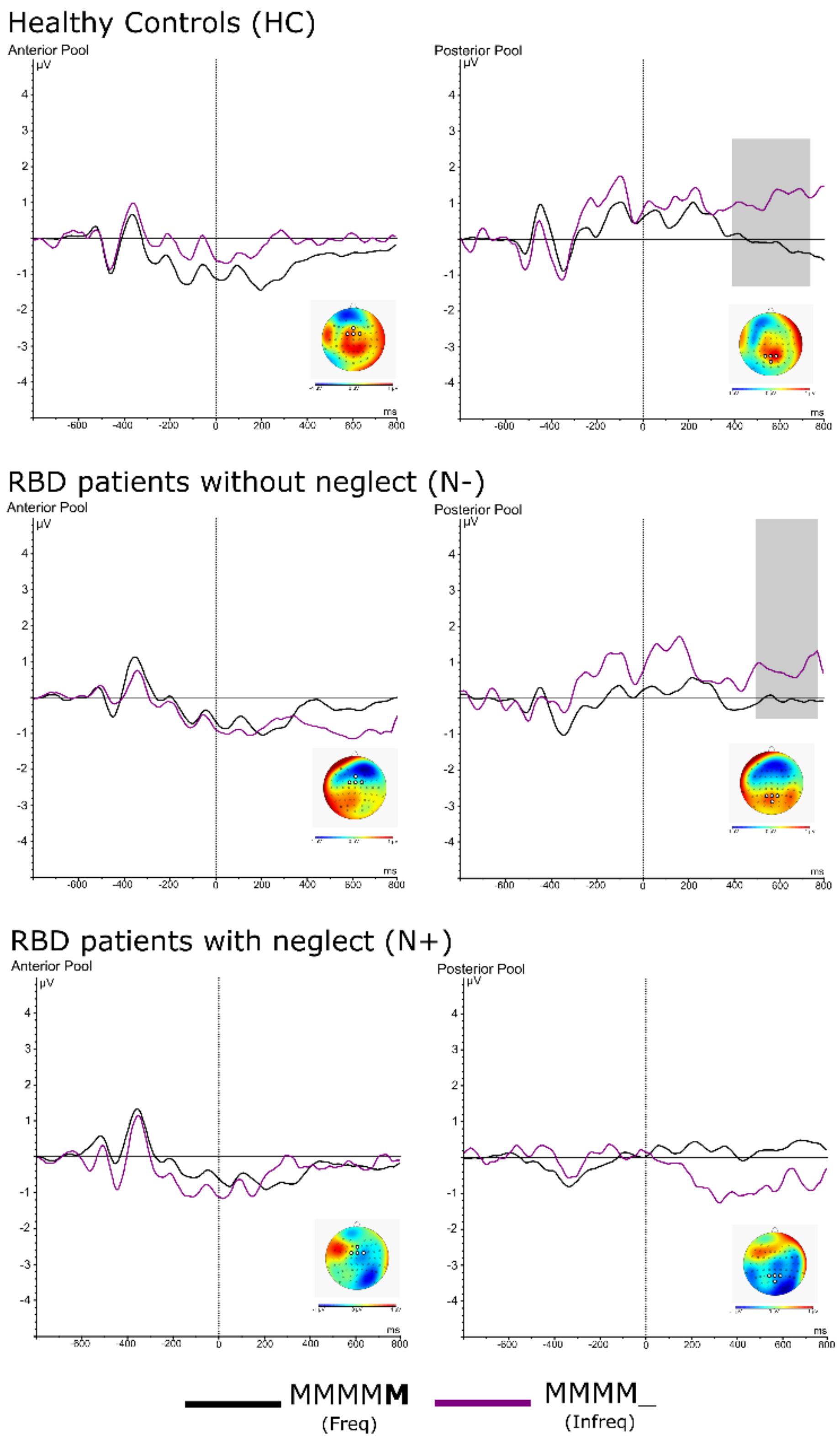

Supplementary Fig.4: Waveforms and relative differential scalp topographies of the P3b component evoked in response to *Infrequent* omission sequences as compared to *Frequent* local sequences with deviant stimuli in the left and on the right side of space and frequent local standard sequences, in HC, N- and N+. Grey shades indicate post-stimulus onset time-intervals in which a significant statistical difference is present.

References

Albert, M. L. (1973). A simple test of visual neglect. *Neurology, 23*(6), 658–664.

Azouvi, P., Samuel, C., Louis-Dreyfus, A., Bernati, T., Bartolomeo, P., Beis, J. M., ... & De Montety, G. (2002). Sensitivity of clinical and behavioural tests of spatial neglect after right hemisphere stroke. *Journal of Neurology, Neurosurgery & Psychiatry, 73*(2), 160-166.

Diller, L., Ben-Yishay, Y., Gerstman, L. J., Goodkin, R., Gordon, W., & Weinberg, J. (1974). Studies in scanning behavior in hemiplegia. Rehabilitation monograph no. 50. *Proceedings of the Studies in Cognition and Rehabilitation in Hemiplegia*.

Halligan, P. W., Marshall, J. C., & Wade, D. T. (1990). Do visual field deficits exacerbate visuo-spatial neglect?. *Journal of Neurology, Neurosurgery & Psychiatry*, *53*(6), 487-491.

Lasaponara, S., Dragone, A., Lecce, F., Di Russo, F., & Doricchi, F. (2015). The “serendipitous brain”: low expectancy and timing uncertainty of conscious events improve awareness of unconscious ones (evidence from the attentional blink). *Cortex*, *71*, 15-33.

Maris, E., & Oostenveld, R. (2007). Nonparametric statistical testing of EEG-and MEG-data. *Journal of neuroscience methods*, *164*(1), 177-190.

Massironi, M., Antonucci, G., Pizzamiglio, L., Vitale, M. V., & Zoccolotti, P. (1988). The Wundt-Jastrow illusion in the study of spatial hemi-inattention. *Neuropsychologia*, *26*(1), 161-166.

Pantazis, D., Nichols, T. E., Baillet, S., & Leahy, R. M. (2005). A comparison of random field theory and permutation methods for the statistical analysis of MEG data. *Neuroimage*, *25*(2), 383-394.

Pizzamiglio, L., Antonucci, G., Judica, A., Montenero, P., Razzano, C., & Zoccolotti, P. (1992). Cognitive rehabilitation of the hemineglect disorder in chronic patients with unilateral right brain damage. *Journal of Clinical and Experimental Neuropsychology*, *14*(6), 901-923.

Rotondaro, F., Merola, S., Aiello, M., Pinto, M., & Doricchi, F. (2015). Dissociation between line bisection and mental-number-line bisection in healthy adults. *Neuropsychologia*, 75, 565-576.

Sergent, C., Baillet, S., & Dehaene, S. (2005). Timing of the brain events underlying access to consciousness during the attentional blink. *Nature neuroscience*, *8*(10), 1391-1400.

Vallar, G., Guariglia, C., Nico, D., & Bisiach, E. (1995). Spatial hemineglect in back space. *Brain*, *118*(2), 467-472.

Yeh, F. C., Panesar, S., Fernandes, D., Meola, A., Yoshino, M., Fernandez-Miranda, J. C., ... & Verstynen, T. (2018). Population-averaged atlas of the macroscale human structural connectome and its network topology. *NeuroImage*, *178*, 57-68.
