# Supplementary material for: Deficits of hierarchical predictive coding in left spatial neglect
Source: Brain Commun. 2021 Jun 4;3(2):fcab111. doi: 10.1093/braincomms/fcab111 (PMC8209285; doi:10.1093/braincomms/fcab111)
Supplement: fcab111_Supplementary_Data [file Original Submission - Authors Response.pdf › Original Submission - Authors Response.pdf]

Point-to-point letter response to the reviewers for the Manuscript

BRAINCOM-2021-007

Deficits of hierarchical predictive coding in left spatial neglect

In red text our replies

Reviewer: 1

We thank the reviewer for appreciating our study and our efforts in data-collection. The reviewer has correctly pointed out that the method by Guthrie and Buchwald for the selection of time windows and pools for the analysis ERPs analysis, only controls for multiple comparisons across time though not across space, i.e. derivations. For this reason, we entirely re-run our EEG analyses using a series of nonparametric FDR-corrected permutation tests with Monte Carlo method. This allows highlighting pools of derivations where a significant difference between two experimental conditions is present in non pre-specified time windows. The new analyses confirmed the results that were reported in the original submission and, in addition, highlighted the presence of a new ERPs component (i.e. P3a) linked to "local" effects. Thank to this re-analysis we believe that we properly addressed all of the concerns raised by the reviewer. Please see below for a detailed point-to-point response.

Comments to the Author

The manuscript by Doricchi and colleagues presents a very interesting study, addressing a timely question – do patients with unilateral neglect suffer from a deficit in 'predictive coding' (PC), the process by which organisms use regularities to interpret their environment. PC is operationalized using sequences of tones with occasional tones deviating either locally from the repeating previous tones, or deviating globally from the probability structure of the sequence of trials. The two types of predictions are indirectly measured by the mismatch negativity (MMN) and the P3 ERP components, respectively. As noted by the authors, MMN deficits on the neglected side have been shown before in our 2000 study, but the current study is a clever extension, within the framework of modern PC terminology and moreover extending it to the global-local approach suggested more recently. The study presents a significant effort including a considerably large group of patients, with two EEG sessions for each – a challenge which cannot be underestimated. The results reported are highly interesting in my view – the MMN reduction for left side deviants is replicated in the new design (in the 2000 paper both standards and deviants were lateralized, whereas here the standards are central) and intriguingly the extraction of the global deviation depends on the side where the more frequent local deviations occur. If the reported results are upheld (see my concerns below), it might indicate a deficit in using information from the neglected side to form a model of the environment, including of the non-neglected side.

Below I list several methodological concerns that make it difficult for me to evaluate the results.

1. The results show little if any MMN elicited by left side deviants in N+ patients. This

nicely replicates our previous results which is satisfying of course. One concern that readers could have is that regardless of predictive coding or prediction errors, the N+ patients may have a basic deficit in responding to left side stimuli (from a hearing impairment to having lesions along the auditory pathway) and that is why we get a diminished MMN. Indeed, the authors must be aware of this, and thus in the beginning of the introduction they state that '…patients with neglect showed intact primary sensory processing of both left-side and right-side deviant tones, as demonstrated by the comparable latency and amplitude of the P1 and N1 responses…". However, I couldn't find this point in the results (sorry if I missed it) and in fact I am not sure how this would be measured in this design, independent of the deviance response (because lateral tones never served as local standards).

The reviewer is simply and entirely right. While preparing the initial submission and re-working intensively on an initial draft of the manuscript to reduce its length according to submission guidelines, we removed from the main text the results of a preliminary screening EEG examination in which, in different blocks of trials, we presented only left-deviant of right deviant tones. At the same time we just entirely failed, as originally planned, to re-include this part of the text in the supplementary material. This is why the reviewer could not find this set of data, which is now included in the supplementary file (an appropriate re-direction to supplementary data is signalled in the method section and in the corresponding discussion paragraph).

2. Looking at the critical figure 6, bottom row, there is a glaring difference for the N+ group in the PRE-deviant response between the condition of frequent left deviants (left bottom panel) and the condition with frequent right deviants (right bottom panel), with a strong "N1" response (circa -450ms) on the right panel but not on the left panel. This is odd because the 4 pre-deviant stimuli are the same for all trials and blocks, so why should there be any difference at this time? Then, looking at the N- patients, there is again a major difference between the right and left panels, now in the opposite direction. To cut a long story short, could it be that the plots on the right column are swapped between the two patient groups?

No, original plots were not swapped; we have now carefully checked our waveforms and excluded this possibility. Nonetheless, please note that the re-analysis of our data led to a change in the pools selected for evaluating Lateralized Global effects reported in Fig. 6: if one looks at new figure 6, it is evident that no difference in pre-deviant response is present. We presume that previous differences were due to the inclusion of electrodes/derivations where the voltage was low or noisy. The new permutation analyses that are based on the reviewer's suggestion probably optimised the selection of electrodes to be included in the pools.

3. Concerning pre-deviant responses, I am also not clear on why there should be such a big difference in the shape of the waveforms in the pre-deviant time between figures 5 and 6. Indeed, in fig. 5 local standards are collapsed with local deviants (e.g. MMMMM + MMMML) and in fig. 6 this is not the case. But again, pre-deviant there couldn't be a real difference so MMMMM+MMMML should be quite similar to MMMML except for higher SNR in the former due to having more trials. Was a different filter applied maybe?

No, there was no different filter applied. The cause of the difference highlighted by the reviewer depends on the fact that both in the previous and new version of Fig 5, Infrequent MMMML waveforms include all infrequent MMMML sequences that were presented in the experiment, i.e. those included in blocks with predominant MMMM**M** together with those included in blocks with predominant MMMM**R.** In contrast to this, in Fig. 6 the Infrequent MMMM**L** are only taken from the block with predominant MMMM**R.** In the revised manuscript we have better specified this point, that in the original version was only deducible basing on the description of statistical analyses (see page 12, line 38-44 and page 13, line 3-13 in the original submission).

4. In fig. 5 the critical result is the lack of positive difference (P3) between infrequent and frequent sequences for N+ patients, when deviance is on the left (left bottom panel). However, it seems that the outstanding condition is the frequent (global standard) rather than the infrequent (left global deviant) response. The infrequent response displays a positivity starting at ~450ms as in all other groups and conditions. It is the frequent condition (global standard, MMMML+MMMMM) which is unusually positive here. This makes it difficult to conclude that there is no P3 to the infrequent global deviant in this condition.

Looking at the results of the new analyses, it is clear that in the N+ group, frequent left-side and right-side stimuli evoke comparable positive activity within the P3b time period. In contrast to this, during the same time period only right-side infrequent stimuli produce a significant increase of positive activity compared to frequent stimuli. Also in this case, re-running the ERPs analyses according to the suggestions of the reviewer was useful. Having said this, we would like to note that within the experimental paradigm used in our study (see Bekinschtein et al., 2009 and Wacongne et al., 2011), the P3 is defined as a differential ERP component, that emerges from the comparison between Global Deviants vs. Global Standards stimuli, and that, notwithstanding the point made by the reviewer, a significant differential amplitude of the P3 was also present in the original analyses.

5. The finding regarding the omissions (discussed on page 23, lines 32-55) is super interesting in my opinion. The implications are mind boggling – it suggests that the system has already learned (has a prior) the abnormal responses elicited by left side stimuli! What is worrying though is that we do not see comparable omission responses in the N- or HC groups. Can this be explained?

Yes, as N- and HC have no pathological lateral bias of spatial attention. The presence, in the N+ group, of the MMN in response to omissions when in a block of trials right-deviants were predominant, can be linked to pathological hyper-attention to regular/frequent events in the ipsilesional side of space and the consequent load/role that this events have in shaping predictions. Because of this, omissions were elaborated as a specific form of deviant tones from highly expected right-side deviants. Viceversa, since no predictions were elaborated on the basis of frequent left-side tones, in this latter case omissions did not reach the status of unexpected events. This reasoning implies that the global distribution of events had some influence on the MMN, which is in agreement with similar results by Wacongne et al. (2011), as already commented in the original manuscript (see page 24 in the revised version "*These results show that the pre-attentive processing of the*

same sensory omissions changed utterly depending on the predominant presentation of tones in the left or right side of space, thus exemplifying the profound effects that the probabilistic sensory context can exert on the behaviour of neglect patients (see Wacongne et al., 2011)".

Otherwise it makes one wonder about the omission response seen in the N+ patients in the frequent MMMMR condition.

Please note that average response to omissions in the Frequent-Left side deviant condition and in the Frequent-Right side deviant one, is gathered from the same patients and from an identical number of trials. Put in other words, we think that for this reason the main sources of variance were under control and that it is probably unlikely that any difference and the presence of the MMN to omitted tones in the Frequent-Right-side deviant condition arose from uncontrolled variance.

6. Patients: It is not clear what were the criteria for including patients in the N+ group. Did they have to show abnormal results in all 6 tests or just in some?

As reported in the original version of our manuscript (page 6, line 43-46): "*Patients who failed on at least two out of the six tests were classified as suffering left spatial neglect*."

7. Lesions: The lesion overlap figure shows overlap only in white matter. It would be a very selective group if there were no damage to the cortex in any patient. I guess it looks like this because the scale starts with 30% overlap. It would be more informative to start from 0 overlap to show the entire extent of the lesions. Also, it should be noted that the lesions of the N- patients must have been much smaller in volume, considering the negligible overlap.

A figure of lesions overlap with a scale ranging from 0 to 100%, thus allowing complete visualization of lesion overlap in cortical areas, has been now included in the supplementary material (see **Supplementary Fig. 2**).

8. Did the MRI normalization procedure take into account the lesioned tissue (see Brett et al., 2001)? Please also report who delineated the lesion on the MRI.

Yes, lesioned tissue was included in the normalization, however we did not used an automatic computerized normalization algorithm but rather a manual normalization process based on twelve anatomical landmarks (software REGISTER (http://www.bic.mni.mcgill.ca/ServicesSoftwareVisualization/Register): this point was already specified in the original Supplementary file. An expert anatomist, Prof. Francesco Tomaiuolo, who was blind to the purpose of the study and the identity of patients, normalised MRI scans and mapped brain lesions. This is now better specified in the method section of the revised manuscript**.**

9. I was confused about the procedure used to select the window of analysis (last paragraph of p. 11). If I correctly understood, the dependent variable in the main analysis of the MMN was the average amplitude in a fixed window of 170-220ms which was determined by performing a time resolved t-test with the Guthrie and Buchwald method for

correction of multiple comparisons (this is described in the supplementary but should be described in the main text in my opinion). By my reading, the t contrast was between every point and the baseline. Was this done for every possible condition ({MMMMM, MMMMR, MMMML, MMMM_} X {frequent, infrequent} * {N+, N-, HC})? If so, how were the results combined to form a fixed ROI\window for the MMN across conditions? If not, what was the procedure exactly? This is essential to clarify to make sure the procedure doesn't bias (double-dipping) the main analysis. Finally, in the figures there are shadings for time windows of significant difference between conditions. Are these also based on the Guthrie and Buchwald method, and if so was there any control for multiple comparisons across electrodes (the method controls only across time) and how was 10 samples determined as the cutoff for significance?

As anticipated in the introduction of our replies, based on this point, we entirely re-analysed and re-tested our original findings through a series of non-parametric FDR-corrected permutation analyses with Monte Carlo method.

In the original manuscript, for sake of brevity we only reported a fixed pool/time window for the MMN and another fixed pool/time window for the P3. As far as those time window are concerned, in the original manuscript we omitted to specify that both windows were identified by choosing the time-period that common to all experimental conditions and all experimental groups. The determination of the 10-sample cutoff was based on previous works, see for example:

Sergent, C., Baillet, S., & Dehaene, S. (2005). Timing of the brain events underlying access to consciousness during the attentional blink. *Nature neuroscience*, *8*(10), 1391-1400.

Bekinschtein, T. A., Dehaene, S., Rohaut, B., Tadel, F., Cohen, L., & Naccache, L. (2009). Neural signature of the conscious processing of auditory regularities. *Proceedings of the National Academy of Sciences*, *106*(5), 1672-1677.

Minor comments

1. Add error measures around the waveforms if possible.

We would like to kindly ask the reviewer to avoid this point because our analysis software (Analyzer 2 – BrainProduct) does not allow an automatic application of standard errors/interval of confidence bars. This means that a time consuming manual export of all data is required to transfer them to another software allowing this step. Statistical indexes demonstrating the strength of our data are already reported in the manuscript.

2. Please add information about the stimuli (frequency content, envelope)

Thanks for noting this: in the revision we have added a complete description of sound stimuli used in our study.

3. Was the patient's pure tone hearing level tested?

No: in an initial screening we examined ERPs evoked by left and right deviant tones and found no side related difference in primary P1 and N1 responses (see our reply to point 1).

4. Information is missing about the order of the blocks and counterbalancing

Right: this information is now reported in the revision

5. The text says the SOA was 150 ms, while figure 1 says the ISI was 150ms. Looking at the ERP figures, it must be that the SOA was 150ms and the ISI was 100ms. But, the methods section also says that a trial lasted 850 ms – which is more consistent with ISI of 150 (and therefore SOA of 200ms). Please clarify, perhaps with adding the intervals in Figure 1?

In the original manuscript we erroneously used the term "SOA" instead of "ISI". This has been corrected in the revision

6. The methods section only mentions an online filter of .01-60Hz. Was any filtering done during pre-processing of the data (the waveforms look very smooth for a 60 Hz low pass)?

Yes, an offline 30 Hz lowpass filter was applied. We added this information in the revised manuscript

7. Page 3 last line – Deouell et al. presented a continuous sequence of tones with interspersed standards and deviants. The 'four' is redundant in this sentence.

Corrected

8. I suggest being a bit less conclusive about the underlying processes of ERP components (e.g. what the P3b marks, p. 4 line 41). We don't really know.

We smoothed the tone of the statement by saying that (page 5): *"Since the P3b probably marks the processing …"*

9. P. 8 line 6 – I suggest replacing 'blocked' by 'supported'.

Corrected

Leon Deouell

Reviewer: 2

Comments to the Author
I am grateful for the opportunity to review this paper, in which the authors use a mismatch negativity paradigm, equipped with global in addition to local deviations, to investigate spatial neglect.

The design of the experiment is attractive in its simplicity, and in the clever use of omissions as a form of non-spatial deviant tone, contrasting with the left and right local deviants. The controls seem appropriate (both healthy controls, and patients with right brain lesions without neglect). I found the experiments and the discussion of the results convincing.

Many thanks for appreciating our work.

There were only two things I thought were missing. The first is reference to the excellent work by Martin Dietz and colleagues on this problem. For example https://doi.org/10.1523/JNEUROSCI.3765-13.2014, https://doi.org/10.1016/j.heares.2020.108052. These use a similar auditory paradigm in neglect patients, and apply dynamic causal modelling to interpret EEG responses in terms of effective disconnections. It strikes me that this is highly relevant to the authors' work and could be seen as offering a mechanistic physiological account of how the results in this study were generated..I would suggest that the authors relate their results back to these papers in the discussion, and that they rephrase their sentence from the introduction '...deficits of predictive coding in patients with neglect have never been directly investigated...' to highlight that there are a few studies that do this.

Thanks for signalling these papers that are now quoted and summarised in the revised Intro section. Please note that in the original submission when talking about studies of predictive coding in patients with neglect we already specified that: "*…a few studies offer insights on this issue*.

We are happy to quote these studies though, for sake of reciprocity, we noted that none of these studies quotes our 2003 work (Doricchi, F., & Tomaiuolo, F. (2003). The anatomy of neglect without hemianopia: a key role for parietal–frontal disconnection?. *Neuroreport*, *14*(17), 2239-2243) which was the first study that provided evidence for a parietal-frontal disconnection hypothesis of neglect. As an example, in the discussion section Dietz et al. 2020) just say that "*In keeping with the dysconnection hypothesis for neglect (Corbetta and Shulman, 2011; Dietz et al., 2014; He et al., 2007), we specifically show that neglect patients present with a dysconnection between parietal, frontal, and temporal regions in the right hemisphere* "

The second thing I would suggest including is a sentence in the introduction to highlight that, by predictive coding, the authors are referring to predictive processing more broadly. The reason for this is that some of the computational papers the authors refer to are based upon discrete partially observed Markov decision processes, as opposed to the Kalman filter style models associated with predictive coding. The underlying maths is very similar,

and the authors' results are equally applicable to both. The term 'predictive processing' is sometimes used as a broader term that encompasses both styles of modelling.

At the end of the first paragraph of the Intro section we have specified that “*Here, by “predictive coding” we shall refer more broadly to “predictive processing” which encompasses both computational models based upon discrete partially observed Markov decision processes (Friston & Kiebel, 2009) and Kalman filter style models (Silvetti et al. 2018)*.”

Finally, there is a typo on page 19 'frequent lef-side' (should be 'left').

Corrected
