# Supplementary material for: Deficits of hierarchical predictive coding in left spatial neglect
Source: Brain Commun. 2021 Jun 4;3(2):fcab111. doi: 10.1093/braincomms/fcab111 (PMC8209285; doi:10.1093/braincomms/fcab111)
Supplement: fcab111_Supplementary_Data [file Original Submission – Decision Letter.pdf › Original Submission – Decision Letter.pdf]

Original Submission – Decision Letter

10-Feb-2021

Dear Dr. Doricchi,

Thank you for your recent submission BRAINCOM-2021-007
entitled "Deficits of hierarchical predictive coding in left spatial neglect" to Brain Communications.

Your manuscript has been reviewed, and while both reviewers were very positive, they have some constructive critiques that I think you could address in a revision of the manuscript. Therefore, I invite you to respond to the reviewers' comments and revise your manuscript. The reviewers' comments are included at the bottom of this message.

In addition to the reviewers' comments, please note our editorial comments added below to comply with our requirements for presentation of scientific data at Brain Communications.

Please format your revised manuscript according to our Instructions to Authors https://academic.oup.com/braincomms/pages/General_Instructions – a summary of key points is attached to help with this.

To revise your manuscript, click the link below or log into https://mc.manuscriptcentral.com/braincom and enter your Author Centre, where you will find your manuscript title listed under "Manuscripts with Decisions." Under "Actions," click on "Create a Revision." Your manuscript number has been appended to denote a revision.

*** PLEASE NOTE: This is a two-step process. After clicking on the link, you will be directed to a webpage to confirm. ***

https://mc.manuscriptcentral.com/braincom?URL_MASK=70d8c0652cfe4e41861bead05251e0e1

Please upload a revised manuscript with the changes you have made shown either with "track changes" or in coloured text.

When submitting your revised manuscript, you will be asked to respond to the reviewer(s) comments. Please be as specific as possible in your response to all of the comments.

The editorial team at Brain Communications also aims to increase transparency in scientific publishing and to demonstrate the value that peer review brings to the scientific process. Please therefore consider whether you are willing to have reviewer comments on your paper and your response published as supplementary data with the paper if it is accepted. This is entirely optional and will only be done if both the authors and reviewers agree. When submitting your revision you will have the opportunity to update the answer you entered previously.

IMPORTANT: Your original files are available to you when you upload your revised manuscript. Please delete any redundant files before completing the submission.

Because we are trying to facilitate timely publication of manuscripts submitted to Brain Communications, we would appreciate your revised manuscript as soon as possible. If it is not possible for you to resubmit within 3 months, the option to submit a revision will expire, and we would then consider your revised paper as a new submission. However, if you need a little more time, please get in touch and we can extend this deadline for you.

Once again, thank you for submitting your manuscript to Brain Communications and I look forward to receiving your revision.

Yours sincerely,
Prof. Tara Spires-Jones
Editor, Brain Communications

Reviewer(s)' Comments to Author:

Reviewer: 1 (Leon Deouell)

Comments to the Author

The manuscript by Doricchi and colleagues presents a very interesting study, addressing a timely question – do patients with unilateral neglect suffer from a deficit in 'predictive coding' (PC), the process by which organisms use regularities to interpret their environment. PC is operationalized using sequences of tones with occasional tones deviating either locally from the repeating previous tones, or deviating globally from the probability structure of the sequence of trials. The two types of predictions are indirectly measured by the mismatch negativity (MMN) and the P3 ERP components, respectively . As noted by the authors, MMN deficits on the neglected side have been shown before in our 2000 study, but the current study is a clever extension, within the framework of modern PC terminology and moreover extending it to the global-local approach suggested more recently. The study presents a significant effort including a considerably large group of patients, with two EEG sessions for each – a challenge which cannot be underestimated. The results reported are highly interesting in my view – the MMN reduction for left side deviants is replicated in the new design (in the 2000 paper both standards and deviants were lateralized, whereas here the standards are central) and intriguingly the extraction of the global deviation depends on the side where the more frequent local deviations occur. If the reported results are upheld (see my concerns below), it might indicate a deficit in using information from the neglected side to form a model of the environment, including of the non-neglected side.

Below I list several methodological concerns that make it difficult for me to evaluate the results.

1. The results show little if any MMN elicited by left side deviants in N+ patients. This nicely replicates our previous results which is satisfying of course. One concern that readers could have is that regardless of predictive coding or prediction errors, the N+ patients may have a basic deficit in responding to left side stimuli (from a hearing impairment to having lesions along the auditory pathway) and that is why we get a diminished MMN. Indeed the authors must be aware of this, and thus in the beginning of the introduction they state that '…patients with neglect showed intact primary sensory processing of both left-side and right-side deviant tones, as demonstrated by the comparable latency and amplitude of the P1 and N1 responses…". However I couldn't find this point in the results (sorry if I missed it) and in fact I am not sure how this would me measured in this design, independent of the deviance response (because lateral tones never served as local standards).
2. Looking at the critical figure 6, bottom row, there is a glaring difference for the N+ group in the PRE-deviant response between the condition of frequent left deviants (left bottom panel) and the condition with frequent right deviants (right bottom panel), with a strong "N1" response (circa -450ms) on the right panel but not on the left panel. This is odd because the 4 pre-deviant stimuli are the same for all trials and blocks, so why should there be any difference at this time? Then, looking at the N- patients, there is again a major difference between the right and left panels, now in the opposite direction. To cut a long story short, could it be that the plots on the right column are swapped between the two patient groups?
3. Concerning pre-deviant responses, I am also not clear on why there should be such a big difference in the shape of the waveforms in the pre-deviant time between figures 5 and 6. Indeed, in fig. 5 local standards are collapsed with local deviants (e.g. MMMMM + MMMML) and in fig. 6 this is not the case. But again, pre-deviant there couldn't be a real difference so MMMMM+MMMML should be quite similar to MMMML except for higher SNR in the former due to having more trials. Was a different filter applied maybe?
4. In fig. 5 the critical result is the lack of positive difference (P3) between infrequent and frequent sequences for N+ patients, when deviance is on the left (left bottom panel). However, it seems that the outstanding condition is the frequent (global standard) rather than the infrequent (left global deviant) response. The infrequent response displays a positivity starting at ~450ms as in all other groups and conditions. It is the frequent condition (global standard, MMMML+MMMMM) which is unusually positive here. This makes it difficult to conclude that there is no P3 to the infrequent global deviant in this condition.
5. The finding regarding the omissions (discussed on page 23, lines 32-55) is super interesting in my opinion. The implications are mind boggling – it suggests that the system has already learned (has a prior) the abnormal responses elicited by left side stimuli! What is worrying though is that we do not see comparable omission responses in the N- or HC groups. Can this be explained? Otherwise it makes one wonder about the omission response seen in the N+ patients in the frequent MMMMR condition.
6. Patients: It is not clear what were the criteria for including patients in the N+ group. Did they have to show abnormal results in all 6 tests or just in some?
7. Lesions: The lesion overlap figure shows overlap only in white matter. It would be a very selective group if there were no damage to the cortex in any patient. I guess it looks like this because the scale starts with 30% overlap. It would be more informative to start from 0 overlap to show the entire extent of the lesions. Also, it should be noted that the lesions of the N- patients must have been much smaller in volume, considering the negligible overlap.
8. Did the MRI normalization procedure take into account the lesioned tissue (see Brett et al., 2001). Please also report who delineated the lesion on the MRI.

9. I was confused about the procedure used to select the window of analysis (last paragraph of p. 11). If I correctly understood, the dependent variable in the main analysis of the MMN was the average amplitude in a fixed window of 170-220ms which was determined by performing a time resolved t-test with the Guthrie and Buchwald method for correction of multiple comparisons (this is described in the supplementary but should be described in the main text in my opinion). By my reading, the t contrast was between every point and the baseline. Was this done for every possible condition ({MMMMM, MMMMR, MMMML, MMMM_} X {frequent, infrequent} * {N+, N-, HC})? If so, how were the results combined to form a fixed ROI\window for the MMN across conditions? If not, what was the procedure exactly? This is essential to clarify to make sure the procedure doesn't bias (double-dipping) the main analysis. Finally, in the figures there are shadings for time windows of significant difference between conditions. Are these also based on the Guthrie and Buchwald method, and if so was there any control for multiple comparisons across electrodes (the method controls only across time) and how was 10 samples determined as the cutoff for significance?
Minor comments
1. Add error measures around the waveforms if possible.
2. Please add information about the stimuli (frequency content, envelope)
3. Was the patient's pure tone hearing level tested?
4. Information is missing about the order of the blocks and counterbalancing
5. The text says the SOA was 150 ms, while figure 1 says the ISI was 150ms. Looking at the ERP figures, it must be that the SOA was 150ms and the ISI was 100ms. But, the methods section also says that a trial lasted 850 ms – which is more consistent with ISI of 150 (and therefore SOA of 200ms). Please clarify, perhaps with adding the intervals in Figure 1?
6. The methods section only mentions an online filter of .01-60Hz. Was any filtering done during pre-processing of the data (the waveforms look very smooth for a 60 Hz low pass)?
7. Page 3 last line – Deouell et al. presented a continuous sequence of tones with interspersed standards and deviants. The 'four' is redundant in this sentence.
8. I suggest being a bit less conclusive about the underlying processes of ERP components (e.g. what the P3b marks, p. 4 line 41). We don't really know.
9. P. 8 line 6 – I suggest replacing 'blocked' by 'supported'.

Leon Deouell

Reviewer: 2 (Thomas Parr)

Comments to the Author
I am grateful for the opportunity to review this paper, in which the authors use a mismatch negativity paradigm, equipped with global in addition to local deviations, to investigate spatial neglect.

The design of the experiment is attractive in its simplicity, and in the clever use of omissions as a form of non-spatial deviant tone, contrasting with the left and right local deviants. The controls seem appropriate (both healthy controls, and patients with right brain lesions without neglect). I found the experiments and the discussion of the results convincing.

There were only two things I thought were missing. The first is reference to the excellent work by Martin Dietz and colleagues on this problem. For example https://doi.org/10.1523/JNEUROSCI.3765-13.2014, https://doi.org/10.1016/j.heares.2020.108052. These use a similar auditory paradigm in neglect patients, and apply dynamic causal modelling to interpret EEG responses in terms of effective disconnections. It strikes me that this is highly relevant to the authors' work and could be seen as offering a mechanistic physiological account of how the results in this study were generated.

I would suggest that the authors relate their results back to these papers in the discussion, and that they rephrase their sentence from the introduction '...deficits of predictive coding in patients with neglect have never been directly investigated...' to highlight that there are a few studies that do this.

The second thing I would suggest including is a sentence in the introduction to highlight that, by predictive coding, the authors are referring to predictive processing more broadly. The reason for this is that some of the computational papers the authors refer to are based upon discrete partially observed Markov decision processes, as opposed to the Kalman filter style models associated with predictive coding. The underlying maths is very similar, and the authors' results are equally applicable to both. The term 'predictive processing' is sometimes used as a broader term that encompasses both styles of modelling.

Finally, there is a typo on page 19 'frequent lef-side' (should be 'left').

Editorial Office comments

-Please supply a graphical abstract. A graphical abstract is a visual representation of the central finding or methodology of the paper. It will be displayed in the table of contents. Authors should strive to make the graphical abstract informative, interesting, visually appealing, and straightforward (no caption needed, no abbreviations except gene names). Please upload as a separate high resolution image file (tiff preferred). The technical requirements for the graphical abstracts are:
  --size of the submitted image: 1200 pixels square at 300dpi
  --font of the text: Arial 12-16 point (smaller fonts will not be legible online)

-Please add an Abbreviated Summary (up to 50 words) of your paper that captures the main purpose and conclusions of the work. The summary should state the main results written in the third person ('[1st author surname] et al. report that …') and conclusion. This summary will be used in the contents list online and should not contain any abbreviations other than those on the accepted abbreviations list and accepted gene/protein names, making it accessible to the non-specialist. Please upload it as a Word document. Allowed abbreviations: AIDS; ANOVA; AMPA; ATP; A,T,C,G; CNS; CSF; CT; DNA; ECG; EEG; EMG; GABA; HIV; MRI; NMDA; PET; PCR; RNA.
