# Supplementary material for: Deficits of hierarchical predictive coding in left spatial neglect
Source: Brain Commun. 2021 Jun 4;3(2):fcab111. doi: 10.1093/braincomms/fcab111 (PMC8209285; doi:10.1093/braincomms/fcab111)
Supplement: fcab111_Supplementary_Data [file Revision 1- Authors Response.pdf › Revision 1- Authors Response.pdf]

Point-to-point letter response to the reviewers for the Manuscript

BRAINCOM-2021-007.R1

Deficits of hierarchical predictive coding in left spatial neglect

In red text our replies

Reviewer: 1

Comments to the Author

The authors addressed most of the suggestions in my previous results. I believe the results are clearer now. I do have a few remaining questions:

We wish to thank the reviewer for his new suggestions that helped us improving the quality of our work.

1. The authors replaced Guthrie and Buchwald's method with a non-parametric FDR-controlled permutation. Non-parametric tests are an excellent choice, but because they are necessarily tailored to the question at hand, they require a full description of what has been done in each iteration (i.e. for each permutation) in order to build the surrogate distribution, and of how the procedure controls for multiple comparisons. For example, it wasn't clear to me whether the procedure included any consideration of temporal\spatial contiguity (as in the popular Maris and Oostenvledt (2007) procedure) and where did the false-discovery rate (FDR) come into play.

FDR-nonparametric permutation T-tests were performed with Brainstorm (Tadel et al. 2011; http://neuroimage.usc.edu/brainstorm) using the statistics section implemented in the software. In particular, this software performs non-parametric statistical inference that does not make assumptions on the distributions of the data (Maris and Oostenveld, 2007; Pantazis et al., 2005). Permutation tests were performed across subjects for random effects inference. Under the null hypothesis of no EEG-activity difference in the sensor data between two experimental conditions "A" and "B", in each participant the labels between conditions A and B are randomly permuted and the resulting data are used to compute a permutation t-statistic spatiotemporal sensor map. Repeating this permutation procedure 1000 times, using Monte Carlo random sampling, enables to estimate the empirical distribution of the t-statistic at each sensor and time point, and thus convert the original data into a p-value statistical map. Finally, to control for multiple comparisons across all sensors and time points, the p-values are adjusted using a false discovery rate procedure.

These informations are now detailed in the Supplementary Material on page 7 (this is signalled in the revised Method section of the main text).

Pantazis, D., Nichols, T. E., Baillet, S., & Leahy, R. M. (2005). A comparison of random field theory and permutation methods for the statistical analysis of MEG data. *Neuroimage*, *25*(2), 383-394.

2. On page 12, section "local effect", it seems that running the above procedure for each group and condition resulted in the same significant electrode cluster with the same time window, and that this spatio-temporal window was then used for the ANOVA. But in the figures, and in the supplementary table, the cluster of electrodes and temporal window is not the same across groups (compare the electrodes marked on the MMN topography for the N+ group on the right bottom panel of figure 4, with the electrodes marked for the MMN in the N- and HC group). Does this mean that the ROI was different for each condition? And if so, how was the ROI determined for conditions in which there was no significant effect?

In each group and for each condition, ROI (pools of electrodes) and time-windows used for the ANOVA and visualization purposes, were defined basing on the results of the FDR non-parametric permutation T-test (see also point 1). When no statistical effect was highlighted by the permutation test, which simply means that in any of the derivation and all along the entire epochs there was no difference between the two experimental conditions involved in the comparison, for sake of homogeneity in the analysis and in the figures, we considered the pools of electrodes that for the same between-experimental conditions comparison was significant in the same group, or in the N- groups. To give you an idea in more detail:

Fig.3 → In the N+ (bottom-left panel), permutation test failed to highlight a significant difference between MMMMM and MMMML during the time windows compatible with the MMN presence (~130-200 ms). In this case, we selected the same anterior pools in which permutation test showed significant difference between the same conditions in the N- and HC (Fz – Fc1 – Fcz – Fc2)

Fig.4 → In the N+ (bottom-left panel), during the MMN time windows (~130-200 ms). permutation test highlights a significant difference between Freq MMMML and Infreq MMMMR though in the same group, shows no significant difference between Freq MMMML and Infreq Omissions. In this case, we selected the same anterior pools in which permutation test highlights the presence of a significant difference (Fc5 – Fc3 – Fc1 – C3).

Fig.5 → In the N+ (bottom-left panel), permutation test failed to highlight a significant difference between Freq and Infreq conditions during the time windows compatible with the P3b presence (~450-750 ms). In this case, we selected the same posterior pools in which permutation test showed significant difference between the same conditions in the N- (Pz – P2 – POz – PO4).

Fig.6 → In the N- (middle-left panel), during the P3b time windows (~450-750 ms). permutation test highlights a significant difference between Freq MMMML and Infreq MMMMR though in the same group, shows no significant difference between Freq MMMML and Infreq Omissions. In this case, we selected the same pools in which permutation test highlights the presence of a significant difference (P5 – P3 – PO7 – PO5). Similarly, in the N+ (bottom-left panel) permutation tests resulted as non-significant both in the Freq MMMML vs. Infreq MMMMR and in the Freq MMMML vs. Infreq Omissions comparisons. In both cases, we selected the same posterior pools in which permutation test showed significant difference between the same conditions in the N- (P5 – P3 – PO7 – PO5).

This procedure is now better specified in the Supplementary material.

3. I couldn’t find Table 2 in the manuscript.

Sorry, in the main text we mistakenly defined **Supplementary Table 1** as **Table 2**: this is corrected in the revision.

4. Figure 5 bottom right panel shows apparently no P3b for (MMMMM+MMMML) when they are infrequent compared to frequent for the N+ group. However, it seems like the entire infrequent waveform (bottom right panel) is shifted downwards, starting around -200ms. Note especially the negative shift between 200-600ms, absent in other conditions. This raises some doubts about whether the P3 is really obliterated. It would be more convincing and highly informative I think to present the lines for the frequent\infrequent MMMMM and MMMML in separate lines (and similarly of course for frequent\infrequent MMMMM and MMMMR). This will allow the reader to understand where the effect comes from.

Maybe the reviewer refers to bottom-left panel in **Fig.5** rather than bottom-right which instead shows the MMMMM+MMMMR comparison.

As for the case of the bottom-left panel, we would like to emphasize that the FDR-nonparametric permutation T-tests highlighted no significant difference between the two waveforms in any of the

derivations and time-points. This means that the negativity the reviewer is dealing about is statistically not significant and represents a random deflection rather than a systematic shift in the waveforms (please note that no visual P3 in response to rare invalidly cued left-side targets, was also reported in Lasaponara et al., 2018, and that the same type of targets evoke no Pupil Dilation, i.e. a marker of infrequency detection, comparable to that evoked by right-side invalid targets, see Lasaponara et al., 2021).

Lasaponara, S., D'Onofrio, M., Pinto, M., Dragone, A., Menicagli, D., Bueti, D., ... & Doricchi, F. (2018). EEG correlates of preparatory orienting, contextual updating, and inhibition of sensory processing in left spatial neglect. Journal of Neuroscience*, 38(15), 3792-3808.*

Lasaponara, S., Fortunato, G., Conversi, D., Pellegrino, M., Pinto, M., Collins, D. L., ... & Doricchi, F. (2021). Pupil dilation during orienting of attention and conscious detection of visual targets in patients with left spatial neglect. Cortex*, 134, 265-277.*

As further support to non-significance of the negative deflection highlighted by the reviewer, we also would like to note that: 1) there are no differences in the baseline-correction period (-800 to -600) between the two waveforms; 2) some shifts between the two waveforms could be observed also in the opposite direction (Infreq more positive than Freq, for example -600 to -400 ms) and finally, 3) consistently with what is observed also in HC and N-, in the N+ the positive peak occurring at 200 msec has the same amplitude for both Freq and Infreq conditions. A shift to the entire waveforms toward the positive polarity imply that the same peaks, differently to what is observed in the two control groups, would have been higher in the Infreq condition compared to the Freq one.

As for the request of presenting lines for the frequent\infrequent MMMMM and MMMML in separate lines (and similarly of course for frequent\infrequent MMMMM and MMMMR), here we report a figure with the data of the neglect group. These show that the non-significant negativity highlighted by the reviewer is present both for infrequent MMMMM and infrequent MMMML.

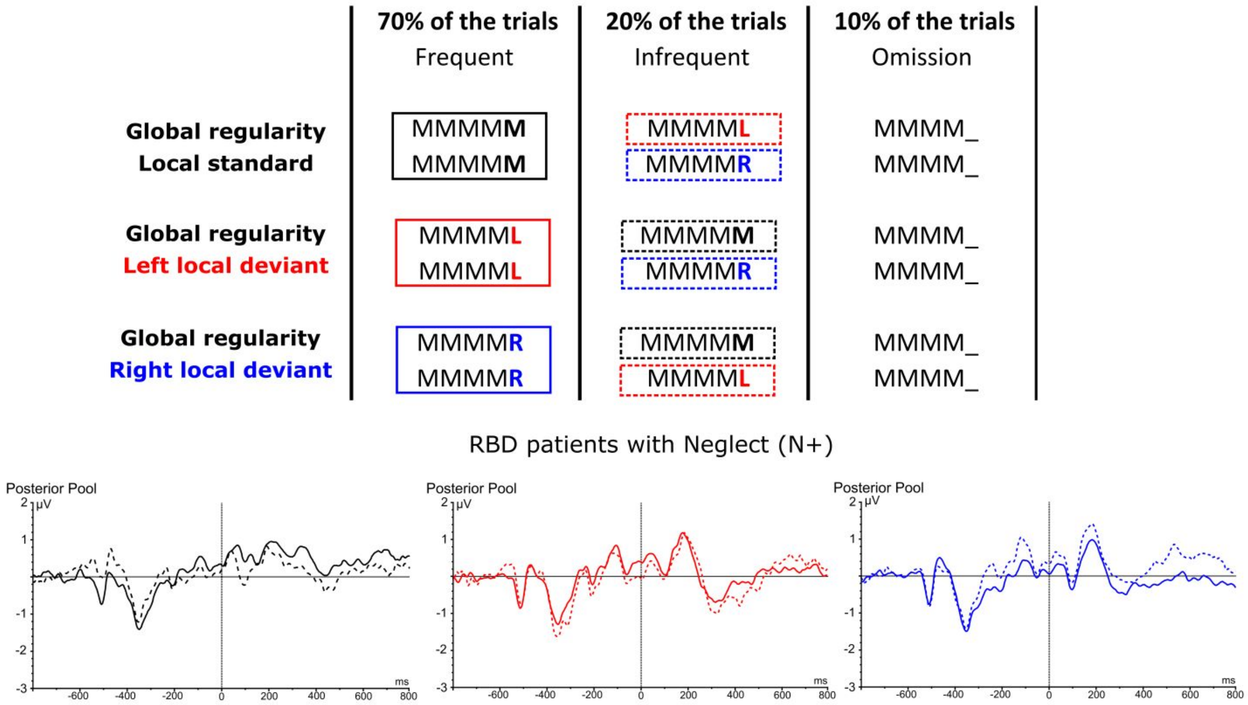

We respectfully see no relevant reason to overcharge the manuscript with additional figures (and analyses) that are related to a statistically non-significant effect resulting by the very EEG analyses recommended by the reviewer in his first review round.

5. I would recommend discussing the limitations and less explainable results of the study. Some examples include the big difference in lesion size between the N- and N+ groups

Past anatomical studies have homogenously pointed out that lesion size is usually larger in N+ than in N-, though the same studies show that lesion size is not a critical factor inducing neglect (see for example in Thiebaut de Schotten et al., 2012)

[…], and the somewhat unusual sparing of the cortex in the N+ group.

Please note that **Supplementary Fig. 2** highlights lesions in cortical areas. Besides, all current anatomical studies have crucially pointed out that lesions producing neglect overlap in subcortical parietal-frontal white matter rather than in parietal-frontal cortical areas (reviews in Bartolomeo et al. 2007; Doricchi et al., 2008).

A result which does not easily fall into place is the lack of omission responses in the N- and HC groups.

This is a very interesting point that allowed us to enrich the revised discussion section with a dedicated new paragraph (page 24-25 in the revision). MMN to omissions is a classical finding that, by the way, highlights the predictive rather than "sensory adaptation to repetition" nature of the MMN (see Wacongne et al. 2011, for a brief review of this point). Nonetheless, we would like to note that MMN to omissions is usually observed in paradigms where tone-pitch, rather than tone-position, is the salient stimulus feature in the task. In our task, the relevant stimulus feature is tone-position. This implies that while in tone pitch-paradigms a no-tone omission is treated as a change in pitch, in tone position paradigms a no-tone omission is not treated as a change in tone position. We have checked the existing literature to see whether other authors have used omission-trials in MMN studies where tone-position was the critical stimulus feature. Based on inspection of records on PubMed and GScholar together with an authoritative review of the literature by Naatanen et al., 2007, we have found no previous study that included omission tone trials in tone-position MMN paradigm.

There is also the fact that the omissions failed to elicit a P3b for omissions vs. an MMMMM sequence in the neglect patients (even though no lateral stimuli were involved).

The explanation for this result might be quite obvious: since in patients with Neglect, the subjective acoustic straight-ahead was shifted 17.4° to the right of the objective acoustic straight-ahead MMMM**M** stimuli were perceived as coming from the left neglected side of space, therefore unable to elicit an evaluation of global deviance just as in the case of the condition with frequent MMMML (see bottom-left panel of **Fig.6**)

[…] I do not mean to imply that all results must be easy to explain, only that it should be noted perhaps.

We have tried to provide likely interpretations of new empirical findings and, see for example the end of the new paragraph on page 25, we have cautiously noted that "*Future studies should address and clarify these points*"

Reviewer: 2

Comments to the Author
I am grateful to the authors for the changes they have made, and have no further suggestions.

We wish to thank the reviewer for her/his suggestions which helped us improving the quality of our work.
