# Supplementary material for: Deficits of hierarchical predictive coding in left spatial neglect
Source: Brain Commun. 2021 Jun 4;3(2):fcab111. doi: 10.1093/braincomms/fcab111 (PMC8209285; doi:10.1093/braincomms/fcab111)
Supplement: fcab111_Supplementary_Data [file Revision 1-Decision Letter.pdf › Revision 1-Decision Letter.pdf]

Revision 1-Decision Letter

30-Mar-2021

Dear Dr. Doricchi,

Thank you for your recent submission BRAINCOM-2021-007.R1
entitled "Deficits of hierarchical predictive coding in left spatial neglect" to Brain Communications.

Your manuscript has been reviewed by the same reviewers who read the original paper, and you will see that one of the reviewers has a few remaining constructive critiques that I think you could address in a minor revision of the manuscript. Therefore, I invite you to respond to the reviewer(s)' comments and revise your manuscript. The reviewer(s)' comments are included at the bottom of this message.

In addition to the reviewers' comments, please note our editorial comments added below to comply with our requirements for presentation of scientific data at Brain Communications.

Please format your revised manuscript according to our Instructions to Authors https://academic.oup.com/braincomms/pages/General_Instructions – a summary of key points is attached to help with this.

To revise your manuscript, click the link below or log into https://mc.manuscriptcentral.com/braincom and enter your Author Centre, where you will find your manuscript title listed under "Manuscripts with Decisions." Under "Actions," click on "Create a Revision." Your manuscript number will be appended to denote a new revision.

*** PLEASE NOTE: This is a two-step process. After clicking on the link, you will be directed to a webpage to confirm. ***

https://mc.manuscriptcentral.com/braincom?URL_MASK=abf73c7e8c834cbd9deb0a28f444f6e3

Please upload a revised manuscript with changes tracked using track changes or in coloured text.

When submitting your revised manuscript, you will be asked to respond to the reviewer(s) comments. Please be as specific as possible in your response to all of the comments.

The editorial team at Brain Communications also aims to increase transparency in scientific publishing and to demonstrate the value that peer review brings to the scientific process. Please therefore consider whether you are willing to have reviewer comments on your paper and your response published as supplementary data with the paper if it is accepted. This is entirely optional and will only be done if both the authors and reviewers agree. When submitting your revision you will have the opportunity to update the answer you entered previously.

IMPORTANT: Your original files are available to you when you upload your revised manuscript. Please delete any redundant files before completing the submission.

Because we are trying to facilitate timely publication of manuscripts submitted to Brain Communications, we would appreciate your revised manuscript as soon as possible. If it is not possible for you to resubmit within 3 months, the option to submit a revision will expire, and we would then consider your revised paper as a new submission. However, if you need a little more time, please get in touch and we can extend this deadline for you.

Once again, thank you for submitting your manuscript to Brain Communications and I look forward to receiving your revision.

Yours sincerely,

Prof. Tara Spires-Jones
Editor, Brain Communications

Reviewer(s)' Comments to Author:

Reviewer: 2 (Thomas Parr)

Comments to the Author
I am grateful to the authors for the changes they have made, and have no further suggestions.

Reviewer: 1 (Leon Deouell)

Comments to the Author
The authors addressed most of the suggestions in my previous results. I believe the results are clearer now. I do have a few remaining questions:
1. The authors replaced Guthrie and Buchwald's method with a non-parametric FDR-controlled permutation. Non-parametric tests are an excellent choice, but because they are necessarily tailored to the question at hand, they require a full description of what has been done in each iteration (i.e. for each permutation) in order to build the surrogate distribution, and of how the procedure controls for multiple comparisons . For example, it wasn't clear to me whether the procedure included any consideration of temporal\spatial contiguity (as in the popular Maris and Oostenvledt (2007) procedure) and where did the false-discovery rate (FDR) come into play.
2. On page 12, section "local effect", it seems that running the above procedure for each group and condition resulted in the same significant electrode cluster with the same time window, and that this spatio-temporal window was then used for the ANOVA. But in the figures, and in the supplementary table, the cluster of electrodes and temporal window is not the same across groups (compare the electrodes marked on the MMN topography for the N+ group on the right bottom panel of figure 4, with the electrodes marked for the MMN in the N- and HC group). Does this mean that the ROI was different for each condition? And if so, how was the ROI determined for conditions in which there was no significant effect?
3. I couldn't find Table 2 in the manuscript.
4. Figure 5 bottom right panel shows apparently no P3b for (MMMMM+MMMML) when they are infrequent compared to frequent for the N+ group. However, it seems like the entire infrequent waveform (bottom right panel) is shifted downwards, starting around -200ms. Note especially the negative shift between 200-600ms, absent in other condiditions. This raises some doubts about whether the P3 is really obliterated. It would be more convincing and highly informative I think to present the lines for the frequent\infrequent MMMMM and MMMML in separate lines (and similarly of course for frequent\infrequent MMMMM and MMMMR). This will allow the reader to understand where the effect comes from.
5. I would recommend discussing the limitations and less explainable results of the study. Some examples include the big difference in lesion size between the N- and N+ groups, and the somewhat unusual sparing of the cortex in the N+ group. A result which does not easily fall into place is the lack of omission responses in the N- and HC groups. There is also the fact that the omissions failed to elicit a P3b for omissions vs. an MMMMM sequence in the neglect patients (even though no lateral stimuli were involved). I do not mean to imply that all results must be easy to explain, only that it should be noted perhaps.
